# Targeting epiregulin in the treatment-damaged tumor microenvironment restrains therapeutic resistance

**DOI:** 10.1038/s41388-022-02476-7

**Published:** 2022-10-06

**Authors:** Changxu Wang, Qilai Long, Qiang Fu, Qixia Xu, Da Fu, Yan Li, Libin Gao, Jianming Guo, Xiaoling Zhang, Eric W.-F. Lam, Judith Campisi, Yu Sun

**Affiliations:** 1 CAS Key Laboratory of Tissue Microenvironment and Tumor, Shanghai Institute of Nutrition and Health, University of Chinese Academy of Sciences, Chinese Academy of Sciences, Shanghai, 200031 China; 2 Department of Urology, Zhongshan Hospital, Fudan University, Shanghai, 200032 China; 3 Department of Pharmacology, Institute of Aging Medicine, Binzhou Medical University, Yantai, 264003 Shandong China; 4 Central Laboratory for Medical Research, Shanghai Tenth People’s Hospital, Tongji University School of Medicine, Shanghai, 200072 China; 5 Department of Orthopedic Surgery, Xinhua Hospital, Shanghai Jiao Tong University School of Medicine, Shanghai, 200092 China; 6 Department of Surgery and Cancer, Imperial College London, London, W12 0NN UK; 7 Buck Institute for Research on Aging, Novato, CA 94945 USA; 8 Lawrence Berkeley National Laboratory, University of California, Berkeley, CA 94720 USA; 9 Department of Medicine and VAPSHCS, University of Washington, Seattle, WA 98195 USA

**Keywords:** Cancer microenvironment, Diagnostic markers

## Abstract

The tumor microenvironment (TME) represents a milieu enabling cancer cells to develop malignant properties, while concerted interactions between cancer and stromal cells frequently shape an “activated/reprogramed” niche to accelerate pathological progression. Here we report that a soluble factor epiregulin (EREG) is produced by senescent stromal cells, which non-cell-autonomously develop the senescence-associated secretory phenotype (SASP) upon DNA damage. Genotoxicity triggers EREG expression by engaging NF-κB and C/EBP, a process supported by elevated chromatin accessibility and increased histone acetylation. Stromal EREG reprograms the expression profile of recipient neoplastic cells in a paracrine manner, causing upregulation of MARCHF4, a membrane-bound E3 ubiquitin ligase involved in malignant progression, specifically drug resistance. A combinational strategy that empowers EREG-specific targeting in treatment-damaged TME significantly promotes cancer therapeutic efficacy in preclinical trials, achieving response indices superior to those of solely targeting cancer cells. In clinical oncology, EREG is expressed in tumor stroma and handily measurable in circulating blood of cancer patients post-chemotherapy. This study establishes EREG as both a targetable SASP factor and a new noninvasive biomarker of treatment-damaged TME, thus disclosing its substantial value in translational medicine.

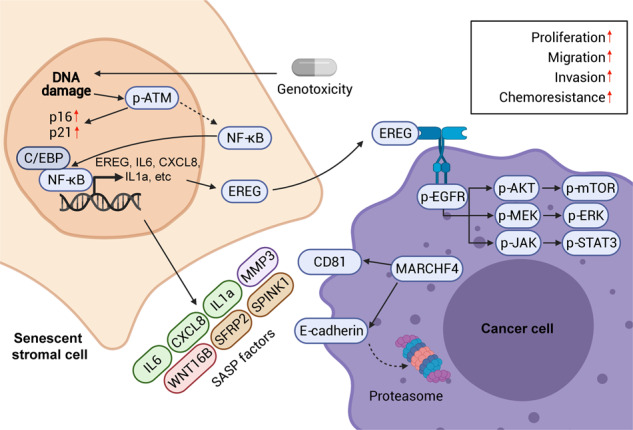

## Introduction

The tumor microenvironment (TME) plays a key role in host response to clinical intervention and substantially affects therapeutic outcomes [1–3]. Accurately deciphering the tumor-stroma interplay “cryptograms” in the microenvironment helps improve the practical tailoring and fine-tuning of therapeutic strategies. However, to date, comprehensive and insightful studies that assess cancer cells and their adjacent niches as a whole remain limited, are often unstructured and lack efficient models. An accessible transcriptomic analysis platform for functional appraisal of the TME to identify key factors that mediate tumor-stroma interactions is highly desired, which allows in-depth evaluation of the TME–supported actions. Further, transcriptomic profiles shaped by paracrine effectors from activated stroma integrated with genomic data of cancer cells from pan-cancer studies will allow a clear visualization, which can show a planetary view of diseases through a comprehensive tumor portrait.

Cellular senescence is a state of essentially irreversible cell cycle arrest, wherein cells remain metabolically active but do not respond to mitogenic stimuli. They display enhanced activities of the lysosomal enzyme, namely senescence-associated β galactosidase (SA-β-Gal), and increased expression of the tumor suppressor p16^INK4a^ and p21^CIP1^, typical biomarkers of cellular senescence and widely used for in vitro and in vivo assays [4]. Senescence inducers frequently cause DNA damage, forming DNA damage foci known as DNA segments with chromatin alterations reinforcing senescence and often manifested as senescence-associated heterochromatin foci [5]. Of note, senescent cells secrete a plethora of cytokines, chemokines, growth factors and proteases that profoundly affect neighboring cells, a phenomenon termed the senescence-associated secretory phenotype (SASP) [6]. In cancer clinics, cellular senescence induced by chemotherapy and/or irradiation is referred to as therapy-induced senescence (TIS) [7]. Although anticancer therapies per se are designed to induce apoptosis of cancer cells, their surrounding stromal cell counterparts are also affected, the latter developing TIS and capable of influencing the microenvironment through expression of SASP factors [8, 9]. The SASP exerts a series of pathophysiological effects via secretion of proteins that may signal back to the receptors on their own cell surface (cell-autonomous), or on the surface of other cells (non-cell-autonomous), with the complexity further increased by the differential effects a single protein can exert in either a cell-autonomous or a non-cell-autonomous manner, or both [4].

In the spectrum of soluble factors released by senescent human stromal cells developing the SASP, we noticed that EREG, a member of the epidermal growth factor (EGF) family of secreted peptides, emerges on the top ranking SASP expression list [1]. Beyond genomic/epigenetic alterations of oncogenes and/or tumor suppressors, the epidermal growth factor receptor (EGFR) ligand EREG can function as a bona fide biomarker of therapeutic sensitivity for many EGFR-driven carcinomas [10]. Among diverse EGFR ligands, EREG significantly reduces cellular sensitivity to tyrosine kinase inhibitors and is associated with decreased response to targeted agents, providing a basis for clinical decision making [10]. Further, increased expression of EREG in cancer-associated fibroblasts also deserves attention, as it correlates with higher tumor stage, enhanced invasiveness and shorter overall survival of cancer patients [11–13]. However, the mechanism underlying treatment-inducible expression of EREG in human stroma and its pathological implications remain poorly defined. In this study, we addressed several fundamental but hitherto-unknown aspects of stromal EREG in anticancer treatment background and established its correlation with acquired resistance of cancer cells. Overall, the data establish EREG as both a tumor-promoting factor that is targetable to avert disease exacerbation and a circulating biomarker exploitable to monitor the host response to therapeutic agents in cancer clinics.

## Results

### Genotoxicity induces EREG expression in human stromal cells

**Fig. 1:**
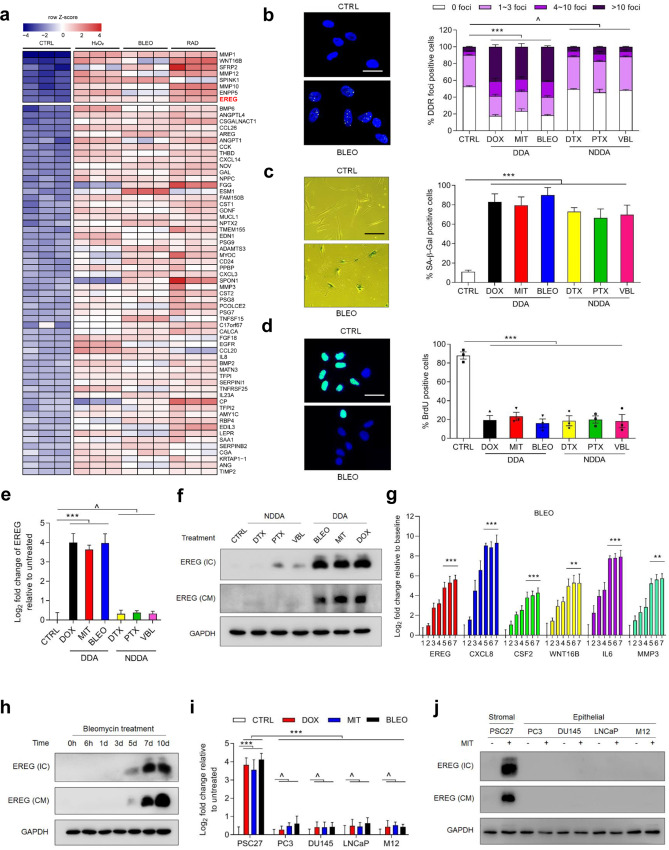
Genotoxicity induces expression of EREG and other secreted factors of the SASP spectrum in human stromal cells. **a** Transcriptome-wide profiling of gene expression changes in primary normal human prostate stromal cell line (PSC27) by microarray. Cell lysates were collected for analysis 7 days after treatment. CTRL control. H_2_O_2_ hydrogen peroxide. BLEO bleomycin. RAD radiation. Red highlighted, EREG. Agilent microarray data adapted from Sun et al. with permission from *Nature Medicine*, copyright 2012, Springer Nature [1]. **b** Representative immunofluorescence staining images (γH2AX and p-53BP1 co-staining, left) and comparative statistics (right) of DNA damage response (DDR) in PSC27 cells treated by DOX (doxorubicin), MIT (mitoxantrone), BLEO (bleomycin), DTX (docetaxel), PTX (paclitaxel) and VBL (vinblastine). DDA DNA-damaging agents (DDAs). NDDA non-DNA-damaging agents. DDR were classified into four sub-categories including 0 foci, 1–3 foci, 4–10 foci and >10 foci per cell. Scale bars, 15 μm. **c** SA-β-Gal staining of PSC27 cells treated by various agents used in **b**. Cells were stained 7 days after in vitro treatments. Scale bars, 30 μm. Right, comparative statistics. **d** BrdU staining of stromal cells treated by different agents as indicated in **b** and **c**. Scale bars, 15 μm. Right, comparative statistics. **e** Quantitative RT-PCR of EREG expression after treatment of PSC27 cells by various agents. Cell lysates were collected for measurement 7 days after treatment. Signals normalized to CTRL. **f** Immunoblot analysis of EREG expression in stromal cells 7 days after treatments performed as indicated. IC intracellular samples. CM conditioned media. GAPDH, loading control. **g** Time course expression assessment of a subset of EREG and other typical SASP factors (CXCL8, CSF2, WNT16B, IL6 and MMP3) after drug treatment of stromal cells in vitro. Numeric numbers indicate the individual days after treatment. **h** Immunoblot measurement of EREG expression at the protein level in the time course described in **g**. **i** Comparative appraisal of EREG transcript expression in stromal cells (PSC27) versus cancer epithelial cells (PC3, DU145, LNCaP and M12). Signals normalized to untreated sample per cell line. **j** Immunoblot assessment of EREG expression in protein lysates of stromal and epithelial cells after bleomycin treatment as performed in **i**. Data are representative of three independent experiments. ^*p* > 0.05, *p < 0.05, **p < 0.01, ***p < 0.001. *p* values were calculated by Student’s *t* test (**c**–**e**, **g**) and two-way ANOVA (**b**, **i**). ^*p* > 0.05, *p < 0.05, **p < 0.01, ***p < 0.001.

Gene-specific alterations are not the sole determinants that can precisely direct the use of targeted therapies, the efficacy of which in cancer patients is largely unpredictable due to intrinsic genetic complexity and variable tissue context. Specifically, molecular profiling of negative predictors of response to anti-EGFR antibodies such as cetuximab and panitumumab, covers regulators of the MAPK and PI3K/AKT signaling pathways and mutations in *NRAS*, *BRAF*, *PIK3CA* and *PTEN* [14]. However, the vast majority of EGFR-associated cancer research has been focused on cancer cells per se, leaving the host-resident stroma largely overlooked. We recently noticed that a prostate stromal cell line PSC27, composed mainly of fibroblasts but with minor percentage of other stromal cell lineages including endothelial and immune cells of the TME, secrets a large array of SASP factors after exposure to cytotoxicity particularly those generated by genotoxic chemotherapy or ionizing radiation [1]. Among diverse genes, EREG emerged as one of the most upregulated SASP components as revealed by bioinformatics (Fig. 1a) [1], largely consistent with other reports [15–17]. Despite a number of EREG-related studies mainly focusing on the consequence of cancer cell-expressed ligands [18, 19], it remains unknown whether and how senescent stromal cell-derived EREG functions as a cancer-responsive factor in the TME niche, specifically regarding the potential of stromal EREG in driving malignant progression of human cancers. To address these issues, we chose to further investigate EREG. First, we expanded the experiment by employing a subset of DNA-damaging agents (DDAs) including doxorubicin (DOX), mitoxantrone (MIT) and bleomycin (BLEO), to treat human stromal cells. We found a substantially enhanced number of DNA damage response (DDR) foci (γH2AX and p-53BP1 co-staining), increased lysosomal activity (SA-β-Gal) and inhibited DNA synthesis (Fig. 1b–d), indicative of typical cell cycle arrest companied by cellular senescence. Interestingly, effects caused by these DDAs markedly differed from those generated by non-DNA-damaging agents (NDDAs) such as docetaxel (DTX), paclitaxel (PTX) and vinblastine (VBL), which typically disturb microtubule structural organization [20]. Subsequent examination at both mRNA and protein levels confirmed an inducible expression nature of EREG in response to DDAs (*p* < 0.001 at transcript level), a feature not phenocopied by NDDAs (Fig. 1e, f). Further, the expression pattern of EREG largely resembled that of other hallmark SASP factors including CXCL8, CSF2, WNT16B, IL6 and MMP3, which is characterized by a gradual increment until cells entered a platform within 7–10 days after genotoxic treatment (*p* < 0.001 for EREG/CXCL8/CSF2/IL6, *p* < 0.01 for WNT16B/MMP3) and consistent with protein level changes (Fig. 1g, h). Expression analysis with lysates from senescent cells induced by oncogenic activation of HRAS^G12V^ (oncogene-induced senescence (OIS)) further substantiated the time course tendency of EREG expression and secretion (Supplementary Fig. 1a, b).

We next assessed EREG expression among a handful of cell lines of prostate origin, and found that stromal cells were significantly more inducible for EREG than epithelial cells, implying a special mechanism supporting EREG production in stromal cells upon genotoxic insults (*p* < 0.001 for stromal, *p* > 0.05 for epithelial cancer lines) (Fig. 1i, j). Such a characteristic expression pattern was subsequently confirmed in several cell lines of human lung origin, including a stromal line HFL1 and several carcinoma cell lines regardless of malignancy, suggesting an organ- or tissue type-independent nature of EREG induction (Supplementary Fig. 1c–h). Genomic instability is a critical hallmark of most cancer cells, which usually exhibit defects in activation of DDR pathways [21], a case that may partly explain the differential response to DNA damage between stromal and cancer epithelial cells as observed in these assays.

Gene expression profiling interactive analysis based on tumor and normal samples archived in the TCGA and the GTEx databases suggested a preferentially higher expression of EREG in human tumor specimens than their normal tissue controls for most cancer types (Supplementary Fig. 1i). However, given the highly inducible pattern of EREG we observed in the stromal cell populations upon genotoxic insults as revealed by in vitro assays, it is reasonable to further investigate the clinical significance of this SASP factor in further depth, specifically in therapeutic settings.

### Stromal EREG expression correlates with adverse clinical outcomes

**Fig. 2:**
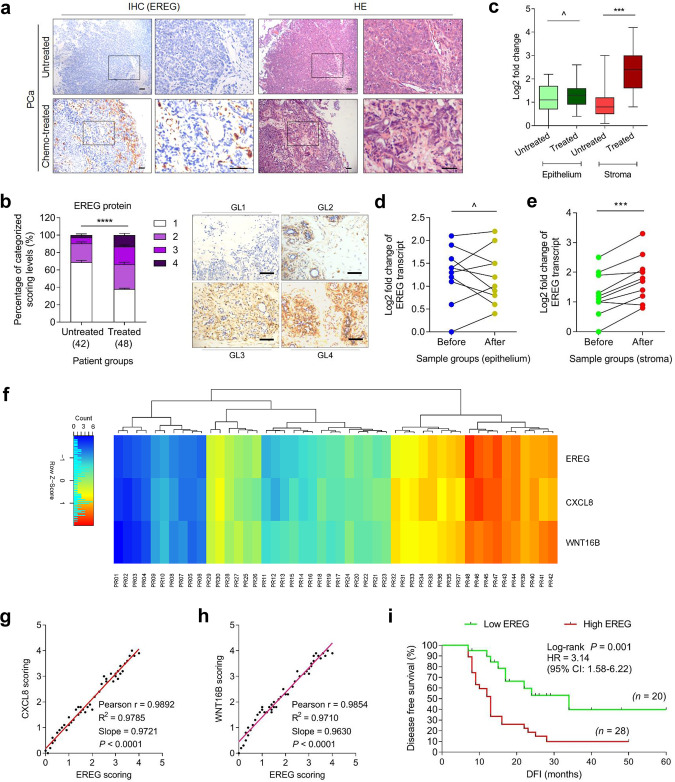
EREG is expressed in human prostate stroma after chemotherapy and correlates with adverse survival posttreatment. **a** Representative images of EREG expression in sample of human prostate cancer patients after histological examination. Left, immunohistochemical (IHC) staining. Right, hematoxylin and eosin (HE) staining. In each staining set, top tissues, untreated; bottom tissues, chemo-treated (mitoxantrone, MIT). Rectangular regions selected in the left images per staining are amplified into the right images. Scale bars, 100 μm. **b** Pathological assessment of stromal EREG expression in PCa samples (untreated, 42 patients; treated, 48 patients). Patients were pathologically assigned into four categories per IHC staining intensity of EREG in the stroma. 1, negative; 2, weak; 3, moderate; 4, strong expression. Left, statistical comparison of the percentage of each category. Right, representative images of each category regarding EREG signals. GL grading level. Scale bars, 100 µm. **c** Boxplot summary of EREG transcript expression by qRT-PCR analysis upon laser capture microdissection (LCM) of cells from tumor and stroma, respectively. Signals normalized to the lowest value in the untreated epithelium group, with comparison performed between untreated (42 patients) and treated (48) samples per cell lineage. For cells of either epithelium or stroma origin, samples from ten patients out of untreated and treated groups were randomly selected for further analysis and parallel comparison. **d** Comparative analysis of EREG expression at transcription level between epithelial cells collected before and after chemotherapy (MIT). Each dot represents an individual patient, with the data of “before” and “after” connected to allow direct assessment of EREG induction in the same individual patient. **e** Comparative analysis of EREG expression at transcription level between stromal cells collected before and after chemotherapy. Presentation follows the manner described in **d**. **f** Pathological correlation between EREG, CXCL8 and WNT16B in the stroma of PCa patients after treatment. Scores were from the assessment of molecule-specific IHC staining, with expression levels colored to reflect low (blue) via modest (turquoise) and fair (yellow) to high (red) signal intensity. Columns represent individual patients, rows different SASP factors. Totally 48 patients treated by chemotherapy were analyzed, with scores of each patient averaged from three independent pathological readings. **g** Statistical correlation between EREG and CXCL8 scores in the 48 tumors with matching protein expression data. **h** Statistical correlation between EREG and WNT16B scores in the same group of tumors described in **g**. **i** Kaplan–Meier analysis of PCa patients. Disease-free survival (DFS) stratified according to EREG expression (low, average score <2, green line, *n* = 20; high, average score ≥2, red line, *n* = 28). DFS represents the length (months) of period calculated from the date of PCa diagnosis to the point of first time disease relapse. Survival curves generated according to the Kaplan–Meier method. HR hazard ratio. Data in all bar plots are shown as mean ± SD and representative of three biological replicates. *p* values were calculated by Student’s *t* test (**c**–**e**), two-way ANOVA (**b**), Pearson test (**g**, **h**) and log-rank (Mantel–Cox) test (**i**). ^*p* > 0.05, ***p < 0.001, ****p < 0.0001.

Data from cell-based experiments prompted us to further determine whether EREG is produced by the benign components of TME, a pathological entity that underlies the progression of multiple cancer types. We first examined the specimens of a cohort of prostate cancer (PCa) patients, who developed primary tumors and underwent chemotherapy involving the genotoxic drug MIT. Immunohistochemistry (IHC) indicated that EREG was markedly expressed in the prostate tissues after chemotherapy, but not before (Fig. 2a). Consistent with our in vitro data, EREG was preferentially expressed in the stroma, in contrast to the surrounding epithelium which displayed limited or no staining (Fig. 2a).

We then stratified in vivo EREG signals in patient tissues with a pre-determined pathological appraisal procedure that allowed quantitative assessment of a target protein expression according to its IHC staining intensity, and found a striking induction pattern of EREG post- versus pre-chemotherapy (*p* < 0.0001) (Fig. 2b). Transcript profiling upon laser capture microdissection (LCM) of cell lineages isolated from primary tissues substantiated EREG induction in the stromal rather than epithelial cell populations (*p* < 0.001 versus *p* > 0.05) (Fig. 2c). To further establish the in vivo inducibility of EREG, we randomly selected a subset of patients whose pre- and post-chemotherapy samples were both available, and found remarkably upregulated EREG in the stroma, but not epithelium, of each individual post-chemotherapy (Fig. 2d, e). Importantly, we noticed the induction pattern of EREG in the damaged TME seemingly synchronized with that of CXCL8 and WNT16B, two canonical SASP factors of human stroma cells (Fig. 2f) [1, 22, 23]. The correlation between EREG and CXCL8 or WNT16B expression in the damaged TME was further supported by pathological appraisal of their expression in post-treatment patients (Fig. 2g, h). Of note, Kaplan–Meier analysis of PCa patients stratified according to EREG levels in the stromal compartments of their TME suggested a significant but negative correlation between stromally expressed EREG and disease-free survival (DFS) of chemo-treated patients (*p* = 0.001, log-rank test) (Fig. 2i).

The distinctive pathological landscape of EREG was reproduced by an extended study that recruited a cohort of human breast cancer (BCa) patients (*p* = 0.0117 for BCa by log-rank test) (Supplementary Fig. 2a–i). Of note, Cox proportional hazard regression analyses of these patients indicated significant correlation of stromal EREG with poor cancer survival (Supplementary Tables 1 and 2). Together, our data implied the potential of EREG expression intensity in tumor stroma as an SASP-specific and tumor-independent predictor in clinics, which can be used to stratify the risk of disease relapse and clinical mortality in post-treatment patients, and suggested a likely causal role of stromal EREG in tumor progression.

### EREG induction is primarily mediated by the NF-κB complex and supported by epigenetic remodeling

**Fig. 3:**
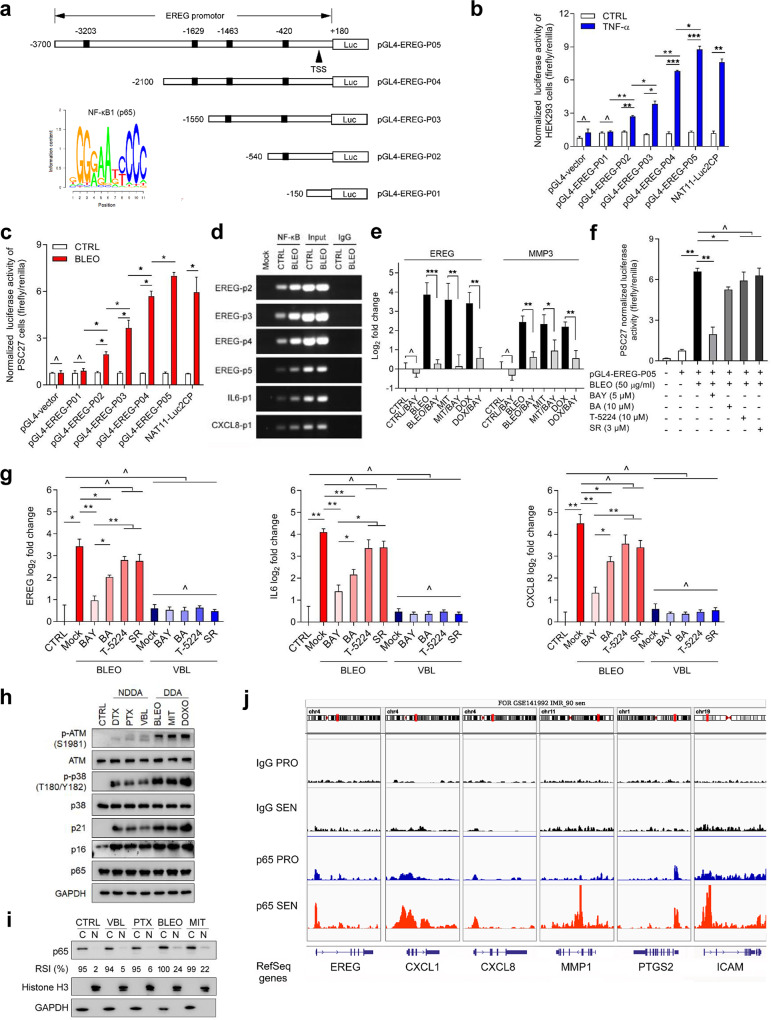
DNA damage induces EREG expression in stromal cells via regulation by the NF-κB complex, C/EBP axis and epigenetic modifications. **a** Schematic of putative NF-κB binding sites in the proximal region of EREG promoter. A set of reporter constructs was generated by sequential cloning of the promoter fragments into a pGL4.22 vector (pGL-EREG-P01 to P05) that expresses firefly luciferase. Numeric numbers on the top denote the core site of each putative NF-κB binding motif, while numbers at the left mark the length of each segmental promoter clone. TSS transcription start site. Lower-left inlet, consensus binding motif of the NF-κB subunit p65. **b** Assessment of luciferase activities upon exposure of 293F cells pre-transfected with the individual EREG promoter constructs to TNF-α at 40 ng/ml in culture. The empty vector was used as a negative control, while a construct NAT11-Luc2CP encoding multiple copies of typical NF-κB binding sequences and an optimized IL-2 minimal promoter served as a positive control. Signals were presented as relative ratios of firefly/renilla luciferase activities. **c** Luciferase activity assay with lysates of PSC27 cells pre-transfected with each of the constructs used in **b** prior to treatment by 50 μg/ml bleomycin (BLEO) in culture. **d** Chromatin immunoprecipitation (ChIP) was performed to identify potential NF-κB binding sites in the proximal promoter of EREG. Left, EREG-p2/p3/p4/p5 denotes four representative genomic sites in EREG promoter region, while selective NF-κB binding sites from IL6 and CXCL8 served as positive controls. **e** EREG and MMP3 transcript expression in PSC27 cells exposed to BLEO, MIT (mitoxantrone) or DOX (doxorubicin), with or without the NF-κB inhibitor BAY (Bay 11-7982, 5 μM). Signals were normalized to untreated cells, with MMP3 expression analyzed as positive control. **f** The reporter construct pGL-EREG-P05 was transiently transfected into PSC27 cells before treatment by BLEO. BAY (5 μM), BA (betulinic acid, 10 μM), T-5224 (10 μM) were applied with BLEO as small molecule inhibitors against NF-κB, C/EBP family and AP-1, respectively. SR (SR 11302, 3 μM), a positive control inhibitor against AP-1. Cells were lysed 7 days after treatment, with lysates subject to luciferase activity assay. **g** PSC27 cells were treated in the same conditions as described in **f**, with lysates collected for total RNA preparation and subject to quantitative RT-PCR analysis. Expression of EREG (left), IL6 (mid) or CXCL8 (right) was compared between CTLR (untreated), Mock (PBS-treated), BAY, BA, T-5224 and SR treatment groups. Cells were damaged by BLEO (50 μg/ml) or VBL (vinblastine, 20 nM) treatment. **h** Immunoblot analysis of DDR signaling (ATM), p38MAPK activation, cellular senescence (p16, p21) and NF-κB activation (p65) in PSC27 cells treated by various chemotherapeutic agents as indicated. GAPDH, loading control. **i** Immunoblot analysis Expression assay of p65 nuclear translocation in PSC27 cells treated by VBL, PTX, BLEO or MIT, individually. C cytoplasmic, N Nuclear. Histone H3, loading control for nuclear proteins. Note, the relative signal intensities (RSI, presented as percentage) of p65 were quantified as the virtual intensity of an individual sample after scanning, and calculated in relative to that of the strongest signal (BLEO, C for the p65 blot). **j** Presentation of p65-specific ChIP-seq tracks of the gene locus of several SASP hallmarks and senescence-associated factors. Illustrations were prepared from datasets deposited in the GEO (accession number GSE141992), with raw data available at publicly released sources [29]. Data are representative of three independent experiments. All *p* values were calculated by Student’s *t* tests. ^*p* > 0.05, *p < 0.05, **p < 0.01, ***p < 0.001.

Given the prominent induction of EREG in stromal cells after in vitro and in vivo genotoxic treatments, we reasoned the mechanism supporting EREG expression. As one of the key transcriptional machineries in mammalian cells, the NF-κB complex drives expression of multiple SASP factors upon replicative exhaustion-, oncogenic activation- or therapeutic agent-induced senescence (RS, OIS or TIS, respectively) [24]. We thus asked whether DNA damage-induced EREG expression is mediated by NF-κB signaling. Bioinformatics identified several putative NF-κB binding motifs in human *EREG* promoter region ~3700 bp upstream of the transcription starting site (Fig. 3a). Luciferase-based reporter assays substantiated the functional involvement of these NF-κB binding motifs using a group of *EREG* promoter constructs generated by sequential cloning. Compared to control HEK293 or PSC27 cells, both tumor necrosis factor α (TNF-α), a potent NF-κB agonist, and the genotoxic drug BLEO significantly elevated EREG reporter activity (Fig. 3b, c). The data were consolidated by pharmacological treatments with an NF-κB stimulator IL1α or the genotoxic drug, DOX, respectively (Supplementary Fig. 3a, b). Data from in vitro assays indicated that treatments by DDAs (DOX, MIT) tend to induce significant activation of NF-κB in the promoter region of EREG, generating signals considerably stronger than those induced by NDDAs (DTX, PTX and VBL) (Fig. 3c and Supplementary Fig. 3b, f). ChIP-PCR assays revealed that each of these binding sites (p2/p3/p4/p5) was indeed a bona fide motif physically bound by NF-κB upon genotoxic treatment (Fig. 3d). Functional involvement of NF-κB was further confirmed by treatment with Bay 11-7082 (BAY), an NF-κB antagonist that inhibits IκBα phosphorylation and blocks NF-κB activation. We found PSC27 cells pre-exposed to BAY exhibited markedly reduced EREG transcription, regardless of the genotoxic agents selected for in vitro expression assays (Fig. 3e).

Besides the NF-κB complex, other transcription factors such as C/EBP and AP-1 family members were also involved in SASP expression [25, 26], whereas their functional relevance in EREG induction remains unknown. To this end, we applied betulinic acid (BA), a pentacyclic triterpenoid that targets the C/EBP family [27], and T-5224, a selective inhibitor of AP-1 [28], to treat PSC27 cells pre-transduced with a reporter construct (pGL4-EREG-P05) encoding the approximal *EREG* promoter in frame of luciferase transgene. Genotoxic stress triggered a remarkable enhancement of signal intensity, which was abolished by the chemical BAY (*p* < 0.01 for both BLEO and DOX-damaged cells) (Fig. 3f and Supplementary Fig. 3g). Although treatment with BA resulted in a decline of reporter signals, the reduction fold was generally less than that caused by BAY (*p* < 0.05 for BA) (Fig. 3f). In contrast, influences generated by T-5224 and SR 11302 (SR), selective inhibitors of the AP family, were basically negligible (*p* > 0.05 for both). Transcript assays indicated that DNA damage-induced EREG upregulation was most effectively counteracted by NF-κB suppression, not C/EBP or AP-1 blockade (*p* < 0.01 for BAY, *p* < 0.05 for BA, *p* > 0.05 for T-5224 and SR) (Fig. 3g left). The case of EREG expression indeed largely resembled that of IL6 or CXCL8, two typical SASP hallmarks, induction of which seems to be mediated by NF-κB and C/EBP but independent of AP-1 (Fig. 3g middle and right). Overall, the data suggest that NF-κB plays a central role in mediating stromal EREG expression in genotoxic settings, although other transcription factors such as C/EBP are also functionally involved (Supplementary Fig. 3h).

Notably, we observed that the expression fold changes, including stress-associated induction and agent-caused inhibition of EREG in senescent cells were generally more dramatic in BLEO treatment settings (DDA) than in VBL exposure conditions (NDDA) (Fig. 3g). Immunoblot data suggested differential activation of DDR signaling and p38 pathway between NDDA and DDA cases, with the latter of a relatively higher capacity in inducing nuclear translocation of p65 (Fig. 3h, i), a pattern that is indeed consistent with the functional involvement of NF-κB in the SASP development, specifically in genotoxic backgrounds.

Recent studies with high-throughput sequencing disclosed alternations in chromatin openness (ATAC-seq), epigenetic modification (ChIP-seq) and transcription factors (such as p65) binding intensity of the promoter and enhancer regions of SASP factors [29–31]. We re-assessed the data by mapping the epigenetic regulatory profile of human EREG and noticed enhanced chromatin accessibility, augmented histone acetylation (specifically post-translational modification sites H3K18, H3K27, H3K122 and H4K5) and increased p65 (Rel A) association (enrichment) at the pre-existing NF-κB sites distributed across the promoter and distal enhancer of EREG in senescent fibroblasts relative to their normal controls (Supplementary Fig. 4). Of note, the characteristic tendency of EREG in these aspects largely resembled that of other SASP and/or senescence-associated factors, as exemplified by p65 binding at the cis-regulatory region of CXCL1, CXCL8, MMP1, PTGS2 and ICAM (Fig. 3j).

### Stromal EREG alters recipient cancer cell phenotypes

The expression of EREG is upregulated in multiple cancer types. Former studies reported EREG as an autocrine factor in promoting malignant phenotypes of PCa cells, and is one of the hub genes that mediate protein-protein interactions in the signaling network [32–34]. Here, we investigated the effect of paracrine EREG on PCa cell behaviors by culturing with stroma cell-derived conditioned media (CM). Upon treatment with the CM from PSC27 cells engineered to overexpress EREG (PSC27^EREG^) (Supplementary Fig. 5a), we observed markedly elevated proliferation of several PCa cell lines PC3, DU145, LNCaP and M12 (*p* < 0.01) (Supplementary Fig. 5b), accompanied by enhanced migration and invasion (Supplementary Fig. 5c, d). However, these gain-of-functions effects were almost completely depleted by EREG-specific shRNAs (Supplementary Fig. 5a), which retained normal proliferative potential of stromal cells but reversed the malignant phenotypes of recipient cancer cells (Supplementary Fig. 5b–d). Importantly, EREG enhanced the resistance of PCa cells to MIT, a DNA-targeting chemotherapeutic drug administered to cancer patients including those developing PCa [35, 36] (Supplementary Fig. 5e). MIT induced cleavage of caspase 3, a process remarkably weakened by stromal EREG but sustained upon elimination of EREG from PSC27 cells (Supplementary Fig. 5f), suggesting EREG drives cancer resistance largely via a caspase-counteracting mechanism. QVD-OPH and ZVAD-FMK, two potent pan-caspase inhibitors, as well as PAC1 and gambogic acid (GA), two caspase activators, were used to individually treat PC3 cells before exposure to MIT. We noticed substantially attenuated apoptotic activity upon application of QVD-OPH or ZVAD-FMK, even in the presence of EREG (Supplementary Fig. 5g). In contrast, apoptosis index was markedly elevated once the procaspase-activating compound PAC1 or GA was loaded, basically offsetting the anti-apoptosis effect of EREG. The effects were reproduced when docetaxel (DOC), another chemotherapeutic agent that disturbs microtubule depolymerization, was applied in culture (Supplementary Fig. 5h). However, EREG overexpression in stromal cells failed to confer survival advantage when these cells per se were treated with increasing concentrations of genotoxic chemicals such as BLEO, implying different survival mechanisms between cancer and stromal cells (Supplementary Fig. 5i). We further noticed a pronounced pattern of epithelial-to-mesenchymal transition (EMT) in PCa cells upon exposure to the EREG-containing CM derived from PSC27 cells, indicating the potential of EREG in propelling such a phenotypic switch (Supplementary Fig. 5j).

**Fig. 4:**
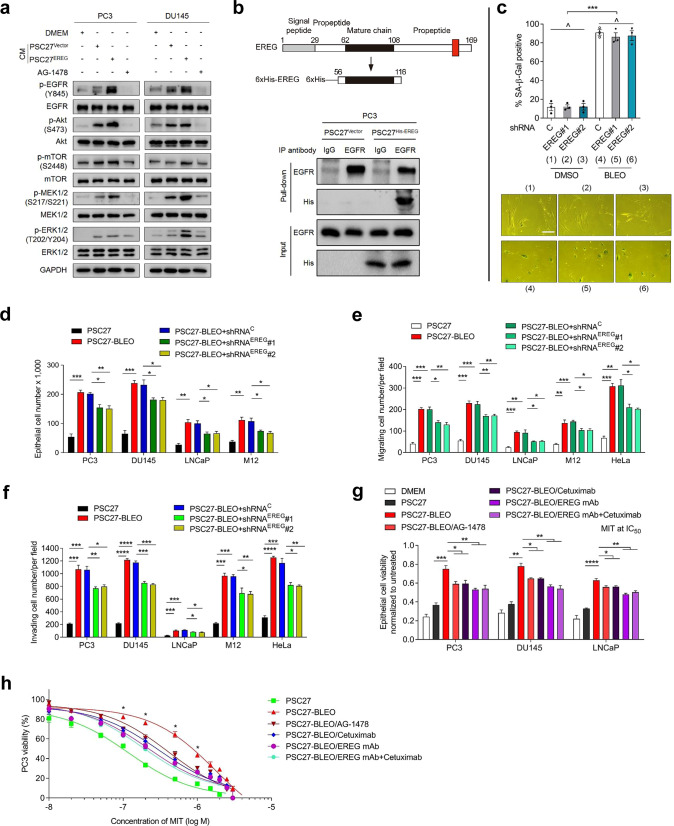
Stromal EREG alters multiple phenotypes of prostate cancer cells in vitro. **a** Immunoblot analysis of EGFR-associated pathways in PC3 and DU145 cells treated by the CM from PSC27 cells transduced with the empty vector or EREG construct, or alongside the EGFR inhibitor AG-1478 (2 μM). Antibodies of p-EGFR (Y845), p-Akt (S473), p-mTOR (S2448), p-MEK (S217/S221) and p-ERK (T202/Y204) were applied to probe the individual molecules. Total protein per molecule and GAPDH were used as loading control. **b** Schematic diagram of the construct encoding the mature chain of EREG (upper) and immunoprecipitation (IP, lower) followed by immunoblot assay of EGFR and His-EREG (fusion protein) in the whole lysates of PC3 cells. PC3 was treated by the CM of PSC27^Vector^ and PSC27^His-EREG^ for 3 days. Antibodies including IgG and anti-EGFR were used for IP, with both EGFR and His-EREG in inputs analyzed. **c** Measurement of cellular senescence by quantification of SA-β-Gal staining positivity. Stromal cells were pre-transduced with shRNAs and treated by BLEO. Upper, statistics. Lower, representative images. Scale bar, 20 μm. **d** PCa cells were treated with the CM from PSC27 sublines for 3 days, and subject to cell proliferation assay. Native and shRNA-transduced PSC27 cells as indicated were treated by bleomycin (BLEO), with the conditioned media (CM) collected 7 days after drug treatment and used for PCa cell culture. The CM were collected from equal number of cells per condition, with a starting DMEM that contains 0.5% FBS to make the CM. **e** Migration assay of PCa cells seeded within transwells in 6-well plates, with cells cultured for 3 days in the CM from PSC27 sublines depicted in **d**. **f** Invasiveness appraisal of PCa cells across the transwell membrane upon culture with the CM from PSC27 sublines described in **d**. **g** Chemoresistance assay of PCa cells cultured with the CM from PSC27 sublines described in **d**. MIT (mitoxantrone) was applied at the concentration of IC50 value pre-determined per cell line. AG-1478 (2 μM), cetuximab (50 μg/ml) or EREG mAb (1 μg/ml) were applied alongside with PSC27 CM. **h** Dose-response curves (non-linear regression/curve fit) plotted from drug-based survival assays of PC3 cells cultured with the CM of PSC27 native or damaged by bleomycin (PSC27-BLEO), and concurrently treated by a wide range of concentrations MIT. AG-1478, cetuximab or EREG mAb (1 μg/ml) were applied with PSC27 CM. Data are representative of three independent experiments. All *p* values were calculated by Student’s *t* tests. ^*p* > 0.05, *p < 0.05, **p < 0.01, ***p < 0.001, ****p < 0.0001.

Given the remarkable effects EREG caused in these in vitro assays, it is reasonable to specifically query the mechanism(s) supporting EREG to confer a pro-survival advantage on cancer cells. As EREG shares considerable sequence homology with other EGF family members [18], we chose to evaluate the capacity of EREG as an EGF-like growth factor. PSC27^EREG^ CM induced phosphorylation of EGFR (Y845), Akt (S473) and mTOR (S2448), indicating activation of the PI3K/Akt/mTOR pathway, while phosphorylation of MEK1/2 (S217/S221) and ERK (T202/Y204) suggested synchronous activation of MAPK signaling (Fig. 4a). However, upon addition of AG-1478 (Tyrphostin AG-1478, NSC 693255), a membrane receptor tyrosine kinase (RTK) inhibitor that selectively targets EGFR [37], EREG-induced EGFR activation was blocked, so was the engagement of both Akt/mTOR and MEK/ERK axes (Fig. 4a). Therefore, EREG-triggered activation of these two signaling pathways was indeed mediated by EGFR, although functional involvement of other RTKs cannot be arbitrarily excluded. As the antibody used in this study specifically recognizes the C-terminal of EREG, a fragment that is cleaved off upon maturation of EREG and cannot be used to probe ligand-receptor interactions, we chose to clone the mature chain of EREG propeptide for further studies. Consequently, we noticed a strong interaction between EREG and EGFR, as evidenced by the clear signal in pull-down precipitates generated by anti-His-EREG in immunoprecipitation assays (Fig. 4b).

We next interrogated whether EREG, a soluble factor in the full SASP spectrum of stromal cells, plays a major role in shaping advanced malignancies of cancer cells. Although EREG elimination from PSC27 neither delayed nor accelerated cellular senescence (Fig. 4c), exposure to the CM of BLEO-treated PSC27 (PSC27-BLEO) enhanced proliferation, migration and invasiveness of PCa cells (Fig. 4d–f). Clearance of EREG from stromal cells markedly diminished these malignancy-promoting potentials of PSC27-BLEO CM, with a reduction of 20–30% in the conducted assays (Fig. 4d–f).

Some SASP components including WNT16B and SFRP2 display strong capacity in conferring resistance to cancer cells [1, 38]. However, whether EREG plays a similar role in drug-damaged TME remains underexplored. We found the viability of cancer cells substantially increased upon exposure to damaged stromal cell-derived CM, although counteracted by ~30% upon EREG knockdown or AG-1478 treatment (Supplementary Fig. 5e and Fig. 4g). When the EREG-specific monoclonal antibody (EREG mAb) was used, a markedly reduced viability of PCa cells was observed, with the effect comparable to or even higher than that of either AG-1478 or cetuximab, the latter a Food and Drug Administration (FDA)-approved EGFR-targeting monoclonal antibody (Fig. 4g). Co-application of EREG mAb and cetuximab to culture achieved an effect largely reproducing that of EREG mAb alone (Fig. 4g), suggesting addition of cetuximab to EREG mAb did not provide an extra benefit. Although PSC27-BLEO CM enhanced viability of PC3 exposed to MIT at 0.1–1.0 μM, a range of dose approaching its serum concentrations in clinical settings [39, 40], EREG-neutralization markedly offset cancer resistance in a similar way as EREG mAb was combined with cetuximab (Fig. 4h). Hence, targeting either EGFR or EREG can significantly deprive cancer cells of stroma-conferred resistance to chemotherapeutic drugs.

### Paracrine EREG alters transcriptome-wide expression of cancer cells

**Fig. 5:**
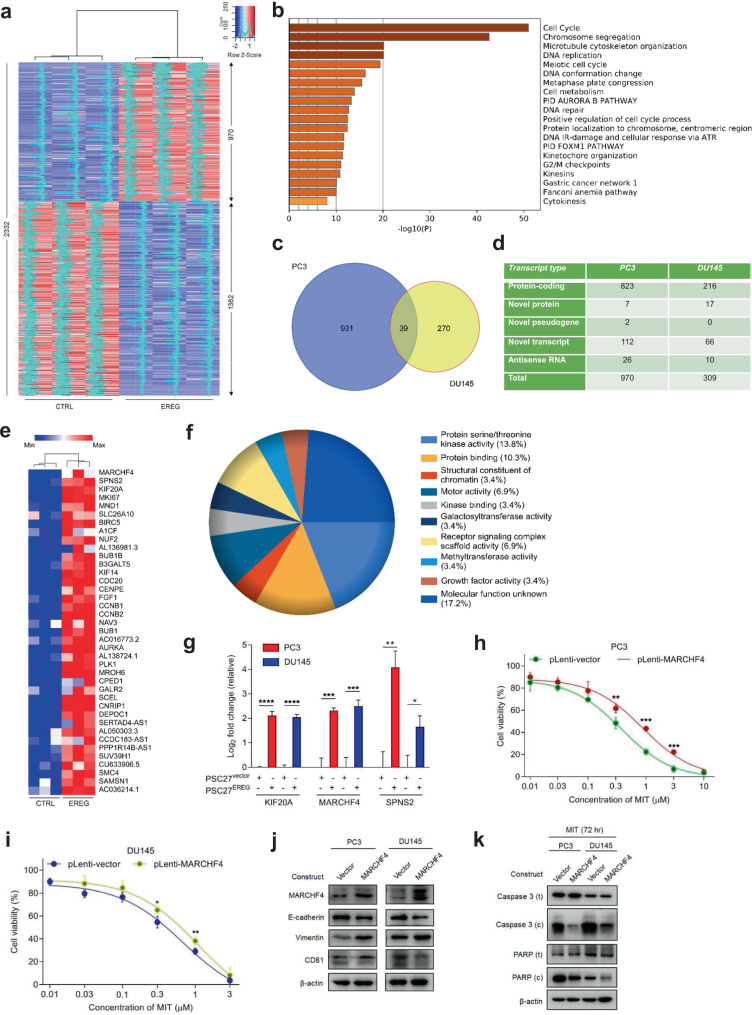
EREG induces profound changes of PCa cell expression profile and promotes phenotypic reprogramming. **a** Heatmap depicting differentially expressed human transcripts in PC3 cells after a 3-days culture with EREG-containing CM collected from PSC27 cells. In contrast to cancer cells cultured with control CM (CTRL), 970 and 1362 genes were upregulated and downregulated, respectively, in those treated with the CM from EREG-expressing PSC27 cells (EREG). **b** Graphical visualization of pathways by GO profiling. Those significantly enriched genes in the upregulated list were sorted according to their fold change in PC3 cells exposed to the CM of EREG-expressing PSC27 cells. **c** Venn diagram displaying the overlap of 39 transcripts upregulated in PC3 and DU145 cells upon treatment with EREG-containing CM from stromal cells (970 and 309 genes with unique annotations for PC3 and DU145, respectively). **d** Statistics of transcripts differentially expressed (fold change either ≥2 or ≤0.5, with *p* < 0.05) in PC3 and DU145 upon EREG stimulation, and classified into typical categories according to functional annotations mapped by Genecode (V27). **e** Heatmap showing the top 39 transcripts upregulated by both PC3 and DU145 cells, sorted according to their expression fold change in PC3. **f** Pie chart depicting the biological processes associated with transcripts upregulated by EREG after GO analysis of the 39 transcripts in PC3. **g** Quantitative RT-PCR measurement of the expression of KIF20A, MARCHF4 and SPNS2 in both PCa lines upon exposure to CM of stromal cells expressing EREG. Signals normalized to those of cells exposed to PSC27 cells transduced with vector. **h** Dose-response curves (non-linear regression/curve fit) plotted from drug-based survival assays of PC3 cells transduced with vector or MARCHF4 construct and treated by a range of concentrations of MIT. **i** Dose-response curves (non-linear regression/curve fit) plotted from drug-based survival assays of DU145 cells treated in a manner similar to that of PC3 cells. **j** Immunoblot assessment of protein expression of EMT-associated molecules. CD81, a downstream target of MARCHF4. β-actin, loading control. **k** Immunoblot profiling of apoptosis-related factors of self-cleavage activity in both PCa cell lines pre-transduced with vector or MARCHF4 construct and exposed to MIT for 72 h. β-actin, loading control. Data in **g**–**k** are representative of three independent experiments. All *p* values were calculated by Student’s *t* tests. ^*p* > 0.05, *p < 0.05, **p < 0.01, ***p < 0.001.

Given the remarkable modification of cancer cell phenotypes caused by stromal cell-derived EREG, we pursued to determine the influence of paracrine EREG on cancer cell expression. As established, EGFR can be activated by seven growth factors that fall into two groups based on receptor-binding affinity, including the group of high-affinity ligands including EGF, transforming growth factor-a, betacellulin, and heparin binding EGF-like growth factor and the group of low-affinity ligands including EREG, epigen (EPGN) and amphiregulin (AREG) [19]. Although former studies reported distinct EGFR-dependent cellular responses to various ligands [41], the paracrine influence of stromal EREG on global expression profiles of cancer cell subpopulations, more specifically, PCa cells, remain underexplored. In our study, we chose to first examine the impact of PSC27-derived EREG on cultured PCa lines. Whole transcriptome RNA sequencing and bioinformatics revealed that 2332 transcripts were significantly upregulated or downregulated (two-fold, *p* < 0.05) in PC3 cells by stromal EREG, with 1659 transcripts changed in DU145 cells (Fig. 5a and Supplementary Fig. 6a). We noticed remarkable changes in the biological processes of both PC3 and DU145, as evidenced by enhanced activities in cell cycle, chromosome segregation, microtubule organization, DNA replication, cell division and metabolism (Fig. 5b and Supplementary Fig. 6b). While the vast majority of these EREG-altered transcripts were protein-coding (1805 and 1123 for PC3 and DU145, respectively), there were also molecules that fall into the sub-categories of novel proteins, novel pseudogenes, novel transcripts, antisense RNAs, long intergenic non-protein-coding RNAs (Supplementary Fig. 6c, d).

Specifically, there were 970 and 309 transcripts significantly upregulated in PC3 and DU145 cells, respectively, with 39 identities commonly shared by both lines (Fig. 5c, d). After mapping the transcripts to a gene ontology database comprising HPRD, Entrez Gene and UniProt accession identifiers [42–44], we noticed these 39 genes mostly encode proteins of molecular functions associated with cancer progression, such as kinase activity, protein binding, motor activity and receptor signaling activity (Fig. 5e, f). Thus, our data suggest a salient capacity of paracrine EREG in reprogramming the transcriptome profile of recipient cancer cells with the potential to enhance their malignancy.

Further analysis confirmed the expression changes of PCa cells, such as upregulation of MARCHF4, SPNS2 and KIF20A in PC3 line (Fig. 5g). Among these top genes, we noticed MARCHF4, which is a member of the MARCH family of membrane-bound E3 ubiquitin ligases. MARCH enzymes can add ubiquitin to target lysines in substrate proteins, thereby signaling their vesicular transport between membrane compartments [45]. However, whether they are involved in development of cancer cell malignancy, specifically drug resistance, remains largely unknown. Thus, we chose to clone MARCHF4 and overexpress it in representative PCa lines. Experimental data suggested that MARCHF4 significantly enhanced the viability of both PC3 and DU145 cells in the condition of MIT treatment (Fig. 5h, i). Immunoblot data indicated a typical pattern of EMT upon ectopic expression of MARCHF4 in these cells, despite the lack of transcriptional alterations of several malignancy-related factors including those indicative of EMT (Fig. 5j and Supplementary Fig. 6e–g). Importantly, the apoptotic activity of PCa cells, which arose in response to MIT-delivered genotoxicity, was pronounced reduced when MARCHF4 was present (Fig. 5k). These data essentially substantiated the functional contribution of MARCHF4 to resistance of cancer cells to chemotherapeutic stress.

### Targeting EREG improves chemotherapeutic outcome in preclinical trials

Given the effects of EREG on the biological phenotype and expression profile of cancer cells in vitro, we next queried the pathological consequences that EREG can generate under in vivo conditions. To this end, we built tissue recombinants by admixing PSC27 sublines with PC3 cells at a pre-optimized ratio of 1:4 before subcutaneous implantation to the hind flank of experimental mice with severe combined immunodeficiency. Animals were measured for tumor size at the end of an 8-week period. Compared with tumors comprising PC3 and PSC27^Vector^, xenografts composed of PC3 and PSC27^EREG^ displayed significantly increased sizes (Supplementary Fig. 7a). Conversely, EREG knockdown from PSC27^EREG^ cells prior to tumor implantation considerably decreased tumor volumes.

**Fig. 6:**
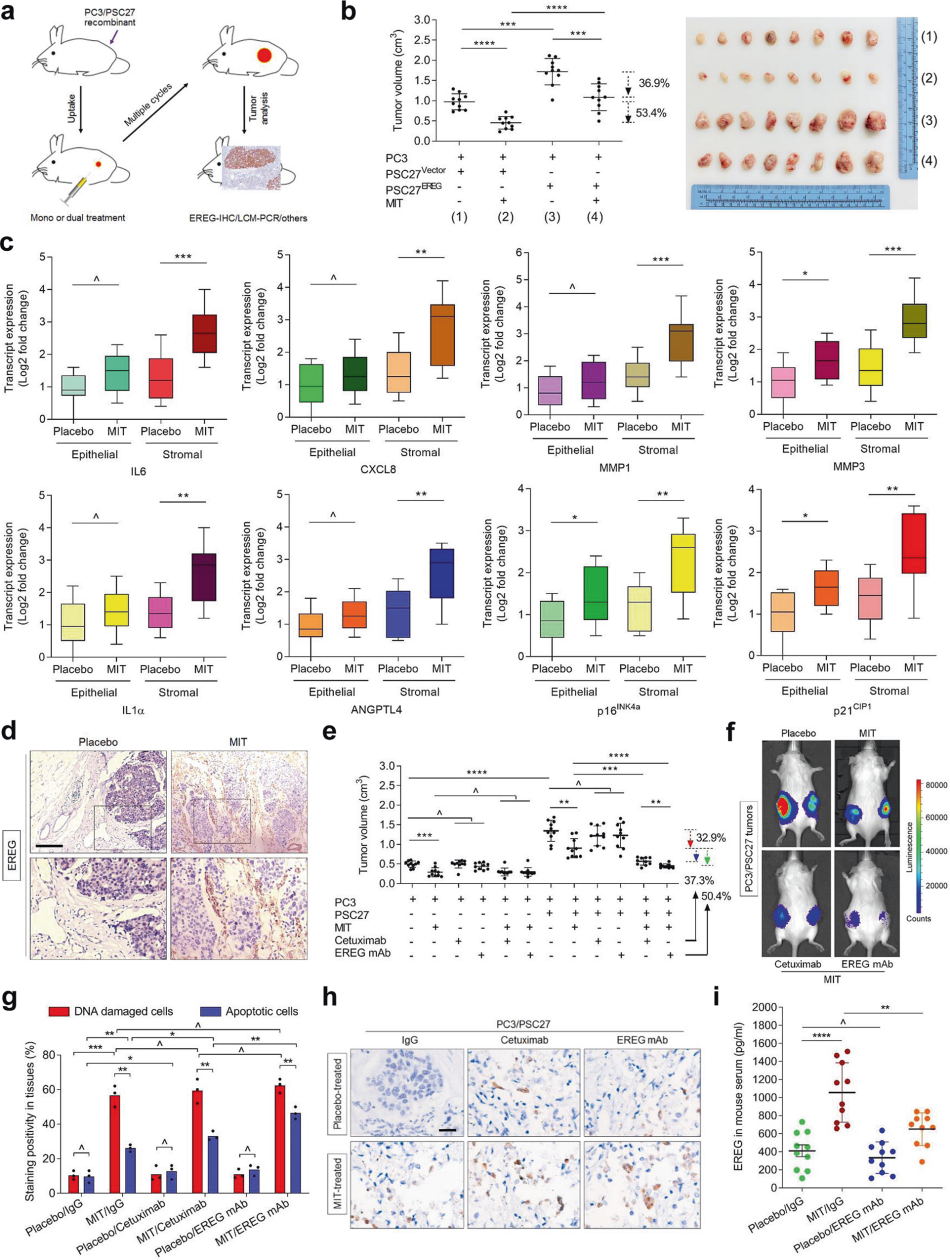
Therapeutically targeting EREG in the damaged TME promotes therapeutic outcome in preclinical trials. **a** Schematic workflow of experimental procedure for severe combined immunodeficient (SCID) mice. Two weeks after subcutaneous implantation and in vivo uptake of tissue recombinants, animals received either single or combinational agents administered as metronomic treatments composed of several cycles. **b** Statistical profiling of tumor end volumes. PC3 cells were xenograted alone or together with PSC27 cells to the hind flank of SCID mice. Prior to implantation, PSC27 cells were transduced with the control vector or EREG construct to make stable sublines. MIT was administered to induce tumor regression. Right, representative tumor images. **c** Transcript assessment of several canonical SASP factors expressed in stromal cells isolated from the tumors of SCID mice. Tissues from animals implanted with both stromal and cancer cells were subject to LCM isolation, total RNA preparation and expression assays. **d** Representative IHC images of EREG expression in tissues isolated from placebo or MIT-treated animals. Square regions in the upper images were zoomed into lower images. Scale bars, 100 μm. **e** Statistical comparison of tumor growth in animals that underwent several different treatment modalities. Mice were implanted with PC3 alone or in combination with PSC27, before treated by the chemotherapeutic drug (MIT) or combinational agents (MIT/cetuximab or MIT/EREG mAb). Tumor volumes were measured at the end of an 8-week preclinical regimen. **f** Representative bioluminescence images (BLI) of PC3/PSC27 tumor-bearing animals in the preclinical trial. Digital signals were proportional to in vivo luciferase activities measured by an IVIS device. **g** Statistical assessment of DNA-damaged and apoptotic cells in the tumor specimens analyzed in **e**. Values are presented as percentage of cells positively stained by IHC with antibodies against γH2AX/p-53BP1 (co-staining) or caspase 3 (cleaved). **h** Representative IHC images of caspase 3 (cleaved) in tumors at the end of therapeutic regimens. Biopsies of placebo-treated animals served as negative controls for MIT-treated mice. Scale bars, 50 μm. **i** EREG concentration assessment in circulating blood of experimental mice treated by chemotherapy and/or EREG mAb. Data were derived from human EREG-specific ELISA assays. Data are representative of three independent experiments. Animal studies were performed with ten mice per group (*n* = 10). All *p* values were calculated by Student’s *t* tests. ^*p* > 0.05, *p < 0.05, **p < 0.01, ***p < 0.001, ****p < 0.0001.

To closely mimick clinical conditions involving chemotherapeutic agents, we designed a preclinical regimen which incorporates genotoxic drugs and/or EREG/EGFR inhibitors (Fig. 6a and Supplementary Fig. 7b). Two weeks post cell implantation when stable uptake of tumors by host animals occurred, a single dose of MIT or placebo was administered at the 1st day of 3rd, 5th and 7th week until the end of the 8-week regimen. Contrasting to placebo, MIT treatment resulted in markedly reduced tumor sizes regardless of EREG expression in PSC27 cells, thus validating the efficacy of MIT as a cytotoxic agent (Fig. 6b and Supplementary Fig. 7c). We noticed a significant upregulation of SASP factors including IL6, CXCL8, IL1α, ANGPTL4, SPINK1, WNT16B, SFRP2 and MMPs, alongside induction of typical senescence markers including p16^INK4a^, p21^CIP1^ and SA-β-Gal in xenografts composed of PC3/PSC27^Vector^ cells, implying development of an in vivo senescence and the SASP in response to MIT treatment (Fig. 6c and Supplementary Fig. 7d, e). Data from IF assays indicated that stromal cells residing in xenografts grown subcutaneously were exclusively from implanted human PSC27 cells, rather than mouse (host) stromal cells migrating to the tumor foci (Supplementary Fig. 7f).

It is noticeable that expression of some SASP factors such as MMP3 and MMP12, together with the canonical senescence markers including p16^INK4A^ and p21^CIP1^, was induced by MIT in both stromal and cancer cells, suggesting chemotherapy caused comprehensive in vivo senescence, although the SASP profile seemed to be differently developed between these two cell populations (Fig. 6c and Supplementary Fig. 7e). However, EREG was more preferentially induced in xenografts of mice exposed to MIT treatment, with signals mainly arising from stromal cells (Fig. 6d).

We next interrogated whether technically depleting EREG from the full SASP spectrum of treatment-damaged stoma could further enhance the therapeutic response of tumors. To address this, we administered either cetuximab or EREG mAb with MIT since the first dose of preclinical treatment. Though MIT per se caused shrinkage of PC3-only tumors (*p* < 0.001), delivery of therapeutic antibodies did not show significant effect (*p* > 0.05) (Fig. 6e). Of note, these antibodies did not confer further benefits even when they were combined with MIT (*p* > 0.05), implying basically independence of PC3 tumor growth on the EGF/EGFR axis, specifically in the absence of stromal cells. Strikingly, upon combination of PC3 cells together with their stromal counterparts, we observed markedly increased tumor volumes (*p* < 0.0001), substantiating the tumor-promotive effect of stromal cells in vivo (Fig. 6e). However, when animals harboring PC3/PSC27 tumors were exposed to MIT, tumor volumes remarkably decreased (32.9%, *p* < 0.01). Upon co-administration of either cetuximab or EREG mAb with MIT as dual treatments, tumors displayed further shrinkage (37.3%, *p* < 0.001 and 50.4%, *p* < 0.0001, respectively) (Fig. 6e).

Chemotherapy can exert paradoxical effects as a double-edged sword, and therapeutic efficacy on primary tumors may be counterbalanced by tumor/host reactive responses enabling dissemination of cancer cell subpopulations, including those of potential for metastatic colonization [46]. Bioluminescence imaging of xenografts generated with cancer cells stably expressing luciferase (PC3-luc) and their stromal counterparts excluded the likely metastasis of cancer cells from primary sites, with bioluminescence signals essentially consistent with tumor growth patterns in individual animal groups (Fig. 6f). The data suggest that classic chemotherapy combined with TME-targeting agents can induce tumor responses more effectively than chemotherapy alone, with the efficacy of an EREG mAb even superior to that of cetuximab, an anti-EGFR agent widely used to restrain EGFR^+^ neoplastic cell expansion by inducing apoptosis in diverse malignancies [47].

To disclose the mechanism(s) inherently responsible for EREG-induced cancer resistance, we chose to dissect tumors from animals 7 days after initiation of treatment, a timepoint prior to resistant colony development. In contrast to placebo, MIT per se caused significant DNA damage and apoptosis in cancer cells (Fig. 6g). Cetuximab alone caused neither a typical DDR nor enhanced cell death in PC3/PSC27 xenografts, suggesting limited responses of these tumors when animals were exposed to cetuximab (Fig. 6g). Upon combination with MIT, cetuximab further enhanced cell apoptosis, implying a synergetic cytotoxicity when administered together with MIT. Contrasting cetuximab, however, EREG mAb generated even more apoptotic cells in tumor foci, achieving a significantly higher apoptotic index than this FDA-approved antibody (Fig. 6g) (*p* < 0.05). The pattern of in vivo apoptosis was largely consistent with that of tumor regression upon treatment by different agents. IHC staining disclosed enhanced caspase 3 cleavage, a typical cell apoptosis indicator, when EREG mAb was administered (Fig. 6h). ELISA data suggested that MIT-mediated chemotherapy resulted in elevated levels of circulating EREG in animals, a pattern that was largely reversed in the case of EREG mAb administration (Fig. 6i).

To expand, we employed LNCaP, a second PCa cell line which expresses androgen receptor (AR) and is routinely employed as a hormone-dependent cell model. To produce an AR-naïve setting, we avoided experimental castration, but followed the same protocol designed for PC3-tumor cohorts. We observed markedly reduced volumes of LNCaP/PSC27 tumors when mice underwent chemotherapy combined with antibodies (Supplementary Fig. 7g). These data evidently suggest that specific elimination of EREG from the whole spectrum of SASP in a treatment-damaged TME enhances tumor response to chemotherapy, a process independent of androgen regulation or AR signaling of prostate tumors per se.

Given the pronounced efficacy of combinational treatment in cancer therapy, we further expanded the study to breast tumors by generating xenografts comprising MDA-MB-231 (malignant) and HBF1203 (stromal) cells, a combination we previously employed for cancer research [48]. Again, MDA-MB-231/HBF1203 tumors largely reproduced the results of PCa preclinical experiments (Supplementary Fig. 7h). Our findings suggest that the resistance-minimizing effects of EREG-targeting strategy are not limited to a specific cancer type, but likely applicable to a wide range of malignancies.

To establish the safety and feasibility of above therapeutic regimens, we performed routine pathophysiological appraisal. The data supported that either single or combinational treatment was well tolerated, as evidenced by body weight maintenance throughout the therapeutic timeframe (Supplementary Fig. 8a). No significant perturbations in serum levels of creatinine, urea and metabolic activities of liver enzymes (ALP and ALT) were observed (Supplementary Fig. 8b). Additional data from animals developing breast tumors and treated by DOX/antibody or MIT/antibody-treated immunocompetent animals (in a C57BL/6J background) largely phenocopied PCa mice by exhibiting no routine blood count changes, thus further validating the findings (Supplementary Fig. 8c–g). Together, these results suggest that combining an EREG-targeting agent with conventional chemotherapy holds the potential to enhance tumor response without causing severe cytotoxicity.

### EREG is an emerging biomarker indicative of the SASP in cancer medicine

**Fig. 7:**
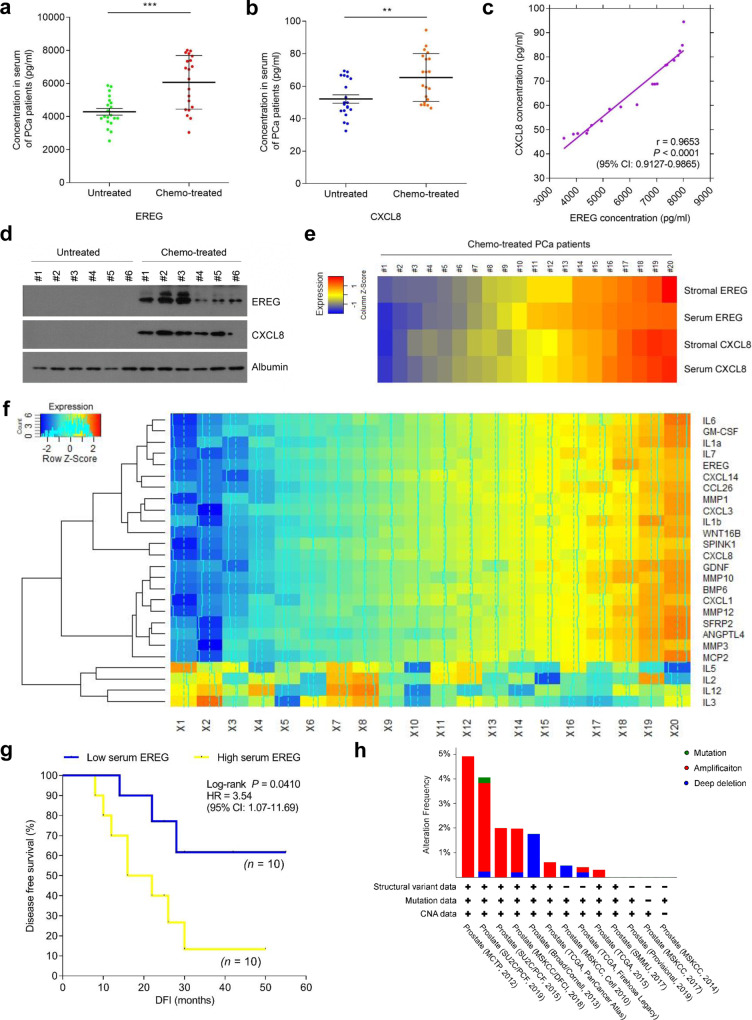
EREG is a novel circulating biomarker indicative of the SASP in vivo and predicts adverse therapeutic outcome in cancer clinics. **a** Abundance of EREG protein in the serum of untreated and chemo (MIT)-treated PCa patients. Data were derived from ELISA measurement and shown as mean ± SD. *N* = 20. **b** Abundance of CXCL8 protein in patient serum analyzed in **a**. Data from ELISA assays and presented as mean ± SD. *N* = 20. **c** Scatterplot showing correlation between EREG and CXCL8 in the serum of individual patients studied in **a** and **b**. Pearson’s correlation coefficient, *p* value and confidence interval are indicated. **d** Immunoblot examination of EREG and CXCL8 circulating in the serum of randomly selected PCa patients from untreated and chemo (MIT)-treated groups, respectively (*n* = 6 per group). Albumin, loading control for patient serum protein. **e** Heatmap depicting the overall correlation between stromal EREG, serum EREG, stromal CXCL8 and serum CXCL8 in chemo (MIT)-treated patients (*n* = 10). The raw scores of stromal EREG and CXCL8 were derived from independent pathological reading of primary tumor tissues of PCa patients, with those of serum EREG and CXCL8 obtained from ELISA assays. Color key, relative expression of these two factors in stromal tissue or patient serum. **f** Heatmap showing the relative expression of a panel of SASP signature factors in the tumor stroma of PCa patients, and the correlation of EREG/CXCL8 with these factors (*n* = 20). Stromal cells in the tumor tissues were isolated via LCM and expression of each target factor was measured by qRT-PCR, with signals per factor group normalized to that of the sample showing the lowest expression value. A subset of inflammatory factors typically not considered as SASP components was examined as random control (IL-2/3/5/12). Correlations of these factors are presented as dendrogram organized by hierarchical clustering. Trace lines indicate the trends of signal main streams. **g** Kaplan–Meier survival analysis of chemo (MIT)-treated PCa patients. Disease-free survival (DFS) stratified according to EREG expression in tumor stroma (low, average score <2, blue line; high, average score ≥2, yellow line). DFS represents the length (months) of period calculated from the date of chemotherapy to the point of first time disease relapse. Survival curves generated according to the Kaplan–Meier method, with *p* value calculated using a log-rank (Mantel–Cox) test. *N* = 10 per group. **h** TCGA data show alterations of EREG in human prostate cancer patients at genomic level, including mutation, amplification and deep deletion. Alteration frequency is displayed in percentage. Data in **a**–**c** are representative of three independent experiments. **p < 0.01, ***p < 0.001. *p* values were calculated by Student’s *t* test (**a**, **b**), Pearson test (**c**) and log-rank (Mantel–Cox) test (**g**). ^*p* > 0.05, **p < 0.01, ***p < 0.001.

Although higher EREG expression in the tumor foci is correlated with lower survival rate of posttreatment cancer patients (Fig. 2I and Supplementary Fig. 2I), whether blood-borne EREG is technically detectable and can be used as a marker for clinical prediction remains unclear. To address this, we acquired peripheral blood samples from PCa patients, including one cohort that experienced standard chemotherapy and the other that did not. ELISA assays of the serum from chemo-treated patients revealed EREG levels in the treated cohort significantly higher than that of the treatment-naïve group (Fig. 7a). The pattern was essentially reproduced by a remarkable increase of CXCL8, a canonical hallmark of the SASP, in the same cohort of post-treatment patients (Fig. 7b). The data suggest development of an in vivo SASP, the index of which can be measured by quantifying concurrently expressed soluble factors, including but may be not limited to EREG and CXCL8, in the peripheral flood of post-treatment cancer patients.

It is intriguing to determine whether the blood levels of EREG are correlated with those of other typical SASP factors such as CXCL8 in a same individual patient after clinical treatment. Data from ELISA tests disclosed a significant and positive correlation between EREG and CXCL8 (Fig. 7c). Subsequent immunoblots not only confirmed elevated levels of EREG and CXCL8 in the serum of chemo-treated patients, but indicated their simultaneous changes in vivo, thus establishing an intimate correlation between these two SASP factors in the serum of a same individual (Fig. 7d). An additional dataset was obtained from a BCa patient cohorts, which exhibited a pattern resembling that of the PCa cohort, thus providing an extra layer of clinical evidence (Supplementary Fig. 9a–c).

We then expanded the study by longitudinal analysis of these factors in both the primary tumor foci and peripheral blood (20 chemo-treated patients). Surprisingly, cross-organ comparisons indicated a pronounced association between in-tissue expression and circulating level per factor, with the amounts of EREG and CXCL8 apparently varying in parallel in either primary tissue or peripheral blood of each individual (Fig. 7e). To establish the accurateness and reliability of employing EREG/ CXCL8 for in vivo SASP appraisal, we selectively captured stromal cells from the primary tissues of PCa patients via LCM, and analyzed the levels of a subset of typical SASP factors, including but not limited to IL6, GM-CSF, IL1α, IL-1β, IL7, WNT16B, SPINK1 and MMPs (Fig. 7f). Signal intensity of the vast majority of these factors in stromal cells consistently paralleled that of both EREG and CXCL8 in the same tissues. However, albeit not surprisingly, representative non-SASP factors such as IL2/3/5/12 failed to show inherent correlations with EREG and CXCL8 (Fig. 7f). Together, our data suggests that EREG indeed represents one of the critical TME-derived soluble factors precisely imaging the development of an in vivo SASP, and can be exploited to assess the SASP magnitude in cancer patients.

Clinical profiling further revealed a negative correlation between plasma level of EREG and post-treatment survival of PCa patients, further substantiating the pathological impact of EREG as a TME-derived SASP factor, which directly predicts adverse outcome once the TME is subject to irreparable damage by clinical interventions (Fig. 7g). Data from the BCa cohort largely confirmed the intimate association between tissue expression and circulating level of EREG/CXCL8, and clinical relevance of plasma EREG in patient survival (Supplementary Fig. 9d–e). As EREG is subject to frequent mutation, amplification and deep deletion as suggested by the TCGA pan-cancer atlas studies which document global genomics data (Fig. 7h and Supplementary Fig. 9f), this factor has been considered an important predictor of disease progression in treatment-naïve patients of multiple cancer types [49–51]. In this study, we propose that beyond the hitherto well-known diagnostic modalities, routine surveillance of EREG in post-treatment cancer populations through a noninvasive approach such as liquid biopsy, can provide a novel, handy and practical strategy for both prognosis and prevention of advanced pathologies in clinical oncology.

## Discussion

Numerous efforts have been devoted to clarify the resistance mechanisms inside cancer cells per se, such as reduced drug accumulation, increased detoxification activity, enhanced DNA repair and disabled apoptotic machineries [52, 53]. However, advances in cell culture platforms, high-throughput techniques, animal models and analytic pipelines have demonstrated the pivotal roles of the TME in development of drug resistance, especially under clinical pressure. Instead of overly focusing on cell-autonomous or intrinsic mechanisms of cancer cells, we demonstrate the functional significance of the treatment-damaged TME in conferring acquired resistance to anticancer regimens, whereby a stroma-derived molecule EREG substantially contributes to cancer progression. Several lines of investigations established that stromal expression of soluble factors including HGF, TNF-α and WNT16B in the TME can promote cancer resistance to chemotherapy, radiation and targeted agents [1, 54–56]. In this study, we further substantiate the pathological influence of the TME on disease exacerbation by producing soluble factors, such as EREG (Supplementary Fig. 9g).

Repairable DNA damage usually does not cause comprehensive cytokine secretion, but severe genotoxic stress can trigger a persistent DDR and initiate development of the SASP, a hallmark feature of cellular senescence that forms after occurrence of inherent or environmental insults including anticancer therapeutics [6, 57]. The SASP is preferentially activated by stimuli that involve DNA damage, modulated by stress-response kinases including ATM/ATR, CHK2 and NBS1, and reciprocally consolidated by a few SASP factors such as the pro-inflammatory IL6 and CXCL8 [57–60]. Though intracellular molecules such as p38, mTOR, GATA4 and BRD4 regulate the SASP expression, they eventually engage activation of the NF-kB complex [61–63]. Here we show that genotoxicity-induced stromal cell expression of EREG involves not only NF-kB signaling, but also the C/EBP family. Although previous studies suggested the presence of C/EBP binding sites in the promoters of SASP factors particularly a subset of CXCR2 ligands including IL6, CXCL8, ENA-78 (CXCL5), GROα/β/γ (CXCL1/2/3), and NAP2 (CXCL7) [60], we extended the range of C/EBP-regulated SASP factors by adding EREG. Of note, a recent study reported that c-Myb and C/EBP co-regulate osteopontin (OPN) and many other SASP components [64], further indicating the regulatory complexity of SASP expression.

Although implications of EREG in cancer progression have been extensively investigated, only recently that mechanisms inherently correlated with its distinct functions were revealed. A study employing approaches including crystallography unraveled how EREG, a typical ligand for EGFR, stabilizes different dimeric conformations of the EGFR extracellular region [19]. Specifically, as a partial agonists of EGFR dimerization, EREG induces less stable EGFR dimers than other EGFR ligands such as EGF, while the weakened dimerization elicits more sustained EGFR signaling. In cancer clinics, upregulated EREG expression predicts a poor prognosis, but potentially benefits from therapies involving anti-EGFR agents such as panitumumab [65, 66]. Unlike other EGFR ligands, EREG mimics EGFR mutations by sustaining EGFR-ERK pathway activation, while high EREG expression sensitizes tumors to treatment by the EGFR inhibitor erlotinib [65]. EREG enhances glycolysis through activating EGFR signaling and its downstream glycolytic genes in tamoxifen-resistant BCa cells, whereby EREG is a direct target of miR-186-3p, downregulation of which by tamoxifen causes EREG upregulation in these cancer cells [67]. In this study, we found stromal cell-derived EREG not only activates Akt/mTOR, MEK/ERK pathways, signaling branches downstream of EGFR, but also generates a profound impact on genome-wide expression of cancer cells. First, experimental data suggested the emergence of EMT, a phenotypic switch as reflected by concurrent expression changes of EMT-specific markers. Second, we observed gene expression pattern indicative of regulations that may involve enhanced ubiquitination in cancer cells, a process mediated by upregulation of the E3 ubiquitin ligase MARCHF4. Although there is a limited number of literatures correlating MARCHF4 and cancer progression, our study suggest that cancer cell resistance driven by stromal cell-derived EREG, is at least partially mediated by MARCHF4 upregulated in recipient cancer cells. Although elimination of EREG from stromal cells generated a seemingly limited reduction, generally 20–30% as compared to the amplitude caused by the full SASP spectrum, of the malignant phenotypes of recipient cells, the changes were found statistically significant. Considering the large number of SASP molecules released by senescent cells, EREG appears to be a potent SASP factor that deserves attention. Altogether, expression of key molecule(s) associated with cancer resistance suggests enhanced aggressiveness caused by paracrine EREG and indicates an adverse prognosis in the post-treatment stage.

Data from our preclinical studies support that EREG mAb holds the advantage by directly neutralizing EREG protein in the extracellular space, as the ligand does not have to undergo conformational change to allow maximal intermolecular interplay between antibody and the antigen as exemplified by the case of EGFR [19]. Elimination of EREG from stromal cells produced remarkable effects by restraining cancer cell malignancy, both substantiating EREG as one of the major factors across the SASP spectrum in shaping cancer plasticity, and suggesting the exploitable value of targeting EREG to minimize cancer resistance acquired from a treatment-damaged TME. Data from in vitro assays indicated that EREG-associated effects are mediated predominantly through EGFR, but involvement of other RTK cannot be arbitrarily excluded and yet remains possible, an issue deserving future exploration. Despite the known autocrine and paracrine effects of EREG on cancer cells, including enhanced proliferation, invasiveness, EMT switch and drug resistance, which were observed in multiple cancer types, our work provides a new avenue to understand the differential response of cell subpopulations in the TME, and supports that targeting both senescent stromal cells and cancer cells holds the potential to achieve maximal therapeutic outcome. In this study, we explored the possibility of controlling cancer resistance by targeting one of the major SASP factors, EREG, which acts as a critical player in driving acquired resistance, with EREG emerging as the SASP-related targetable molecule. Our work not only provides a rationale for development of humanized mAbs to EREG, but imply the technical feasibility of curtailing drug resistance by delivering a panel of humanized mAbs against the key SASP factors, to maximally improve therapeutic outcome in cancer clinics.

We found enhanced levels of EREG in circulating blood of both experimental animals and human patients post-chemotherapy. It is thus reasonable to appraise the potential of EREG as a potential biomarker indicative of a treatment-damaged TME in clinical settings. Nevertheless, one caveat to this study is the animal model used for preclinical assays. Although males were consistently chosen to make tumor xenografts at their hind flank, we have to admit that the TME of human prostate, human breast, murine prostate, and murine mammary are all different. Despite the complexity and variability of the microenvironment across organ and/or species types, however, we do speculate that EREG expression is not limited to the TME of a specific cancer type such as PCa or BCa, but may be universal across diverse malignancies, a feature that merits sufficient attention. Indeed, high EREG expression in the microenvironment is found to be correlated with advanced pathological stages, cancer cell invasion, distant metastasis, shorter OS and DFS of diverse cancer types including oral squamous cell carcinoma, gastric cancer, glioblastoma, colorectal and non-small cell lung cancer [11, 12, 68–71], supporting EREG one of the competent targets for anticancer therapies. Although there still remains much to do before the strategy involving EREG-specific targeting in an in vivo system can be technically translated to clinical settings, our study provides a new modality that may be further improved to minimize drug resistance by targeting the treatment-damaged TME of cancer patients. Furthermore, despite the technical advancement of well-validated assays of blood-borne soluble factors, comprehensive subtyping using EREG-based assays for pan-cancer investigation remains an unexplored but exciting and promising area in translational medicine.

## Materials and methods

### Cell culture

Primary normal human prostate stromal cell line PSC27, breast stromal cell line HBF1203 and lung stromal cell line HFL1 (ATCC) were maintained in stromal complete medium as described [1]. PCa epithelial cell lines PC3, DU145, LNCaP and lung cancer epithelial cell lines A549, NCI-H460 and NCI-H1299, BCa epithelial cell line MDA-MB-231 (ATCC) were routinely cultured with RPMI 1640 (10% FBS). PCa epithelial line M12 was a kind gift from Dr. Stephen Plymate, which derived from BPH1 but phenotypically neoplastic and metastatic [72]. All cell lines were routinely tested for mycoplasma contamination and authenticated with STR assays.

### Cell treatments

Stromal cells were grown until 80% confluent (CTRL) and treated with 50 μg/ml BLEO, 2 μM MIT, 5 μM DOX, 50 nM DTX, 50 nM PTX or 20 nM VBL. After treatment, the cells were rinsed briefly with PBS and allowed to stay for 7–10 days prior to performance of various examinations.

### Human cancer patient recruitment and biospecimen analysis

Administration of chemotherapeutic agents was performed for primary PCa patients (Clinical trial no. NCT03258320) and infiltrating ductal BCa patients (NCT02897700), by following the CONSORT 2010 Statement (updated guidelines for reporting parallel group randomized trials). Patients with a clinical stage ≥I subtype A (IA) (T1a, N0, M0) of primary cancer but without manifest distant metastasis were enrolled into the multicentered, randomized, double-blinded and controlled pilot studies. Age between 40–75 years with histologically proven PCa, or age ≥18 years with histologically proven infiltrating ductal BCa was required for recruitment into the clinical cohorts. Data regarding tumor size, histologic type, tumor penetration, lymph node metastasis, and TNM stage were obtained from the pathologic records. Tumors were processed as FFPE biospecimens and sectioned for histological assessment, with alternatively prepared OCT-frozen chunks processed via LCM for gene expression analysis. Specifically, stromal compartments associated with glands and adjacent to cancer epithelium were separately isolated from tumor biopsies before and after chemotherapy using an Arcturus (Veritas Microdissection) laser capture microscope following previously defined criteria [1]. The immunoreactive scoring (IRS) gives a range of 1–4 qualitative scores according to staining intensity per tissue sample. Categories for the IRS include 0–1 (negative), 1–2 (weak), 2–3 (moderate), 3–4 (strong) [73]. The diagnosis of PCa and BCa tissues was confirmed based on histological evaluation by independent pathologists. Randomized control trial protocols and all experimental procedures were approved by the Institutional Review Board of Shanghai Jiao Tong University School of Medicine, with methods carried out in accordance with the official guidelines. Informed consent was obtained from all subjects and the experiments conformed to the principles set out in the WMA Declaration of Helsinki and the Department of Health and Human Services Belmont Report.

### In vivo SASP assessment of patients and ELISA assays

Sections of clinical biospecimens or animal tissues were processed via LCM for gene expression analysis. Specifically, stromal compartments associated with glands in patient tumor samples were separately isolated using an Arcturus (Veritas Microdissection) laser capture microscope following the criteria defined formerly [1, 22]. For tumors grown from xenografts composed of human cells, OCT sections were first H&E-stained to determine the location of stromal cells and the stroma-epithelium border, with cell lineages then separately acquired by LCM. Transcript levels of human SASP canonical factors including IL6, CXCL8, WNT16B, SPINK1, IL1α, SFRP2, MMP1, MMP3 and MMP12 were measured by qRT-PCR (primers listed in Table S5).

Peripheral blood samples from cancer individuals with matched FFPE or frozen tumor samples were collected in EDTA tubes and centrifuged at 2000 × *g* for 10 min at room temperature within 1 h of clinical acquisition to prepare high quality serum. EREG and CXCL8 proteins in serum of cancer patients were subject to quantification by antigen-specific ELISA kits (R&D Systems, DY1195-05) according to manufacturer’s instructions. Detection limits for these factors were 5 pg/ml.

### Experimental animals and preclinical studies

See Supplementary Information.

### Statistics

See Supplementary Information.

## Supplementary information

Supplemental material

## Data Availability

The raw RNA-seq data have been deposited in the Gene Expression Omnibus database (accession code GSE173383). All sequencing experiments were performed as independent triplicates, and the RNA-seq data referenced during the study are available in a public repository (https://www.ncbi.nlm.nih.gov/geo/). For bioinformatics-based epigenomic profiling, publicly available data of proliferating and senescent cells were re-analyzed after acquisition from sources associated with previous studies (GSE141992 from Liu et al. [29] and GSE106146 from Sen et al. [30] for ChIP-seq, and GSE103588 from Parry et al. [31] for ATAC-seq, respectively). Further information and requests for resources and reagents should be directed to YS (sunyu@sibs.ac.cn).
