# Supplementary material for: Targeting epiregulin in the treatment-damaged tumor microenvironment restrains therapeutic resistance
Source: Oncogene. 2022 Oct 6;41(45):4941–59. doi: 10.1038/s41388-022-02476-7 (PMC9630100; doi:10.1038/s41388-022-02476-7)
Supplement: Supplementary file 1 — Supplemental material [file 41388_2022_2476_MOESM1_ESM.pdf]

# Supplementary Information online

Supplementary Materials and Methods

Supplementary Figures (1-9)

Supplementary Figure legends (1-9)

Supplementary Tables (1-6)

## Supplementary Materials and Methods

### Vectors, viruses and infection

Full length human EREG sequence was cloned into pLenti-CMV/To-Puro-DEST2 as described [1]. Small hairpin RNAs (shRNA) targeting sequences for EREG (1#, CGTCGGTTCCACATATTATTT; 2#, CCAACCTTTAAGCAAAGAGTA; 3#, CGTGTGGCTCAAGTGTCAATA) (scramble, CCTAAGGTTAAGTCGCCCTCG) were cloned in pLKO.1-Puro vector (Addgene). Upon production by 293T cells, lentivirual titers were adjusted to infect ~ 90% of cells. Stromal cells were infected overnight in the presence of polybrene (8 μg/ml), allowed to recover for 48 h and selected for 72 h before used for further analysis. For expression of target genes in either stromal or epithelial cells, total RNA was prepared and subject to qRT-PCR assays (primers listed in Table S5), immunoblot and immunofluorescence analysis.

### Histology and immunohistochemistry

Formalin-fixed paraffin-embedded (FFPE) tissue sections of 7 μm were deparaffinized in xylenes and rehydrated through a graded series of alcohols. Routine histology appraisal was performed with hematoxylin and eosin staining. For immunohistochemical (IHC) evaluation, FFPE sections experienced antigen retrieval with sodium citrate, incubation with 3% $H_2O_2$, treatment with avidin/biotin blocking buffer (Vector Laboratories) and then 3% BSA for 30 min. Staining with primary and secondary antibodies was conducted at 4°C for overnight and at room temperature for 60 min, respectively. Sections were incubated with a $H_2O_2$-diaminobenzidine (DAB) substrate kit (Vector, SK-4100). Samples were counterstained with hematoxylin, dehydrated and mounted. IHC images were obtained using an upright microscope (Olympus BX51). Brown staining indicated the immunoreactivity of samples.

**RNA-seq and bioinformatics analysis**

Total RNA samples were obtained from PC3 and DU145 cells cultured with CM of either $PSC27^{Vector}$ or $PSC27^{EREG}$ (collected from DMEM containing 0.5% FBS, with an equal number of cells *per* condition). Sample quality was validated by Bioanalyzer 2100 (Agilent), and RNA was subjected to sequencing by Illumina NovaSeq 6000 with gene expression levels quantified by the software package RSEM (https://deweylab.github.io/RSEM/). Briefly, rRNAs in the RNA samples were eliminated using the RiboMinus Eukaryote kit (Qiagen, Valencia, CA, USA), and strand-specific RNA-seq libraries were constructed using the TruSeq Stranded Total RNA preparation kits (Illumina, San Diego, CA, USA) according to the manufacturer's instructions before deep sequencing.

Pair-end transcriptomic reads were mapped to the reference genome (GRCh38/hg38) with reference annotation from Gencode v27 using the Bowtie tool. Duplicate reads were identified using the picard tools (1.98) script mark duplicates (https://github.com/broadinstitute/picard) and only non-duplicate reads were retained. Reference splice junctions are provided by a reference transcriptome (Ensembl build 73) [1]. FPKM values were calculated using Cufflinks, with differential gene expression called by the Cuffdiff maximum-likelihood estimate function [2]. Genes of significantly changed expression were defined by a false discovery rate (FDR)-corrected *P* value < 0.05. Only ensembl genes 73 of status "known" and biotype "coding" were used for downstream analysis.

Reads were trimmed using Trim Galore (v0.3.0) (http://www.bioinformatics.babraham.ac.uk/projects/trim_galore/) and quality assessed using FastQC (v0.10.0) (http://www.bioinformatics.bbsrc.ac.uk/projects/fastqc/). Differentially expressed genes were subsequently analyzed for enrichment of biological themes using the DAVID bioinformatics platform (https://david.ncifcrf.gov/), the Ingenuity Pathways Analysis program (http://www.ingenuity.com/index.html). Raw data of RNA-seq

were deposited in the NCBI Gene Expression Omnibus (GEO) database under the accession code GSE173383.

### *Venn diagrams*

Venn diagrams and associated empirical *P*-values were generated using the USeq (v7.1.2) tool IntersectLists [3]. The t-value used was 22,008, as the total number of genes of status “known” and biotype “coding” in ensembl genes 73. The number of iterations used was 1,000.

### *RNA-seq heatmaps*

For each gene, the FPKM value was calculated based on aligned reads, using Cufflinks [2]. Z-scores were generated from FPKMs. Hierarchical clustering was performed using the R package heatmap.2 and the distfun = “pearson” and hclustfun = “average”.

### EREG promoter characterization and ChIP-PCR assays

A 3700 bp genomic region upstream of human EREG ORF was analyzed for core NF-κB binding sites. The immediate 5’ upstream sequences containing putative NF-κB binding sites were amplified from the human genomic DNA. Four regions that encompass augmenting numbers of NF-κB binding sites were cloned into a luciferase reporter vector pGL4.22 (Promega). A NAT11-Luc2CP-IRES-nEGFP construct was used as a positive control as described [4]. After reporter constructs were co-transfected with a pRL-TK vector for transfection normalization, cells were treated with 40 ng/ml TNF-α (or 20 ng/ml IL-1α) for 3 hr, or 50 μg/ml bleomycin for 1 d. Luciferase activity was measured using the Dual-Luciferase Reporter Assay System (Promega). The NF-κB inhibitor Bay 11-7082 (5 μM), C/EBP antagonist betulinic acid (BA, 10 μM), c-Fos/AP-1 suppressor T-5224 (10 μM) and AP-1 inhibitor SR11302 (3 μM) were applied, with cells lysed 3 days later for luciferase assays.

For ChIP-PCR assays, four primer sets were designed to amplify short sequences within the approximal promoter region [primer set #2 (-482 to -259) forward 5'-CTACTGAAATCACAGTGAAGTATAG-3', reverse 5'-CTGTTCATTGCATCCTGCTAT-3'; primer set #3 (-1870 to -1625) forward 5'-GACCAGTCTGGCCAACATGG-3', reverse 5'-CCTCATGCTGTATGTTAGATATTCAGAC-3'; primer set #4 (-1917 to -1773) forward 5'- TACTTTGGGAGGCCGAGGCAG-3', reverse 5'-CTCCCGAGTAGCTGGGATTACAGG-3']; primer set #5 (-4000 to -3798) forward 5'- TTTAAGAACCTACTATGTGTTTGG-3', reverse 5'-GAAACTCTTGGACACTTTGAG-3']. Additionally, two specific primer sets were employed to amplify regions within the promoters of the human IL6 (forward 5'-AAATGCCCAACAGAGGTCA-3', reverse 5'-CACGGCTCTAGGCTCTGAAT-3') and CXCL8 (forward 5'- ACAGTTGAAAACTATAGGAGCTACATT-3', reverse 5'-TCGCTTCTGGGCAAGTACA-3') genes, respectively, which encompass known NF-κB binding sites [4]. ChIP assays were then performed on PSC27 cells of early passage (p10) and those treated by 50 μg/ml bleomycin in culture. A sample of formalin-fixed sheared chromatin (DNA fragments at an average size of ~500 bp) from these cells was used as “input DNA” for control amplification. Fixed chromatin was immunoprecipitated using mouse monoclonal anti-p65 antibody (Santa Cruz) and DNAs were extracted from the immunoprecipitates and amplified using the primer sets described above. Control immunoprecipitations were carried out with a mouse IgG, which essentially yielded no reaction products.

**Immunoblot and immunofluorescence analysis**

Whole cell lysates were prepared using RIPA lysis buffer supplemented with protease/phosphatase inhibitor cocktail (Biomake). Nitrocellulose membranes were incubated overnight at 4°C with primary antibodies listed in Table S6, and HRP-conjugated goat anti-mouse or -rabbit served as secondary antibodies (Vazyme). For immunofluorescence analysis, cells were fixed with 4%

formaldehyde and permeabilized before incubation with primary and secondary antibodies, each for 1 hr. Upon counterstaining with DAPI (0.5 μg/ml), samples were examined with an Imager.A1 (Zeiss) upright microscope to analyze specific gene expression.

## ***In vitro*** **cell phenotypic characterization**

For proliferation assays of cancer cells, 2 × $10^4$ cells were dispensed into 6 well-plates and treated with conditioned medium (CM) from stromal cells. The CM were collected from DMEM containing 0.5% FBS, with an equal number of stromal cells *per* condition. Three days post exposure to stromal CM, cancer cells were digested and counted with hemacytometer. For migration assays, cells were added to the top chambers of transwells (8 μm pore), while stromal CM were given to the bottom. Migrating cells in the bottom chambers were stained by DAPI 12-24 h later, with samples examined with Axio Observer A1 (Zeiss). Invasion assays were performed similarly with migration experiments, except that transwells were coated with basement membrane matrix (phenol red free, Corning). Alternatively, cancer cells were subject to wound healing assays conducted with 6-well plates, with healing patterns graphed with bright field microscope. For chemoresistance assays, cancer cells were incubated with stromal CM, with the chemotherapeutic agent MIT provided in wells for 3 days at each cell line's IC50, a value experimentally predetermined. Commercial EREG (human) recombinant protein (R&D Systems, Accession No. O14944, Cat. No. 1195-EP) was employed at 100 ng/ml as a positive control for cell-based assays.

## **Expression construct of fusion protein**

The cDNA encoding the EGF domain of human EREG was amplified by PCR and subcloned into a lentiviral vector pLenti-CMV/To-Puro-DEST2 (Thermo Fisher Scientific) to express the mature chain of EREG in PSC27 cells as a fusion protein. The expressed protein has an N-terminal hexahistidine (6 x His) tag, followed by

the EREG EGF domain fragment that corresponds to the residues D56-K116 of EREG complete chain (including signal sequence) of entries UniProtKB/Swiss-Prot: O14944.1 (locus EREG_HUMAN, accession O14944).

**Co-immunoprecipitation**

Cells were rinsed twice with cold PBS then lysed on ice for 20 min in 1 ml of lysis buffer (40 mM HEPES at pH 7.5, 120 mM NaCl, 1 mM EDTA, 10 mM pyrophosphate, 10 mM glycerophosphate, 50 mM NaF, 0.5 mM orthovanadate, EDTA-free protease inhibitors) containing 0.3% CHAPS. Four micrograms of antibody specific to EREG (Cell Signaling Technology) were added to the cleared cellular lysates and incubated with rotation for overnight. Then, 50 μl of protein A/G-agarose beads (Pierce) were added and the incubation continued for 12 h at 4 °C. Immunoprecipitates captured with the beads were washed thrice with the CHAPS lysis buffer and twice by wash buffer A (50 mM HEPES at pH 7.5, 150 mM NaCl, protease phosphatase inhibitors included), and boiled in 4 × SDS sample buffer prior to electrophoresis and immunoblotting.

**Experimental animals and preclinical studies**

All animals were maintained in a specific pathogen-free (SPF) facility, with NOD/SCID (Charles River and Nanjing Biomedical Research Institute of Nanjing University) mice at an age of approximately 6 weeks (~20 g body weight) used. Ten mice were incorporated in each group, and xenografts were subcutaneously generated at the hind flank upon anesthesia mediated by isoflurane inhalation. Stromal cells (PSC27 or HBF1203) were mixed with cancer cells (PC3, LNCaP or MDA-MB-231) at a ratio of 1:4 (i.e., 250,000 stromal cells admixed with 1,000,000 cancer cells to make tissue recombinants before implantation *in vivo*). Animals were sacrificed at 2-8 weeks after tumor xenografting, according to tumor burden or experimental requirements. Tumor growth was monitored weekly, with tumor volume (v) measured and calculated according to the tumor length (l), width (w)

and height (h) by the formula: $v = (\pi/6) \times ((l+w+h)/3)^3$ [5]. Freshly dissected tumors were either snap-frozen or fixed to prepare FFPE samples. Resulting sections were used for IHC staining against specific antigens or subject to hematoxylin/eosin staining.

For chemoresistance studies, animals received subcutaneous implantation of tissue recombinants as described above and were given standard laboratory diets for 2 weeks to allow tumor uptake and growth initiation. Starting from the 3rd week (tumors reaching 4-8 mm in diameter), MIT (0.2 mg/kg doses), DOX (doxorubicin, 1.0 mg/kg doses), therapeutic antibodies (cetuximab or EREG mAb, 10.0 mg/kg doses, 200 μl/dose) or vehicle controls was administered by body injection (chemicals via intraperitoneal route, antibodies through tail vein), on the 1st day of 3rd, 5th and 7th weeks, respectively. Upon completion of the 8-week therapeutic regimen, animals were sacrificed, with tumor volumes recorded and tissues processed for histological evaluation.

At the end of chemotherapy and/or targeting treatment, animals were anaesthetized and peripheral blood was gathered via cardiac puncture. Blood was transferred into a 1.5 ml Eppendorf tube and kept on ice for 45 min, followed by centrifugation at 9000 g for 10 min at 4 ˚C. Clear supernatants containing serum were collected and transferred into a sterile 1.5 ml Eppendorf tube. All serum markers were measured using dry-slide technology on IDEXX VetTest 8008 chemistry analyzer (IDEXX). About 50 μl of the serum sample was loaded on the VetTest pipette tip followed by securely fitting it on the pipettor and manufacturer's instructions were followed for further examination.

All animal experiments were performed in compliance with NIH Guide for the Care and Use of Laboratory Animals (National Academies Press, 2011) and the ARRIVE guidelines, and were approved by the Institutional Animal Care and Use Committee (IACUC) of Shanghai Institute of Nutrition and Health, Chinese

Academy of Sciences.

**Statistics**

All *in vitro* experiments were performed in triplicates. Animal studies were performed with 10 mice *per* group, without blinding measurements or arbitrary exclusions from analysis. Mice at an age of approximately 6 weeks (~ 20 g body weight) were randomly assigned to groups of placebo, chemotreatments and/or targeted treatments. Statistical analysis involving gene expression data was performed with R version 4.1.1 and BioConductor version 1.8. Unless otherwise indicated, data in the figures are presented as mean ± SD. Cox proportional hazards regression model and multivariate Cox proportional hazards model analysis were performed with statistical software SPSS (v23). Statistical significance was determined by unpaired two-tailed (two-sided) Student's *t* test, one- or two-way ANOVA followed by Bonferroni's post hoc tests. Pearson's correlation coefficients test, Kruskal-Wallis, log-rank test, Wilcoxon-Mann-Whitney test or Fisher's exact test. For all statistical tests, a $P$ value < 0.05 was considered significant. Experimental data meet the assumptions of the tests (normal distribution), and the variance was similar between experimental groups. All data were included, without specific exclusions throughout the study.

To determine sample size, we began by setting the values of type I error (α) and power (1-β) to be statistically adequate: 0.05 and 0.80, respectively [6]. We then determine *n* on the basis of the smallest effect we wish to measure. If the required sample size is too large, we chose to reassess the objectives or to more tightly control the experimental conditions to reduce the variance.

The investigators were not blinded to sample group allocations due to the fact that the genotypes of human primary cells, needed to be carefully documented by the investigators, so blinding was not always possible during experimental setup. When feasible, data analysis was performed blind, including RNA and protein

preparation, q-PCR and immunoblots, immunofluorescence staining, RNA-seq library preparation, bioinformatics profiling, tumor volume measurement, biochemical examination of circulating blood, evaluation of histological sections from preclinical biospecimens, for which all data acquisition was performed blinded to types of individual samples.

## References


1 Zerbino DR, Wilder SP, Johnson N, Juettemann T, Flicek PR. The ensembl regulatory build. *Genome biology* 2015; 16: 56.

2 Trapnell C, Roberts A, Goff L, Pertea G, Kim D, Kelley DR *et al*. Differential gene and transcript expression analysis of RNA-seq experiments with TopHat and Cufflinks. *Nat Protoc* 2012; 7: 562-578.

3 Nix DA, Courdy SJ, Boucher KM. Empirical methods for controlling false positives and estimating confidence in ChIP-Seq peaks. *BMC Bioinformatics* 2008; 9: 523.

4 Sun Y, Campisi J, Higano C, Beer TM, Porter P, Coleman I *et al*. Treatment-induced damage to the tumor microenvironment promotes prostate cancer therapy resistance through WNT16B. *Nat Med* 2012; 18: 1359-1368.

5 Chen F, Long Q, Fu D, Zhu D, Ji Y, Han L *et al*. Targeting SPINK1 in the damaged tumour microenvironment alleviates therapeutic resistance. *Nat Commun* 2018; 9: 4315.

6 Krzywinski M, Altman N. POINTS OF SIGNIFICANCE Power and sample size. *Nat Methods* 2013; 10: 1139-1140.

**Supplementary Fig. 1**

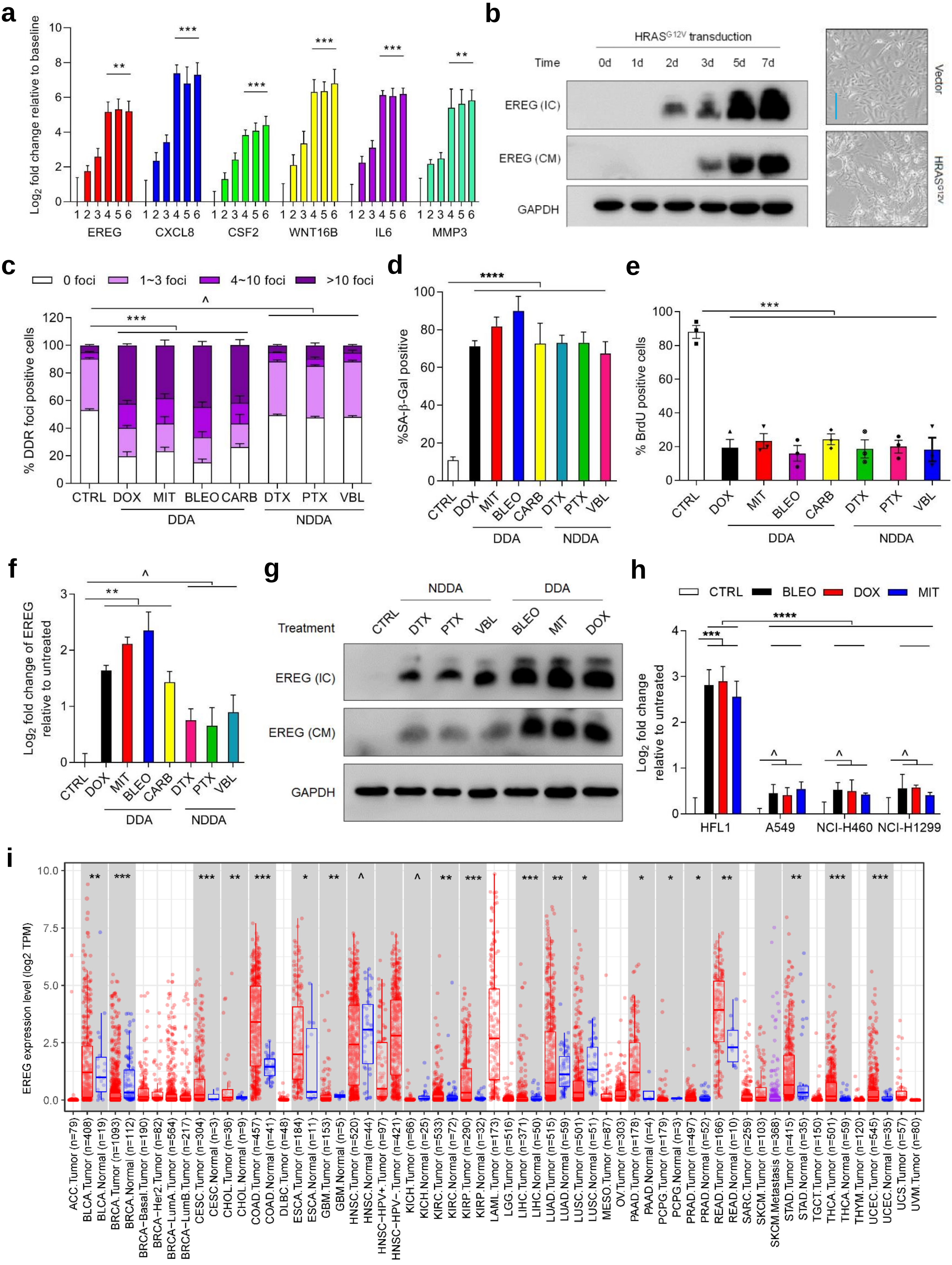

# Supplementary Fig. 2

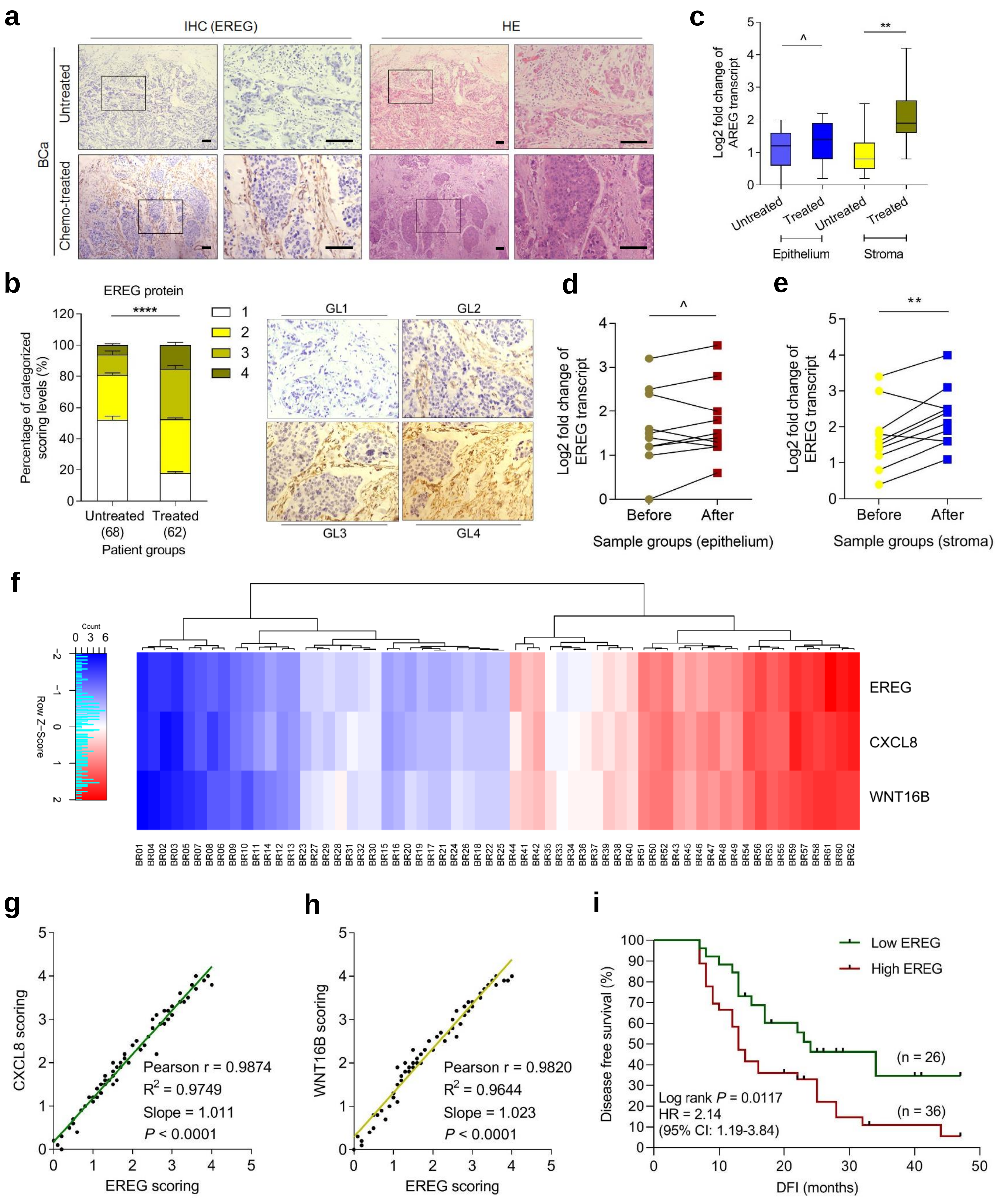

**Supplementary Fig. 3**

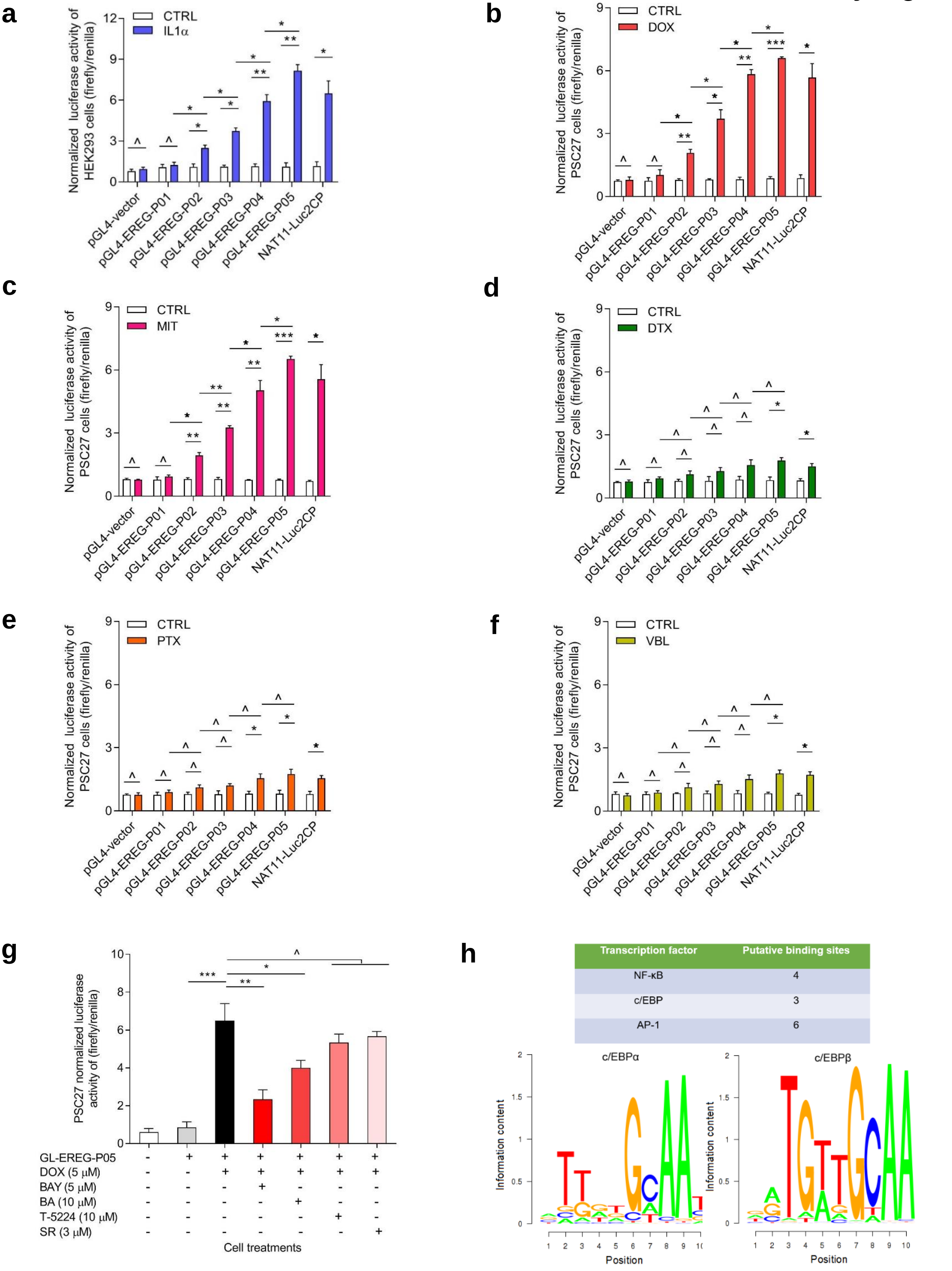


| Transcription factor | Putative binding sites |
|---|---|
| NF-κB | 4 |
| c/EBP | 3 |
| AP-1 | 6 |

# Supplementary Fig. 4

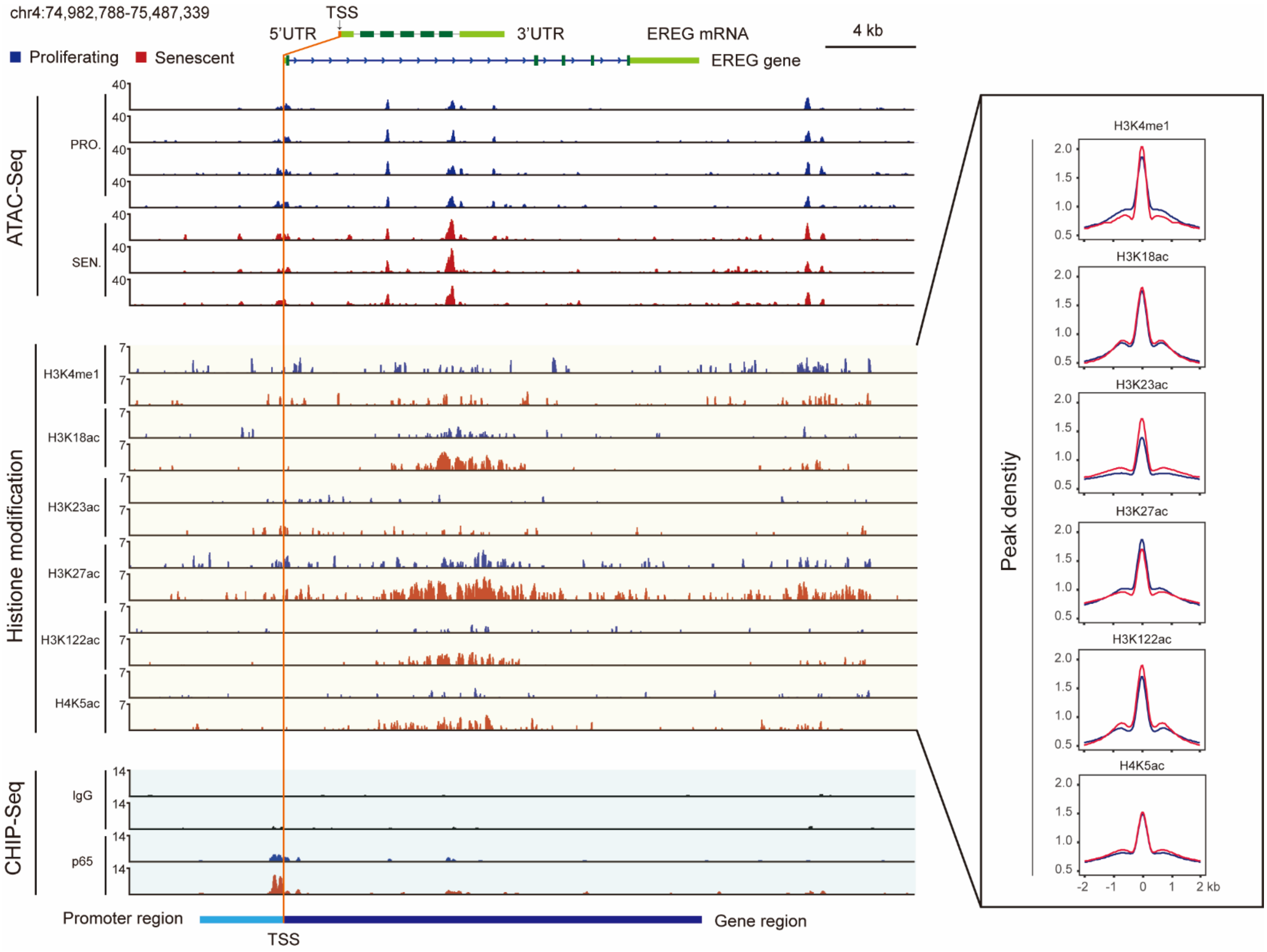

## Supplementary Fig. 5

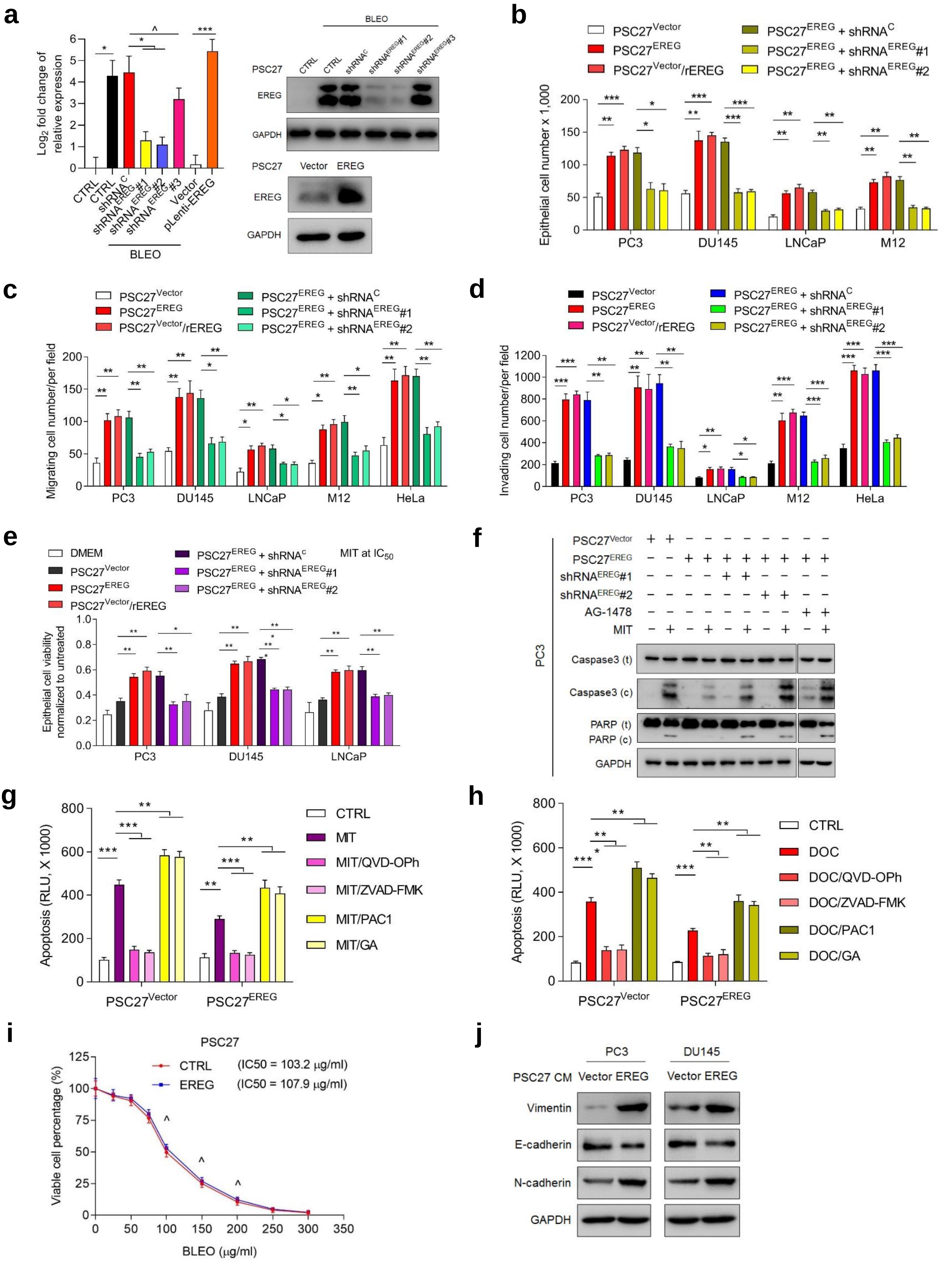

# Supplementary Fig. 6

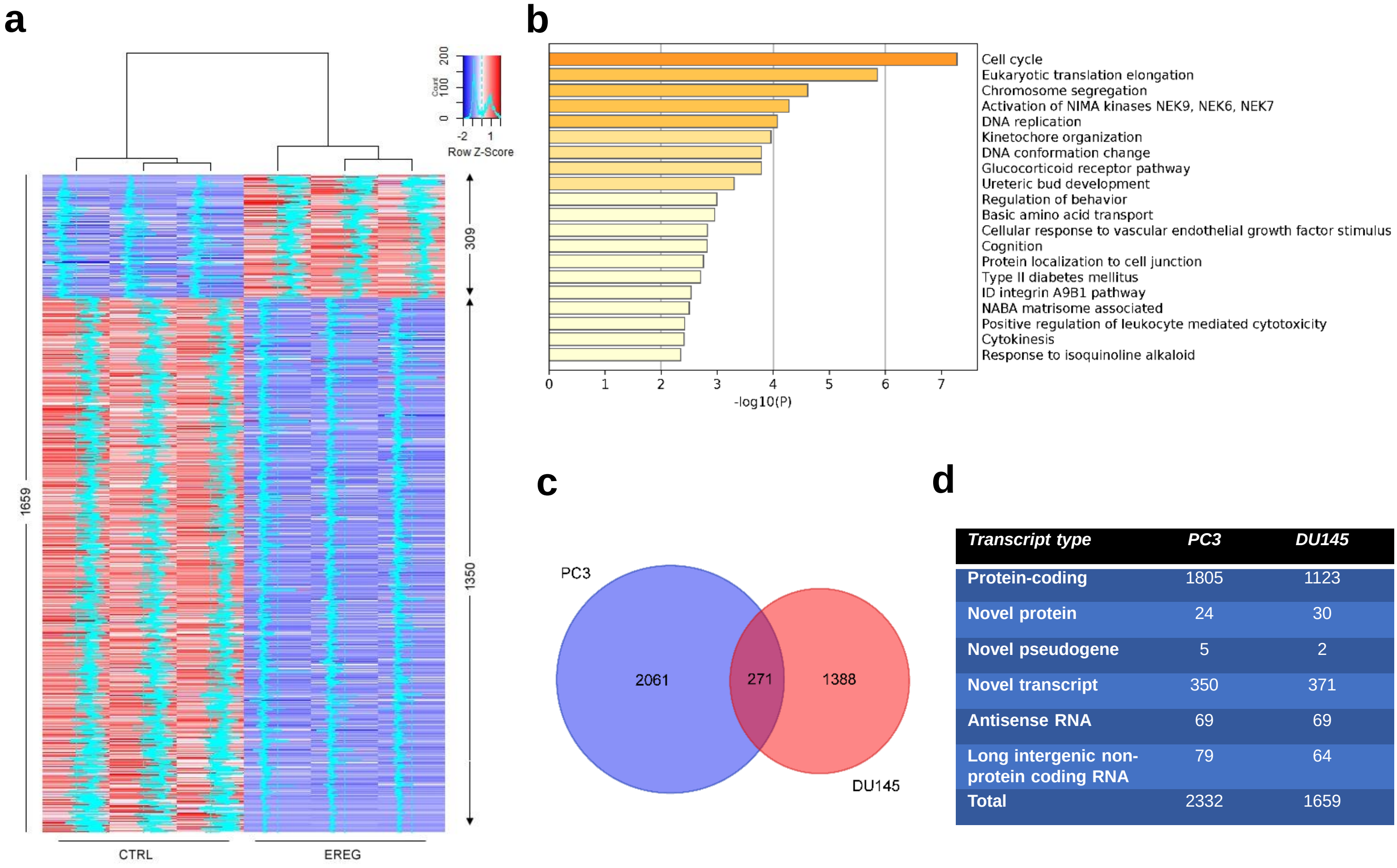


d

| *Transcript type* | *PC3* | *DU145* |
|---|---|---|
| **Protein-coding** | 1805 | 1123 |
| **Novel protein** | 24 | 30 |
| **Novel pseudogene** | 5 | 2 |
| **Novel transcript** | 350 | 371 |
| **Antisense RNA** | 69 | 69 |
| **Long intergenic non-protein coding RNA** | 79 | 64 |
| **Total** | 2332 | 1659 |

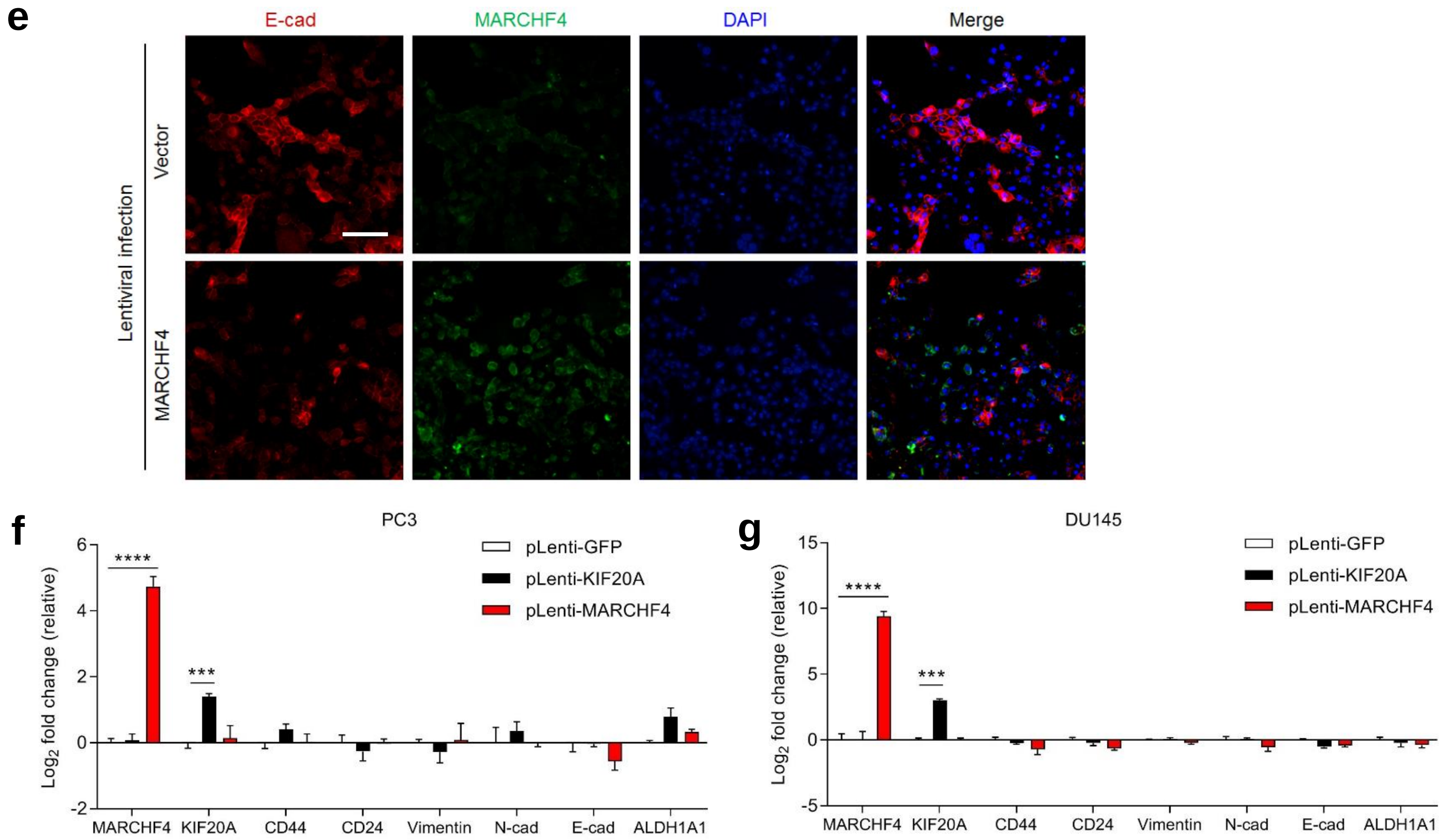

# Supplementary Fig. 7

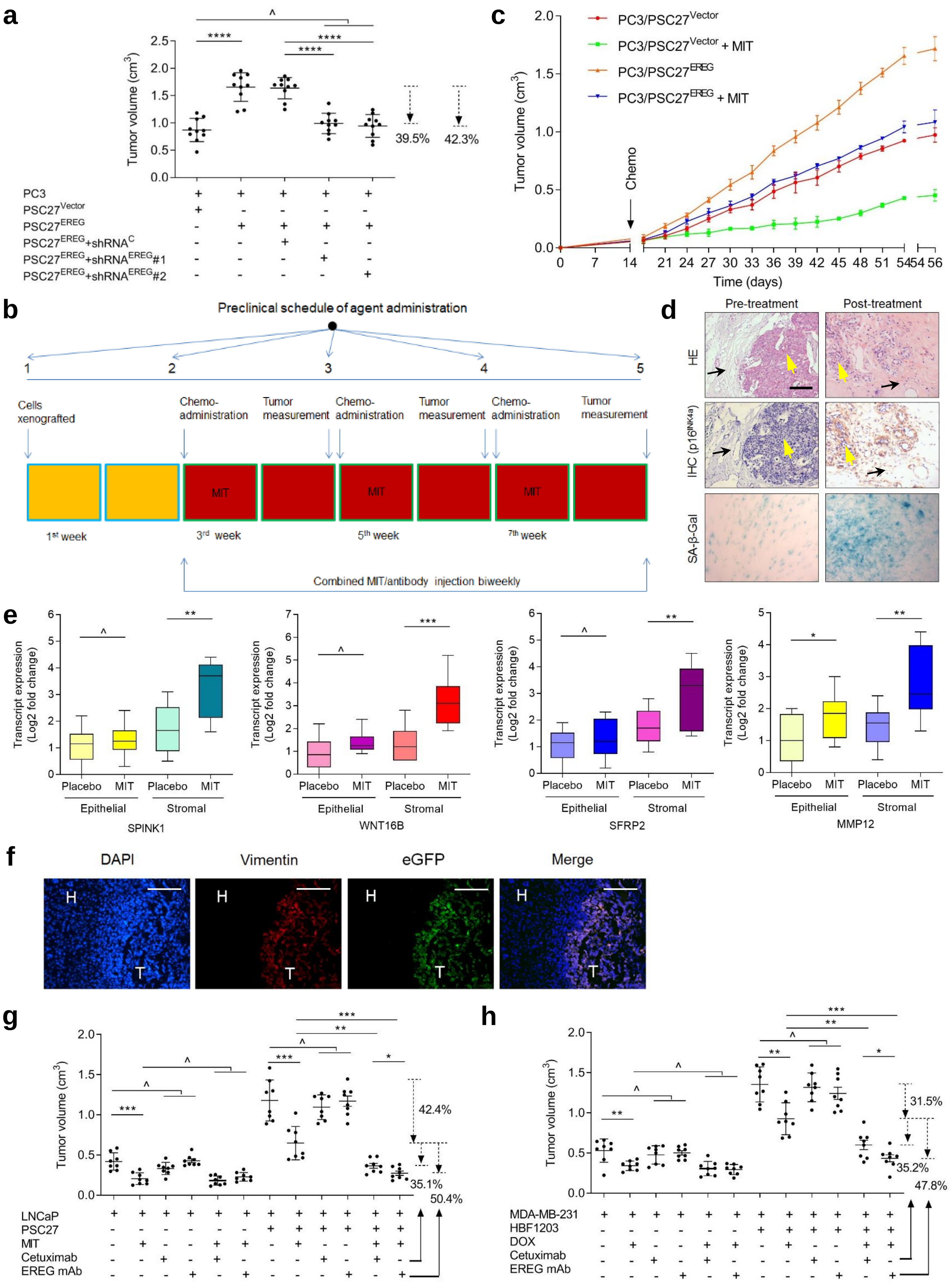

**Supplementary Fig. 8**

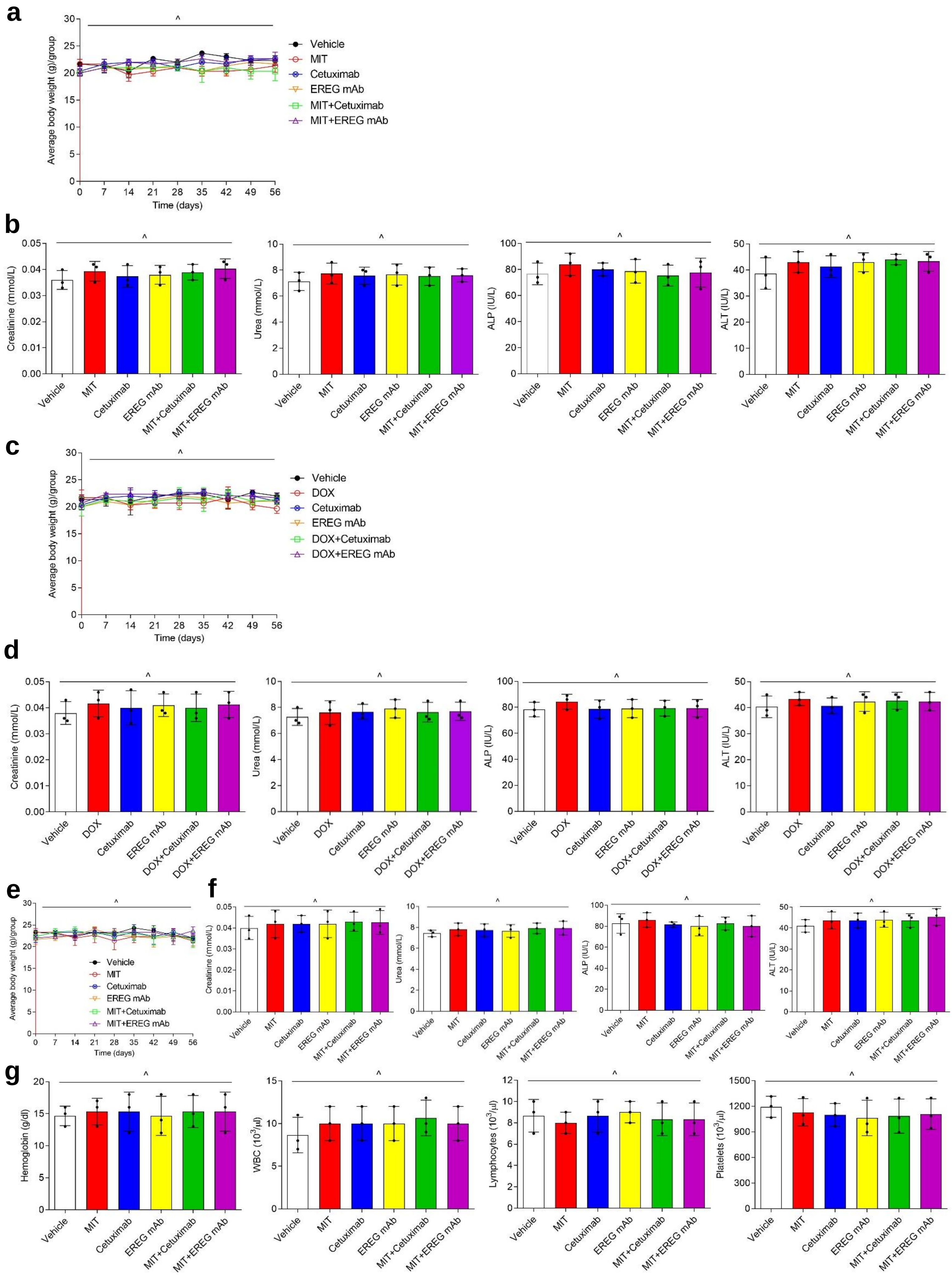

## Supplementary Fig. 9

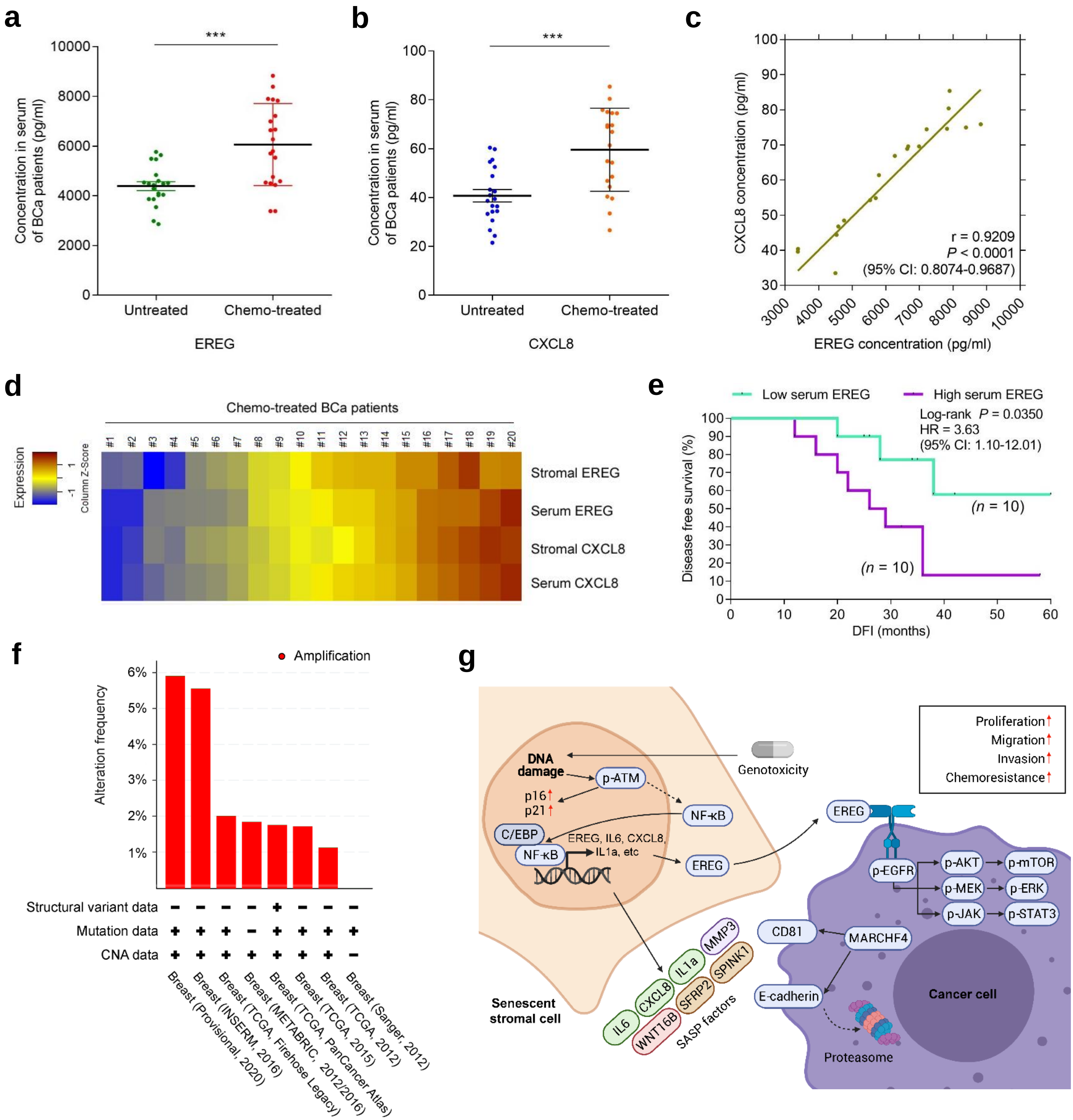

# Supplementary figure legends

**Supplementary Fig. 1 Characterization of EREG induction in human stromal cells and profiling of EREG expression in TCGA clinical samples.** (**a**) Quantitative RT-PCR measurement of EREG expression in the time course and other typical SASP factors in PSC27 stromal cells transduced with $HRas^{G12V}$ oncogene (OIS). 1, 2, 3, 4, 5 and 6, represent 0, 1, 3, 5, 7, 9 days after transduction, individually. (**b**) Immunoblot analysis of EREG expression in PSC27 cells upon OIS in the time course. IC, intracellular samples. CM, conditioned media. GAPDH, loading control. Right, representative images. Scale bar, 50 μm. (**c**) Measurement of DNA damage response (DDR) by quantification of γH2AX/p-53BP1 (co-staining) foci in HFL1 fibroblasts. CTRL, control. DOX, doxorubicin. MIT, mitoxantrone. BLEO, bleomycin. CARB, carboplatin, DTX, docetaxel, PTX, paclitaxel. VBL, vinblastine. DDR were classified into 4 subcategories, including 0 foci, 1~3 foci, 4~10 foci and > 10 foci per cell. DDA, DNA-damaging agents (DDAs). NDDA, non-DNA-damaging agents. (**d**) Assessment of cellular senescence by SA-β-Gal staining after treatment of HFL1 with agents described in (**c**). (**e**) Measurement of DNA synthesis by BrdU staining of HFL1 cells with agents described in (**c**). (**f**) Quantitative RT-PCR measurement of EREG expression at transcription level. Signals were normalized to CTRL. (**g**) Immunoblot analysis of the lysates of HFL1 cells treated as described in (**f**). (**h**) Comparative analysis of EREG expression in stromal cells (HFL1) versus a group of cancer epithelial cells (A549, NCI-H460 and NCI-H1299) after treatment with BLEO, DOX or MIT. Note all lines are of human lung origin. (**i**) Gene expression profiling interactive analysis (GEPIA) to profile EREG in the landscape of human solid tumor types. Data are based on tumor and adjacent normal samples from the TCGA and the GTEx databases. Distributions of gene expression levels are displayed using boxplots, with the number of patients provided in brackets per term. Samples available from both normal and tumor specimens of the same tumor type are plotted in gray columns. The statistical significance is computed by the Wilcoxon test and is annotated by the number of stars. OIS, oncogene-induced senescence.

Data are shown as mean ± SD and representative of 3 independent experiments, with at least 3 technical replicates per cell-based assay. *P* values were calculated by Student's *t*-test (**a**, **d**, **e**, **f**), two-way ANOVA (**c**, **h**) and Wilcoxon test (**i**). ^, $P > 0.05$; *, $P < 0.05$; **, $P < 0.01$; ***, $P < 0.001$; ****,

$P < 0.0001$.

**Supplementary Fig. 2 EREG is expressed in the tumor microenvironment (TME) of human breast cancer (BCa) patients after chemotherapy.** (**a**) Representative images of EREG expression in the primary foci of human BCa patients. Left, immunohistochemical (IHC) staining. Right, hematoxylin and eosin (HE) staining. In each staining set, top tissues were from untreated patients; bottom tissues from chemotreated (by doxorubicin, DOX). Rectangular region in the left image per staining was zoomed into the right image. Scale bars, 100 μm. (**b**) Pathological assessment of stromal EREG expression in BCa patients (untreated, 68; treated, 62). Patients were pathologically assigned into 4 categories per EREG staining intensity in the stroma. 1, negative; 2, weak; 3, moderate; 4, strong. Statistical comparison of untreated and treated groups is displayed. (**c**) Comparative analysis of EREG expression at transcript level between different cell lineages after chemotherapy. Briefly, epithelial and stromal cells were separately acquired by laser capture microdissection (LCM) from the primary tumors of BCa patients, with RNA subsequently extracted for quantitative assessment. (**d**) Statistical presentation of EREG induction in the epithelium of 10 randomly selected BCa patients. Stromal cells from both before and after treatment per same patient were isolated by LCM and subject to transcript examination. (**e**) Statistical appraisal of EREG induction in the stroma of the tumor foci of 10 randomly selected BCa patients. Cells were captured and processed in the manner similar to that described in (**d**). (**f**) Heatmap displaying pathological correlation between EREG, CXCL8 and WNT16B in the stroma of BCa patients after treatment. Scores were from the assessment of molecule-specific IHC staining, with expression levels colored to reflect low (blue) via modest (white) to high (red) signal intensity. Columns represent individual patients, rows different SASP factors. Totally 62 patients treated by chemotherapy were analyzed, with scores of each patient averaged from 3 independent pathological readings. (**g**) Statistical correlation between EREG and CXCL8 scores in the 62 tumors with matching protein expression data. (**h**) Statistical correlation between EREG and WNT16B scores in the same group of tumors described in (**g**). (**i**) Kaplan-Meier analysis of BCa patients. Disease free survival (DFS) stratified according to EREG expression (low, average score < 2, dark green line, n = 26; high, average score ≥ 2, dark red line, n = 36). DFS represents the length (months) of period calculated from the date of BCa diagnosis to the point of first time disease relapse. Survival curves generated according to the

Kaplan–Meier method, with *P* value calculated using a log-rank (Mantel-Cox) test. Data are shown as mean ± SD and representative of 3 independent experiments. *P* values were calculated by Student's *t*-test (**c**, **d**, **e**), two-way ANOVA (**b**), Pearson test (**g**, **h**) and log-rank test (**i**). ^, $P > 0.05$; *, $P < 0.05$; **, $P < 0.01$; ****, $P < 0.0001$. HR, hazard ratio.

**Supplementary Fig. 3 EREG expression is mediated by the NF-κB complex and C/EBP signaling in response to cytokine stimulation or genotoxic stress.** (**a**) Luciferase activity assay with lysates of 293T cells pre-transfected with each of GL-vector, GL-EREG-P01, GL-EREG-P02, GL-EREG-P03, GL-EREG-P04 and GL-EREG-P05 subsequently treated by 10 ng/ml IL-α in culture. Data are presented as relative ratios of firefly/renilla luciferase signals. (**b**) PSC27 cells transiently transfected with the constructs used in (**a**) were treated by DOX (doxorubicin, 5 μM), with cell lysates collected for luciferase assay. The NAT11-Luc2CP construct which encodes multiple NF-κB binding sites was used as a positive control. (**c**) PSC27 cells transiently transfected with the constructs used in (**a**) were treated by MIT (mitoxantrone, 2 μM), with cell lysates collected for luciferase assay. NAT11-Luc2CP used as a positive control. (**d**) PSC27 cells transiently transfected with the constructs used in (**a**) were treated by DTX (docetaxel, 50 nM), with cell lysates collected for luciferase assay. NAT11-Luc2CP used as a positive control. (**e**) PSC27 cells transiently transfected with the constructs used in (**a**) were treated by PTX (paclitaxel, 50 nM), with cell lysates collected for luciferase assay. NAT11-Luc2CP used as a positive control. (**f**) PSC27 cells transiently transfected with the constructs used in (**a**) were treated by VBL (vinblastine, 20 nM), with cell lysates collected for luciferase assay. NAT11-Luc2CP used as a positive control. (**g**) The reporter construct pGL-EREG-P05 encoding the longest approximal EREG promoter (3700 bp upstream of TSS) was transiently transfected into PSC27 cells before treated by DOX. BAY (Bay 11-7982, 5 μM), BA (betulinic acid, 10 μM) and T-5224 (10μM) were applied simultaneously with DOX as small molecule inhibitors against NF-κB, C/EBP family and AP-1, respectively. SR (SR 11302, 3 μM) was used as an extra control for AP-1 inhibition. Cells were lysed 7 d after treatment and subject to luciferase activity assay. Data were calculated as ratios of firefly/renilla luciferase signals. (**h**) Statistical summary of binding sites for transcription factors functionally associated with EREG expression in stromal cells. Upper, putative counts provided for binding sites of NF-κB, C/EBP and AP-1 on human EREG approximal promoter, with data derived from bioinformatics evaluation. Lower, representative binding motif of C/EBPα and

C/EBPβ. All data are shown as mean ± SD and representative of 3 independent experiments. *P* values were calculated by Student's *t*-test (**a**-**g**) (^, $P > 0.05$; *, $P < 0.05$; **, $P < 0.01$; ***, $P < 0.001$; ****, $P < 0.0001$).

**Supplementary Fig. 4** Landscape profiling of epigenetic regulation of EREG upon cellular senescence according to major findings of published literatures. For ATAC-seq, ChIP-seq and histone modification-associated profiles, raw data were downloaded from individual publicly available sources (GSE141992, GSE106146 for ChIP-seq, and GSE103588 for ATAC-seq), with deposited datasets analyzed individually to allow longitudinal visualization of the chromatin landscape encompassing EREG enhancer and approximal promoter regions [1-3]. All raw sequencing data were aligned against the hg19 version of the human genome using bowtie2, with HOMER employed to generate bigwig files. To allow assessment and overview of the genome-wide distribution of several histone modifications in proliferating *vs* senescent cells, peak densities of select histone methyl and acetyl marks identified in mass spectrometry studies (H3K4me3, H3K18ac, H3K23ac, H3K27ac, H3K122ac and H4K5ac) were plotted (see the right column, linked with histone modification presented in the left main figure), based on the datasets derived from ChIP-seq assays [2]. All data are representative of 3 independent experiments.

**Supplementary Fig. 5 Stromal cell-derived EREG changes the phenotypes of prostate cancer cells.** (**a**) Transcript assay (left) and immunoblot analysis (right) of EREG expression in PSC27 sublines established with lentiviral constructs. Cells were infected with lentiviral particles encoding either EREG-specific shRNAs (scramble as control) or full length human EREG sequence (vector as control). GAPDH, loading control for protein lysates. (**b**) PCa cell lines including PC3, DU145, LNCaP and M12 were treated with the conditioned media (CM) from PSC27 cells for 3 days, and subject to cell proliferation assay. rEREG, recombinant protein of human EREG. (**c**) Migration assessment of PCa cells after 3-day culture in the CM from PSC27 sublines. HeLa cells were examined as a positive control. (**d**) Invasion assay of PCa cells across the basement membrane of transwells upon culture with the CM from PSC27 sublines for 3 days. HeLa line served as a positive control. (**e**) Chemoresistance assay of PCa cells upon culture with the CM from PSC27 sublines. MIT (mitoxantrone) was applied at the concentration of IC50 value pre-determined per cell line. (**f**)

Immunoblots to analyze protein lysates prepared from PC3 cultured with the CM of PSC27 sublines for consecutive 3 days in the presence or absence of MIT. Intact and cleaved forms of caspase 3 and PARP1 were probed. GAPDH, loading control. (**g**) Apoptotic assay for combined activities of caspase 3/7 determined 24 h after exposure of PC3 cells to CM of stromal cells while being treated by MIT in the presence or absence of caspase inhibitors including QVD-OPH and ZVAD-FMK, or caspase activators including PAC1 and gambogic acid (GA). RLU, relative luciferase unit. (**h**) Apoptotic assay performed in the way similar as the one in (**G**), except that DOC (docetaxel) was applied instead of MIT. (**i**) Survival curves of PSC27 sublines transduced with either empty vector (CTRL) or an EREG construct (EREG). Cells were exposed to increasing concentrations of BLEO (0-300 μg/ml), and the number of surviving cells after 3-day consecutive culture was counted and normalized to the untreated control, with the IC50 value calculated per line. (**j**) Immunoblot analysis of EMT-associated factors in PC3 and DU145 cells treated by the CM from PSC27 sublines. Data are shown as mean ± SD and representative of 3 independent experiments. All $P$ values were calculated by Student's $t$-test (^, $P > 0.05$. *, $P < 0.05$. **, $P < 0.01$. ***, $P < 0.001$).

**Supplementary Fig. 6 Expression profile of prostate cancer (PCa) cells is subject to modification by stromal EREG.** (**a**) Heatmap showing differentially expressed human transcripts in DU145 cells after consecutive 3-day culture with EREG-containing CM collected from PSC27 stromal cells. In contrast to cancer cells cultured with control media (vector), there were 309 and 1350 genes upregulated and downregulated, respectively, upon treatment with CM from EREG-expressing PSC27 (EREG). (**b**) Graphical visualization of pathways by GO profiling. Genes significantly enriched in the upregulated list were sorted according to their fold change in DU145 cells exposed to the CM of EREG-expressing PSC27 cells. (**c**) Venn diagram presenting the overlap of 271 transcripts upregulated in PC3 and DU145 cells upon treatment with EREG-containing CM from stromal cells (2332 and 1659 genes were upregulated in PC3 and DU145, respectively). (**d**) A summary of differentially upregulated transcripts in PC3 and DU145 upon exposure to EREG-containing CM of stromal cells. (**e**) Representative images of immunofluorescence staining of PC3 cells. The construct encoding human MARCHF4 cDNA was transduced to PC3 cells, after which E-cadherin (E-cad) and MARCHF4 expression was examined by immunofluorescence microscopy. Scale bar, 10 μm. (**f**) Quantitative RT-PCR assay of a subset of cancer cell aggressiveness-correlated genes including

those encoding EMT markers. PC3 cells were transduced with KIF20A or MARCHF4, with GFP as an experimental control. (**g**) Quantitative RT-PCR assay for DU145 in a manner similar to that described for PC3 in (**f**). Data are shown as mean ± SD and representative of 3 independent experiments. *P* values were calculated by Student's *t*-test (**f, g**) (^, $P > 0.05$; ***, $P < 0.001$; ****, $P < 0.0001$).

**Supplementary Fig. 7 Stroma-derived EREG confers therapeutic resistance on solid tumors in a paracrine manner.** (**a**) Statistics of tumor volume measured at the end of an 8-week growth period. PC3 cells were xenografted alone or together with PSC27 cells to the hind flank of SCID mice. Prior to implantation, PSC27 cells were transduced with the control vector or an EREG construct to make stable sublines ($PSC27^{Vector}$, and $PSC27^{EREG}$, respectively). $PSC27^{EREG}$ cells were subsequently subject to lentiviral infection to make EREG-null sublines, scramble shRNA as control. (**b**) Strategic workflow of drug administration and tumor monitoring in the preclinical trial. PC3 cells alone or combined with PSC27 cells were inoculated subcutaneously to SCID mice 2 weeks prior to the initiation of chemotherapy. The chemotherapeutic agent MIT was provided on the first day of each week starting from the 3$^{rd}$ week, then given every other week with a total number of 3 doses. Therapeutic antibodies (cetuximab or EREG mAb) were given 12 h before each time of MIT delivery (totally 3 doses in the regimen). At the end of 8 weeks mice were sacrificed with tumor volume measured, and histologically analyzed. (**c**) Time course measurements of tumor growth in mice harboring xenografts comprising PC3 and PSC27 sublines. Measurements were performed from the 18$^{th}$ day post xenograft implantation, with a frequency of once *per* 3 days, until the end of the regimen (8 weeks, 56 days; the last measurement was on the 56$^{th}$ day, which was the ending time of regimen). The timepoint of chemotherapeutic administration of MIT is indicated. (**d**) Representative images of *in vivo* cellular senescence after MIT-mediated chemotherapy. PC3/PSC27 tumors were collected at the end of therapeutic regimen and subject to histological assays. Images from staining of HE, IHC (anti-$p16^{INK4a}$ as a primary antibody) and SA-β-Gal staining were acquired for comparative analysis. Black arrows, stromal cells. Yellow arrowheads, cancer cells. Scale bars, 150 μm. (**e**) Transcript analysis of a subset of canonical SASP factors expressed in stromal cells isolated from the tumors of SCID mice. Tissues from animals implanted with both stromal and cancer cells in tumor grafts were subject to LCM isolation, RNA preparation and qRT-PCR assays. (**f**) Immunofluorescence

assessment of tumor xenografts. Tumor sections were subject to staining with an antibody against vimentin. Blue, DAPI; red, vimentin; green, signals from PSC27 cells pre-transduced with a marker vector encoding enhanced green fluorescent protein (eGFP). H, host; T, tumor. Scale bars, 150 μm. (**g**) Statistical comparison of tumor growth in animals after treatment by different agents. Mice received LNCaP cells implanted alone or combined with PSC27 cells, before treatment by the chemotherapeutic drug (MIT) only or combined with cetuximab or EREG mAb. Tumor volumes were measured at the end of an 8-week preclinical regimen. (**h**) A similar statistical comparison performed for tumors grown in mice as depicted in (**g**), except that animals received breast cancer cells (MDA-MB-231) alone or combined with breast stromal cells (HBF1203). Mice were treated by the chemotherapeutic drug DOX only or together with monoclonal antibodies (cetuximab or EREG mAb). Tumor volumes were measured at the end of an 8-week preclinical regimen. Data are shown as mean ± SD and representative of 3 independent experiments. N = 10 per treatment arm. MIT, mitoxantrone. DOX, doxorubicin. $P$ values were calculated by Student's $t$-test (**a**, **e**, **g**, **h**) (^, $P > 0.05$; *$P < 0.05$; **$P < 0.01$; ***$P < 0.001$; ****$P < 0.0001$).

**Supplementary Fig. 8 Chemotherapeutic and/or targeting agents generate negligible effects on body weight, biochemistry, and blood counts of experimental mice.** (**a**) Mouse body weights were determined once a week until the end of the therapeutic regimen. Chemotherapeutic agent MIT (0.2 mg/kg) was administered alone or with the targeting antibody (cetuximab or EREG mAb, each 10.0 mg/kg) on 1st day of week 3, 5 and 7 after tumor implantation (PC3/PSC27) to SCID mice. (**b**) Terminal bleeds were taken via cardiac punctures on day 56. Serum levels of creatinine, urea, alkaline phosphatase (ALP) and alanine aminotransferase (ALT) were analyzed for toxicity appraisal with SCID mice developing prostate tumors. (**c**) Mouse body weights were determined as described in a strategy similar to (**a**). Chemotherapeutic agent DOX (1.0 mg/kg) was administered alone or with the targeting antibody (cetuximab or EREG mAb, each 10.0 mg/kg) on 1st day of week 3, 5 and 7 after tumor implantation (MDA-MB-231/HBF1203) to SCID mice. (**d**) Terminal bleeds were taken via cardiac punctures on the last day of the 8-week regimen. Serum levels of creatinine, urea, alkaline phosphatase (ALP) and alanine aminotransferase (ALT) were analyzed for toxicity appraisal with SCID mice developing breast tumors. (**e**) Mouse body weights were determined once a week until the end of a therapeutic regimen applied to immunocompetent animals. Chemotherapeutic agent

MIT (0.2 mg/kg) was administered alone or with the targeting antibody (cetuximab or EREG mAb, each 10.0 mg/kg) on 1st day of week 3, 5 and 7 to wild type C57BL/6 mice. (**f**) Terminal bleeds were taken via cardiac punctures on the last day of the regime. Serum levels of creatinine, urea, alkaline phosphatase (ALP) and alanine aminotransferase (ALT) were analyzed for potential toxicity to C57BL/6 mice. (**g**) Blood counts were measured to evaluate potential effect of therapeutic agents on the immune system and tissue homeostasis of C57BL/6. WBC, white blood count. Data are shown as mean ± SD and representative of 3 independent experiments. n = 3 per treatment arm. MIT, mitoxantrone. *P* values were calculated by Student's *t*-test (**a-g**) (^, *P* > 0.05).

**Supplementary Fig. 9 EREG is a novel blood-borne biomarker that reflects the SASP development *in vivo* and predicts adverse outcome of cancer patients.** (**a**) Abundance of EREG protein in the serum of untreated and chemo (DOX)-treated BCa patients. Data were derived from ELISA measurement, n = 20. (**b**) Abundance of CXCL8 protein in patient serum analyzed in (**a**), data from ELISA assays, n = 20. (**c**) Scatterplot correlating EREG and CXCL8 in the serum of individual BCa patients studied in (**a**) and (**b**). Pearson's correlation coefficient, *P* value and confidence interval are indicated in the plot, n = 20. (**d**) Heatmap profiling the overall correlation between stromal EREG, serum EREG, stromal CXCL8 and serum CXCL8 in chemo (DOX)-treated BCa patients (n = 10). The raw scores of stromal EREG and CXCL8 were derived from independent pathological reading of primary tumor tissues of BCa patients, with those of serum EREG and CXCL8 obtained from ELISA assays. Color key, relative expression of these two factors in stromal tissue or patient serum. (**e**) Kaplan-Meier survival analysis of chemo (DOX)-treated BCa patients. Disease free survival (DFS) stratified according to EREG expression in tumor stroma (low, average score < 2, cyan line; high, average score ≥ 2, purple line). DFS represents the length (months) of period calculated from the date of chemotherapy to the point of first time disease relapse. Survival curves generated according to the Kaplan–Meier method, with *P* value calculated using a log-rank (Mantel-Cox) test. N = 10 per group. (**f**) TCGA data showing alterations of EREG in human BCa patients at genomic level (mainly amplification). Alteration frequency is displayed in percentage. (**g**) Illustrative working model of EREG expression in the treatment-damaged TME, pathological impact of paracrine EREG on intercellular signaling network of cancer cells and its potential as a therapeutic target and novel biomarker for cancer surveillance and outcome prediction in clinical settings.

## References

1 Liu P, Li F, Lin J, Fukumoto T, Nacarelli T, Hao X *et al*. m(6)A-independent genome-wide METTL3 and METTL14 redistribution drives the senescence-associated secretory phenotype. *Nat Cell Biol* 2021; 23: 355-365.

2 Sen P, Lan Y, Li CY, Sidoli S, Donahue G, Dou Z *et al*. Histone Acetyltransferase p300 Induces De Novo Super-Enhancers to Drive Cellular Senescence. *Molecular cell* 2019; 73: 684-698 e688.

3 Parry AJ, Hoare M, Bihary D, Hansel-Hertsch R, Smith S, Tomimatsu K *et al*. NOTCH-mediated non-cell autonomous regulation of chromatin structure during senescence. *Nat Commun* 2018; 9: 1840.

**Supplementary Table 1. Univariate and multivariate Cox proportional hazards model analysis of prognostic factors for PFS of PCa patients.**

| Variable | Univariate | | | Multivariate | | |
|---|---|---|---|---|---|---|
| | HR | 95% CI | *P* | HR | 95% CI | *P* |
| Age: < 60 vs. ≥ 60 | 0.49 | 0.21–1.35 | 0.120 | 0.46 | 0.24–0.98 | **0.038** |
| Tumor stage: IV vs. IIc-III | 1.31 | 0.40–4.16 | 0.699 | 1.38 | 0.51–3.92 | 0.562 |
| Tumor size: ≥ 3 vs. < 3 cm | 1.63 | 0.69–3.46 | **0.042** | 2.12 | 1.14–4.34 | **0.039** |
| EREG: low vs. high | 0.79 | 0.36–1.68 | **0.018** | 0.35 | 0.21-1.69 | **0.006** |
| Caspase 3 (cleaved): low vs. high | 0.53 | 0.18–1.72 | 0.269 | 1.25 | 0.42–4.24 | 0.096 |
| AR low vs. high | 1.12 | 0.63-1.91 | 0.059 | 1.31 | 0.69-3.64 | 0.068 |

PFS, progression-free survival. PCa, prostate cancer. HR, hazard ratio. CI, confidence interval. AR, androgen receptor. $P < 0.05$ is statistically significant, with significant *P*-values highlighted in bold.

**Supplementary Table 2. Univariate and multivariate Cox proportional hazards model analysis of prognostic factors for PFS of BCa patients.**

| Variable | Univariate | | | Multivariate | | |
|---|---|---|---|---|---|---|
| | HR | 95% CI | *P* | HR | 95% CI | *P* |
| Age: < 60 vs. ≥ 60 | 0.41 | 0.18–1.10 | 0.098 | 0.45 | 0.21–0.84 | **0.049** |
| Tumor stage: IV vs. Ia-III | 1.42 | 0.34–4.12 | 0.524 | 1.35 | 0.42–3.68 | 0.454 |
| Tumor size: ≥ 3 vs. < 3 cm | 1.80 | 0.48–3.25 | **0.046** | 1.98 | 1.09–3.19 | 0.068 |
| EREG: low vs. high | 0.68 | 0.35–1.75 | **0.014** | 0.46 | 0.25-0.81 | **0.006** |
| Caspase 3 (cleaved): low vs. high | 0.67 | 0.25–1.89 | 0.228 | 1.56 | 0.39–3.22 | 0.106 |
| HER2 high vs. low | 2.46 | 1.78-3.16 | **0.016** | 1.89 | 1.26-2.83 | **0.036** |
| PR high vs. low | 2.95 | 0.95-3.92 | 0.074 | 1.56 | 0.96-2.78 | 0.106 |

PFS, progression-free survival. BCa, breast cancer. HR, hazard ratio. CI, confidence interval. HER2, human epidermal growth factor receptor 2. PR. Human progesterone *receptor*. $P < 0.05$ is statistically significant, with significant *P*-values highlighted in bold.

**Supplementary Table 3. List of significantly upregulated genes in PCa cell lines upon exposure to stromal EREG.**

| Characteristics | Gene numbers | Gene names (as output elements) |
|---|---|---|
| Overlapping between PC3 and DU145 lines | 39 | *CCNB1 SLC26A10 A1CF BIRC5 PLK1 SAMSN1 B3GALT5 AL136981.3 FGF1 SMC4 AURKA KIF14 AL050303.3 GALR2 AC016773.2 CNRIP1 AC036214.1 MARCHF4 CDC20 SCEL BUB1 NUF2 CPED1 DEPDC1 CENPE PPP1R14B-AS1 AL138724.1 SUV39H1 MND1 SPNS2 CCNB2 CU633906.5 BUB1B MKI67 NAV3 KIF20A MROH6 SERTAD4-AS1 CCDC183-AS1* |
| Unique to PC3 line | 931 | *ESPL1 C3orf14 AC026740.1 RYR3 MIR137HG AL121749.1 E2F8 AC092747.2 PTN AC021092.1 UTP20 C17orf97 ZNF492 LINC00643 AC099066.2 PDCL3 AC100800.1 HSPB6 AC016583.2 AC078883.1 ARHGAP11A AC116158.3 OR2B6 GJD3 TPX2 MMP7 AC016394.1 DEPDC1B GYS1 CYCSP6 ZNF85 C15orf48 AC048382.5 HRG LINC01708 FAT4 HROB NDUFAF4 MSRB3 PDE1C MMS22L MSMP DOCK2 AC024560.5 AL357054.4 PDLIM2 CCDC190 AL499602.1 FAM95B1 MNS1 AC107959.3 NLRP3 NPM1P25 ZFP3 ST6GALNAC5 SFXN2 RFC3 CD33 CCDC34 BRCA1 GEN1 AC010809.1 PTPRG-AS1 FARSB TGM2 GINS1 CLDN11 ARPP21 PHACTR3 H2BC13 TEK MCUB TRPM6 KPNA2 FKBP14-AS1 DDX11L16 RPP40 LINC01127 AC112721.1 DSCC1 ANLN SHISA3 PRR16 WDR54 LINC02027 SELL NPM1P9 GALNT13 IFITM3P1 CCDC141 AC005865.2 ZNF345 AL359922.3 AC022973.5 KNTC1 AC066580.1 CMTM1 CFAP157 FOXM1 SLC16A12 ALK ANGPT4 AL031320.2 LMLN IPO4 HMGB1P5 FAS PM20D2 AC023024.2 SPRN CDK1 REP15 SEMA3E CLEC2B CHEK1 AL136964.1 NEIL3 KPNA2P3 KIF11 BCL2 PARVA PART1 FAM72B ORC1 LRRC4C MUC7 NUP37 MPP4 CENPH TMPRSS11E GPD1 KIF18B AC232271.3 CLCN4 PCAT5 GPI DNAH11 ASF1B NLRP10 PACRGL AL807752.3 STMN3 TMEM200A AC114798.1 AC092919.2 PRR22 CDK6-AS1 FAM216A GPR19 HVCN1 E2F1 AC010894.1 AC087883.2 PPBP OXTR CDC25C AC022107.1 SUV39H2 PSMC3IP PTRH1 AC131392.2 LINC01116 ARHGAP33 SNRPF DAPP1 BBS1 CDC6 C12orf4 AC068831.6 AC008494.3 COLGALT2 PPIAP22 WDR62* |

| | | |
|---|---|---|
| | | *CRPPA KIFC1 CENPU ZWILCH AC015813.5 CCDC18 KCNQ5 AC002310.2 MYBL2 UNG AC091057.1 MASTL HEPHL1 HSPA4L APOBEC3B AL161756.1 GNB4 C2orf81 LINC00943 GALNT18 MT-CYB CENPO KIAA1755 SRD5A2 MGAT5B CIP2A LONRF2 AL049840.4 TUBA1B AC009549.1 ARMC6 PXMP2 DHFR MAD2L1 ZFHX4-AS1 PLAGL1 ELFN1 SPATA3-AS1 AC079601.1 C4orf36 AC083799.1 H3C1 POLA1 MAGOHB AURKB ARHGAP28 CDCA7L OXCT1 LRRC66 NR2C2AP AL451064.1 POLD2 INGX MCM3 HECW2 VPS9D1-AS1 MIR503HG TIPIN CCDC15-DT AC069499.1 AC091826.2 BNIP3 HMGB3P7 LEF1 MTHFD1 CHAF1A SERPINB2 MTG1 AL139011.1 CCN2 ZNF107 GRWD1 SLC25A13 CTSS POC1A MRTO4 AL049840.5 FANCA DEUP1 LINP1 PDSS1 IL23A ZGRF1 KIF20B JPH2 GAS2L3 NCAPD3 BLM DNLZ LINC02709 RPS2P46 LINC02261 UCN2 KIF4A PDGFB VIM NCAPG2 AC027228.2 AL078612.2 XRCC3 SAMD9L PCNA MMACHC GPATCH4 Z97832.2 DCLK2 AP000944.5 H2AC17 PHF19 DPYSL4 AC069547.2 CCDC134 HASPIN HERC6 CENPS KRTAP1-5 AC005775.1 CCL20 AC005786.3 PLA2G4C BARD1 PARPBP F2 STX11 CA9 CYP7B1 OTUD7A SPDL1 HMGB1P8 GPR176 ALDH1B1 MAP6D1 ARMC4 FAM86JP ACOD1 SLC35E1P1 CEP83 KIF5A KIF2C AADACP1 NEGR1 EGFLAM AC006299.1 MCM10 TCF19 MT-CO3 CDC42EP2 AL390726.4 PLD5 TTLL11-IT1 FBXO5 AC025678.3 CCR5AS LINC01291 WDR4 CD83 SLC7A5P2 HIRIP3 IL4I1 FAM72D TICRR DLG1-AS1 CHTF18 AL513477.1 TRAIP AC004982.2 C20orf141 CDK4 CENPA LDHAP3 TMEM75 FLI1 TYMS CDCA7 MRPS9 IGHEP2 THG1L MCM5 DENND2A CLN6 AP001528.2 HERC3 MELK DPY19L2 GPR1 OGFRP1 FAM53A ZNF551 DLEU2 AL355612.1 TDRD9 GAP43 AL133390.1 CENPN KRTAP4-8 OR10A6 RIMS1 AC112777.1 FAM30A UTP14A STARD13-AS MAP6 AL133551.1 ZWINT LAMA1 THOP1 TUBB3 NDC80 GIMAP6 TADA2A AC073264.3 ZIC3 MTND5P11 LPAL2 SGO1 ABHD16B KRT34 RAB36 ORC5 LCMT2 PLK4 AC112236.1 TNKS2-AS1 OIP5 CCNA2 GASAL1 HAUS8 ZNF724 GTSE1 DYNC2H1 WDR7-OT1 INHBB GLYCTK MIR3150BHG AC026316.5 Z94721.3 POLQ GAL PBK AL161891.1 PSRC1 LINC01111 AADAT PRR11 SKA1 AC007326.4 NIM1K AC004801.2 AC091057.6 TRIP13* |

| | | |
|---|---|---|
| | | *AC044810.2 AC069224.1 AC012313.5 AC127526.5 BEND5 IL6 AC011043.1 TCAF2P1 TROAP HMGB2 EPHB1 ADAMTS6 CDCA5 FAM242C CDC7 FBXO15 MORC2-AS1 TAS2R14 FBXO43 TRIM6 CEP164P1 TP53I3 SLC22A20P EEF1A1P11 LYPD1 PES1 TEAD4 MX2 ZNF215 PLPPR5 NXT1-AS1 RAB39B CREB5 ITGB3BP PARP1 AC008581.1 LINC02084 KIF23 AC114271.1 ARHGDIB TRIP6 ZNF695 SKA3 NCAPH TAGLN FAM111A IQGAP3 MYBL1 AGAP14P ACOT6 TK1 DIAPH3 AC004832.1 LETM2 SGPP2 BRI3BP RFPL4AL1 AC018521.5 AC024587.1 EPHA3 PYCR3 TREX2 TAS2R31 PFAS ALOXE3 SMC2 RMI1 SHCBP1 CDRT1 NOL4 MTFR2 MMP3 ARL9 GUSBP5 ASPM SACS AXL LMNB1 UBE2S STARD6 RFC2 AP000311.1 SPAG5 ASL BRCA2 CMSS1 TMSB15A HTR2B LRRC37A9P PRIM1 AC007731.1 MMP8 LINC02057 NAP1L3 PCLAF C7orf57 CDCA3 AL663070.1 HMGN2 AC009879.3 PIMREG SMKR1 RALGAPA1P1 PRSS2 NOP56 MRM1 KNL1 NUDT11 ATAD2 ZNF229 AP003307.1 CHRNA1 ALOX5AP DNMT1 AC004982.1 BRIP1 FAM172BP RUFY1-AS1 TACC3 SCG2 AC011043.2 NDST3 AC063952.1 STIL NME7 UBE2C MYL3 GINS4 MIR924HG CHML DPY19L2P1 IL1R2 AL096711.2 ANKRD30B PCDH19 CBR3 MINDY4 ZNF10 POLE2 ARHGAP19-SLIT1 PRC1 LINC00920 IGFBP1 AC012038.2 TCAF2 DOK3 PXDNL FIGNL1 AC110015.1 SOGA3 PIF1 SORCS3 TRDMT1 CDT1 AC011503.1 RAB40A PUS7 LINC00571 WDR35 CENPW LINC01968 RIBC2 GBP1 PSMG1 CEP55 PALMD MPHOSPH6 DIAPH2-AS1 LINC00461 WDR77 HNRNPM AL356274.2 QTRT1 SPRR2A C2orf72 AUNIP CORO1A RRM2 PHACTR2 AC015849.5 DDIAS EVI2B RASSF2 C19orf48 TOP2A SCARA3 ADAM1A AL021920.2 FEN1 JSRP1 PPIF ROBO4 ELOVL2 KLK5 NRGN CXCL9 THBS1 SCLT1 ATRIP CASC17 HELLS H2AX FANCI PIK3R2 LOXL1-AS1 KRTAP4-9 DRD5P2 LINC02236 DBF4B CCL28 RAD54L CCNE2 ZNF367 DPF1 CDC45 CYTOR C4orf47 NEXN SUGT1P4-STRA6LP LINC02009 PLEC AC135782.1 AC079385.3 AC011603.3 GVINP1 APLN IRAG1 SPC25 ARHGAP31-AS1 LIG1 FAH WDHD1 DNTTIP1 TERT MCM2 IKBKE TMEM200C TRAF1 FAM72A SOCS1 PKMYT1 AL121987.2 MCM4 DUT BLZF2P KIF18A AC060814.2 SPA17 LINC00052 C22orf24 KIF15 KRTAP2-3 AC037198.2 DYNC1I1 INMT* |

| | | |
|---|---|---|
| | | *ADSL POP1 SAA1 PLAAT1 AL359834.1 CCDC96 GLIDR SLAMF7 TIMELESS AC092745.4 RRM1 RPL37A-DT FAM189A2 DLGAP5 WDR76 TMEM14B GPRASP1 HJURP RAD51AP1 AL359258.3 HAT1 SLITRK2 AQP1 L3HYPDH KIF24 IL7R ESCO2 ZSWIM5 TLR4 SPDYE9 SAMD15 ENO1 AC021945.1 SLF1 MB21D2 CKAP2L AC067930.2 SYDE1 ADAMTS3 H2AC7 LURAP1L-AS1 AC117481.1 CCDC15 AL512353.2 AL450426.1 GLI2 AC008780.1 CSNK2A3 AC139720.2 CLSPN DTL FAM83D AC137936.2 MANCR DZIP1L C15orf56 ERVMER34-1 EME1 CDCA2 HHIP CIT RAD51 ZNF469 CENPP PAICS SLC2A4RG AC145423.3 SERPINB10 AC005041.1 HMMR AL008721.2 AC084357.3 TGM4 TCTN2 CAMKV HDHD5 AC010457.1 CHAF1B SLC35E3 TEDC1 EXO1 LINC00378 ACACB EBNA1BP2 MCM6 MIR4432HG AC007106.1 CCDC171 RAB39A STAMBPL1 CENPI CU638689.4 LAP3 WDR18 GIMAP5 GMNN NMU AP002784.1 TEDC2 KCTD16 DIRC3-AS1 AC036108.2 SOWAHA CCBE1 NUAK2 AC114803.1 ERCC6L KMO GPR63 ORC6 CENPK SMG1P7 KRT5 AC114811.2 GAS6-AS1 LINC00911 LINC02827 C1QTNF1-AS1 MAP7D3 CXCR6 FCRLA CFAP300 TUBGCP5 HPSE AC145207.5 PIGW PDCD1LG2 EXOC3L1 PLAC4 H3C3 SAMD9 AC010327.4 TRBC1 CLUAP1 LINC01234 NT5C3AP1 DAW1 EXOSC6 NRAV ANKRD1 SAA2 AC091849.2 SCNN1G LINC00702 AP000526.1 LCAL1 MEIOC PRICKLE1 RBFA RNVU1-26 AC005077.4 BRD3OS LINC00680 CEP128 HNRNPA1P33 RIMBP3C UHRF1 FAM111B RRP15 NECTIN3-AS1 CHN1 SLC2A9 SGO2 AC100822.1 AC099548.2 AC067751.1 CHAC2 AC004947.1 ACOX2 BNC2 NANOS1 AC109322.2 ATAD5 NRK ASB9 RPL9P25 BACH2 QTRT2 AP002851.1 TTK PSMB10 AC022034.1 IVL TRNP1 AC108449.2 CDKN3 AC008543.1 NME1 FAM3D AL669831.7 SLC4A4 FJX1 SCG5 AL590399.1 NCAPG RFC5 AC087632.2 AL360181.2 AOX1 LINC02742 GCNT1 DTNA DUSP9 C21orf58 RMI2 BPTFP1 AC018978.1 AC022364.1 AC024132.1 ZNF658B H2AC20 TCOF1 AL354920.1 COQ2 LINC02672 AC013468.1 VRK1 ADD2 TEX15 NEK2 SKA2 CENPM DPYSL3 FANCB FLVCR2 GINS2 RASSF1-AS1 RBL1 CCDC189 LINC00242 SPC24 OXCT2P1 CENPF H1-10-AS1 NUSAP1 KRT75 TTC26 CD3EAP FLRT1 ABCA13 DPCD FANCG RDM1 SNCB* |

| | | |
|---|---|---|
| | | *FANCD2 HECW1 METTL1 POLR3B DHRS2 CDC25A CDCA8 XRCC2 ESM1* |
| Unique to DU145 line | 270 | *ST8SIA5 AC060766.1 XDH AL136985.3 SNHG16 AC093827.1 YBX2P1 GAS5 TECTA ZBTB12 AL390728.2 HMGB3 LRMDA ABALON AC005674.2 AL596244.1 TRIM22 AC104109.3 CA14 CD160 SEC14L5 AC005540.1 YOD1 AC089984.1 CALML4 NDUFC2-KCTD14 LGALS1 AC091152.2 ALDH2 AC004801.6 LINC02019 CR769775.2 AC139530.2 AC097059.1 MYH6 AC133644.2 FBXL19-AS1 TAS1R3 AC022154.1 HESX1 OR6L2P AC084757.3 TMEM156 AL645922.1 TNFRSF11B MYADML2 AC002996.1 AP002990.1 QARS1 CTBP2P10 TERC ALG14 IRS1 RPS27A AC013472.2 NME1-NME2 AF111169.1 AL049555.1 AC007842.1 SCAT8 IGFL2-AS1 RSKR AP002847.1 GDAP1L1 RPSA IL1RL1 AL122035.1 RPL22L1 KRT15 FAM215A HK3 AC019069.1 CTBP2P9 TCEA1P4 TRAPPC2B CCNP AC120057.3 CCL27 MYBPH LEPR AC009053.3 AC124862.1 AC131392.1 RPL32P29 EIF4BP7 RPL4P2 AC008764.4 FHL2 LTB AL590714.1 RNF185-AS1 AC008770.3 SLC38A2 AL662884.5 RGPD2 PLAC9 AL137784.1 LRRN4 AL138781.1 AC087164.2 PRDM6 EPHX4 KCNG2 CCNT2-AS1 AC087623.2 PAPPA2 CGB2 CHMP3 AC025423.4 OTP AC007308.1 AC004877.1 RBM3 RPL13AP5 ZNF807P AC092687.3 AC022532.1 AP003068.4 AL139094.1 AC138811.2 EEF1B2 PER3 ACTRT3 S100A6 APEX1 AL035458.2 CAVIN2 RPL6 AP000688.1 ZBED6 LINC01213 MALL SRSF9P1 GTF2IRD2P1 AC137630.3 CEMP1 SLC23A3 AC007881.4 EXOSC5 AL645940.1 SLC11A1 AC022826.2 AC004832.3 DHFRP1 AC012213.5 UFC1 KCNH1-IT1 ADAMTS12 AC005329.1 AP000350.6 SLC7A2 OR11M1P GREM2 AC026786.1 ZDHHC11 SERPINB8 BDH1 RPLP0 HMGN2P5 AP001347.1 AC016877.3 F13A1 AC025262.2 RPL41P1 CTRC PSMC1P1 TAP2 LHX4 SNHG5 AL929091.1 DMBT1 AC135983.4 SKOR1 NAIPP1 AC006435.4 AC020661.4 SLC22A13 HK2 AC106886.2 AC016877.1 AC105020.4 RN7SL1 AC008403.2 TUBA1C PTPRG ZNF256 AC009093.8 C2 RPL6P27 AC020922.3 AL353796.2 ARL5C GRPR AL110118.2 EIF3L AC023794.5 AC009812.4 ZRSR2P1 PDE4B ODC1 FAM243A TCTE1 SMARCE1P6 NDUFB2-AS1 COL17A1 AC125611.3* |

| | | |
|---|---|---|
| | | *ACTB VN1R1 SLC49A3 AC132812.1 ARG2 ICAM3 SLC41A3 AC024580.1 AC010327.2 MIF-AS1 AP001972.5 AL135999.1 RPL41 AF127577.6 AC021016.2 KLF15 AC024267.6 ZNF286B LINC02263 AL445524.1 LRRC46 VEGFD AL137002.2 POU5F1 LINC02262 EDN2 AC141586.4 FOSB UBE2Q2L NHLH1 RPL23A FUT5 AL133500.1 ANAPC15 IL11 COL24A1 TTC9B AL512274.1 AL356017.1 YARS1 KISS1 AC005041.3 AC010531.1 Z85996.2 RPL12P37 RPS14 ACTG1 AC006486.2 FP671120.4 IMPDH1P5 AC000120.4 MAP2K3 BHLHE41 AC124312.3 AC006059.5 AC093668.1 SPHK2 AL603832.2 AC005670.2 ACTBP13 TMEM81 AC084880.1 SLFN12L TUBBP1* |

**Supplementary Table 4. List of differentially expressed genes in PCa cell lines upon exposure to stromal EREG.**

| Characteristics | Gene numbers | Gene names (as output elements) |
|---|---|---|
| Overlapping between PC3 and DU145 lines | 271 | *CSPG4 SSC4D NTRK1 AC133555.5 GDF15 OR2B6 NTSR1 AL137800.1 CCNB1 AC104024.4 SLC26A10 AC100803.3 C5AR1 COL9A2 CCDC153 A1CF BIRC5 APOE ALDH2 PRDM16 B3GNT7 FZD9 PA2G4P4 GNAT1 MYO15B SAMSN1 ID1 B3GALT5 CYP1A1 AC010319.2 CNTFR CETN4P FGFR3 FGF1 CAPN12 MAFA SELENOP AURKA SRD5A2 RARRES2P4 CPAMD8 KIF14 AP002907.1 GALR2 PPP1R1B TNFAIP6 AC016773.2 TNF DEUP1 RPL32P29 SEMA3G WNT6 AC008738.7 RNF185-AS1 H2AC17 KANK3 AL133335.2 H3C6 AGGF1P2 PLAC9 LINC01907 CYP4F12 MARCHF4 CILP2 AC004877.1 CD72 NYAP1 AL133551.1 ENPP2 AC026316.5 AQP6 AC239584.1 TMEM178A BGN NUF2 AC005753.3 AL669831.3 DEPDC1 GGT1 CDRT1 CENPE AC104024.3 KIAA0408 AP003733.3 PPP1R14B-AS1 GPR20 AL138724.1 CCNB2 PALM3 RGS11 TRPV4 FBLN2 TMEM221 AC120498.4 CDC42EP5 AC008040.1 PIWIL2 C2 AC000085.1 IRAG1 STRA6 PDE4B AC211476.7 GIPC3 ARG2 MKI67 NAV3 MAP1LC3A AC105118.1 GRIN3B RTBDN TMEM59L HAPLN3 NKILA MROH6 BCL6B C1QTNF1-AS1 CEACAM1 COL8A2 AC005077.4 P2RX7 MAP1LC3B2 SULT2B1 AP002851.1 GLP2R AC009229.4 HSD17B6 CCDC183-AS1 AC026956.2 HYAL1 AP003120.1 HPN DELEC1 LDHD PROM2 AL136131.2 AC107959.3 PSG8 H2BC13 AL139352.1 AC010504.1 PLK1 LINC02019 CR769775.2 FOXC1 AC090192.2 TNFSF15 CACNG7 AC131212.2 TNNI3 IGFALS KLHDC7B SCN4A ARHGAP4 IGFL2-AS1 AL136981.3 NUPR1 SMC4 C2orf81 SYNE4 PRSS35 FOXO6 MCEMP1 SPATA3-AS1 AL050303.3 LEPR H1-3 PKD1L2 INS-IGF2 NPTX2 RNF225 AC040160.1 NCCRP1 CNRIP1 NTF4 DES AC008083.2 CENPS MAP6D1 KIF5A ATP1B2 AC036214.1 CFAP58-DT AP003068.4 CDC20 PRSS3 FKBP1B SLC17A7 SCEL AL391832.2 BUB1 TMPRSS2 CPED1 AC046185.3 C1QL4 AC005329.1 SLC7A2 SHD NMUR1 MMP28 AC107294.2 AL034417.2 PDE2A RANBP3L C7orf57 CNIH2 IGF2 CLIC3 SUV39H1 LY75 CYGB MND1 DMBT1 SPNS2 LINC00920 CALHM3* |

| | | |
|---|---|---|
| | | *NAT16 AP000424.1 APOBEC3H CCDC33 EFEMP2 LOH12CR2 AC021218.1 LINC02236 PLA2G4F CYP1B1-AS1 SUGT1P4-STRA6LP AC116535.1 KCNK15 TEX48 SERHL2 FLJ31356 CU633906.5 LINC01589 TMEM249 CEBPA BUB1B GPRASP1 LYNX1 C21orf62-AS1 AC008780.1 INAFM1 AL008721.2 CHD5 AL137785.1 VEGFD RGMA AC036108.2 KIF20A AP003068.1 LINC02175 FUT5 LINC00886 IL20RB PTGS1 AC091849.2 AL512274.1 AC015712.6 RNF213-AS1 LINC01970 AC025164.1 STUM AC022364.1 SERTAD4-AS1 CYP24A1 AC069185.1 RASSF1-AS1 PTGIS TINCR SNCB GRIP2* |
| Unique to PC3 line | 2061 | *PNCK CLMP AC026740.1 INSL3 KCNJ2 AC092747.2 ARHGAP5-AS1 UTP20 TACC2 ZNF492 CYP11A1 AC099066.2 ABCC6 PDCL3 HSD17B1 CMTM8 AC016583.2 CRTAM KRT16P2 AP004609.3 LDB3 AL445623.2 PELI2 GJD3 TPX2 HMGCS2 GNAL MMP7 RDH10 SIRPB2 AC016394.1 GYS1 AC004908.1 C15orf48 PADI1 HRG LINC00520 LINC01708 FAT4 CCDC88B HROB DIRAS1 NDUFAF4 MSRB3 PDE1C CEACAM6 MMS22L MSMP AC012459.1 PDLIM2 CCDC190 GPR143 BBC3 NR4A2 AL499602.1 CXCL13 FRY MNS1 SLC22A2 GPR153 CCDC34 CYP2F1 AC087289.2 BRCA1 GEN1 AC010809.1 PTPRG-AS1 AC068888.1 GINS1 ACSM3 AL031123.1 AL139023.1 APOBR ANAPC1P2 TEK MCUB TRPM6 AL136531.3 KPNA2 FKBP14-AS1 DDX11L16 CLDN16 MACROD1 RPSAP5 CA11 AC112721.1 DSCC1 ANLN HSPA1L SCX ARL14EPL PRR16 SLC1A7 LINC02569 SELL AC093799.1 LTBP4 SPIRE2 DNAH17 IFITM3P1 IFITM3P2 ZNF345 KNTC1 FGF17 PKDREJ GPR78 CFAP157 FOXM1 ANGPT4 KLF3-AS1 NR2E3 IPO4 PCNPP3 SLC6A15 SYT13 CDK1 DNM1 AP002370.1 FAM182B DPP9-AS1 AL009031.1 PPM1K SERPING1 BCL2 AF127577.4 ORC1 LRRC4C SLC36A3 NUP37 MPP4 AL355987.4 A4GALT GPD1 KCNK15-AS1 CLCN4 GCK AP002748.3 MMP15 DNAH11 ASS1 NLRP10 AL807752.3 TMEM200A AC114798.1 PDE4C AC092919.2 AL136418.1 PRR22 FP700111.2 RHOH BAMBI TLCD2 GPR19 HVCN1 FP325332.1 AKR1B15 AC010894.1 RYR1 CHST6 SMAD9 TLCD3B RGL1 AC087883.2 PPBP OXTR AC022107.1 SUV39H2 PSMC3IP AC131392.2 AP001528.1 ZNF467 SLC38A4 HES7 KRT37 LGI4 PTGES AP002761.2 DAPP1 BBS1* |

| | | |
|---|---|---|
| | | *CDC6 C12orf4 SULT1C2 AL592295.4 VASH1 WDR62 CRPPA CLEC3B EPHA4 CENPU AC023906.3 PLA2R1 AC015813.5 CCDC18 KCNQ5 UQCRHL AC002310.2 AC245407.2 TARID LINC01444 UNG RPLP1P11 HEPHL1 APOBEC3B GNB4 PACERR LINC00943 GALNT18 TLE2 RBM44 REM2 CIP2A LONRF2 AL049840.4 GIPR BST1 LINC00486 STON1 ADSS1 AC009549.1 ARMC6 PXMP2 DHFR MAD2L1 ZFHX4-AS1 PHACTR1 AC103810.2 ELFN1 ATP1A1 RARA-AS1 FRMPD3 CYSTM1 EML5 AC083799.1 AC233968.1 H3C1 FAM229A AC097526.1 STRC IGKV1OR-2 TJP3 MAGOHB MILR1 SNN AC009404.1 AURKB HID1 SOWAHB AC067968.1 POLD2 PLSCR4 MIR503HG ZMIZ1-AS1 TIPIN CCDC15-DT CCL26 LGALS7B AC091826.2 RALGPS1 ZDHHC11B AL158212.4 MTHFD1 CHAF1A HRH2 SMIM38 MTG1 PRSS22 AL139011.1 SLCO1B3-SLCO1B7 ATP6AP1L PRR36 CEBPD ZNF107 GRWD1 SLC4A5 AC073111.3 POC1A MRTO4 AL049840.5 FANCA PCBP3 AP001266.1 PDSS1 IL23A ZGRF1 EDAR JPH2 EGF GNRH1 NCAPD3 AC008537.2 FST VWCE ESPNL LINC01374 AL133227.1 AC141586.5 FAM9B LINC02261 UCN2 KIF4A CCDC169-SOHLH2 MYOZ2 SNCAIP AC090365.1 KLHL28 AL078612.2 RCAN1 ZNF705E Z97832.2 NTN4 DCLK2 AP000944.5 AC016820.1 UCA1 DPYSL4 TMPRSS4 AC084262.2 AC069547.2 HASPIN HERC6 KRTAP1-5 AL512504.1 NSG2 AC091167.1 MYCL AC005786.3 RAB15 TNNT2 AP002490.1 PLA2G4C BARD1 F2 PTGES3P1 CA9 OTUD7A SPDL1 HMGB1P8 AC099552.4 TSPAN19 FDFT1 PTGS2 SLC35E1P1 LINC02359 DMRTC1B AADACP1 ADAMTSL4 SQLE NEGR1 AC066614.1 SAT2 MCM10 TCF19 AC025678.3 GCM1 PCDHGA5 TLR5 LINC01291 CLIP2 LINC01194 CD83 SLC7A5P2 AC114744.2 NPY AC005329.3 CYP2A7 CTSK IL4I1 SGK2 SC5D SLC9A2 FAM72D NEURL3 CHTF18 LINC00507 RASSF9 BMP2 NPY1R MVD AL513477.1 PCDH15 NPIPB7 HPCAL4 AP001267.2 TRAIP C20orf141 AC015802.4 CDK4 LINC01179 CCDC85B LDHAP3 FLI1 TYMS MRPS9 IGHEP2 IGFN1 AC133919.2 NOL4L MCM5 DENND2A CLN6 AP001528.2 GPRIN2 SH2D3C HERC3 MELK DICER1-AS1 PTP4A3 GPR1 AC079140.6 FAM53A GPX2 RAMP3 ZNF551 DLEU2 ESRRB AC025165.1 LINC00882 AL109811.2 TDRD9 AC015563.2 GAP43* |

| | | |
|---|---|---|
| | | *LINC-PINT KRTAP4-8 OR10A6 AC083862.1 RIMS1 AC112777.1 BCL6 AL022318.2 CDKN2B AC124319.1 MAP6 ZWINT LAMA1 MFAP3L NDC80 GIMAP6 AREG AL031432.2 ZBTB7C SLC6A16 ACKR2 AC073264.3 ZIC3 LPAL2 SLC5A3 SGO1 ABHD16B ADRA1D NTS KRT34 SCART1 AC007686.5 ORC5 LCMT2 AL031731.1 DOCK9-DT TNKS2-AS1 OIP5 CCNA2 GASAL1 CU639417.5 TIMP1 GTSE1 ALPK1 KLF9 SPDYE8 PCSK9 GAL APOBEC3F THEMIS2 PPP1R3F CD86 AL161891.1 LINC01978 PSRC1 PLA2G10 LINC01111 PDK4 AC145212.1 RESF1 AC091057.6 SPINK13 AC044810.2 AC069224.1 AC012313.5 BEND5 IGFBP5 NRTN TCAF2P1 AL158071.4 ZMYND10 EPHB1 SLC44A3-AS1 ADAMTS6 IL18RAP CDCA5 FBXO15 MORC2-AS1 LSAMP ATP2A3 AC016571.1 ZIK1 TRIM6 TP53I3 OR7D2 HAGLR SLC22A20P PES1 TEAD4 ZNF215 ZNF862 PLPPR5 UCP2 FGFBP1 RAD21L1 TBATA CACNA2D2 PCDHB6 AC008581.1 LINC02084 KIF23 OLFM2 MMP11 CD14 ITGA10 ARHGDIB CDK18 TRIP6 CAPN8 MCF2L AL359878.2 AC136489.1 ZNF695 FAM111A DNAJB13 IQGAP3 MYBL1 SHC2 ZNNT1 VASH2 ACOT6 DIAPH3 AC004832.1 AC092117.1 BRI3BP HAS3 AC018521.5 BMF PYCR3 GRAMD1B CALML5 FNDC4 TAS2R31 PCED1B PFAS ALOXE3 GAREM1 AL132986.1 FCGRT U4 NOL4 AL645941.2 MTFR2 ZNF555 MMP3 GUSBP5 AXL LMNB1 AL121928.1 PNPLA3 SPAG5 ASL AC011472.1 PALM BRCA2 BMS1P10 KCNQ4 CMSS1 LRRC37A9P SLC44A3 KRT8P26 SLC5A7 AC007731.1 MMP8 ABCA10 WBP2NL SYCP2L LINC02057 PCLAF HMGN2 CCDC3 FOXN3 SMKR1 KIAA0513 PRSS2 AC018638.4 ANXA10 MRM1 PGF GOLGA8Q AL163952.1 AP003307.1 AL589182.2 AL391684.1 FYB1 KLK7 ALOX5AP RUFY1-AS1 SYNDIG1L TACC3 RPSAP52 SCG2 CDON TRIM73 PSMC1P7 LVRN TNFSF11 THSD7A NDST3 NBEA AC063952.1 AC119674.2 LINC00334 TESPA1 MYL3 LINC01694 AL162497.1 MRPL23-AS1 B4GALNT3 CHML DPY19L2P1 AL096711.2 CBR3 FAM226B KLHL38 MINDY4 AC116021.1 ZNF10 AQP3 ST6GALNAC4 TBC1D3G PRC1 LINC00589 AC011498.4 PLCD1 AC012038.2 CEACAM5 AC061975.1 AC093677.3 CLEC7A ZNF56 NECTIN4 SOGA3 PIF1 SORCS3 TRDMT1 NAPSA CDT1 ERP27 LINC00571 TRHR RNF152 CENPW RPSAP18* |

| | | |
|---|---|---|
| | | *LINC01968 MUCL1 GCOM1 GBP1 PSMG1 CEP55 PALMD KRT20 MPHOSPH6 DIAPH2-AS1 KCNG3 FAM71E1 GNE BX284632.2 WDR77 AC010442.3 SHISA4 NOX3 QTRT1 AC005064.1 C2orf72 ARHGEF4 AC008083.3 KRT16 PHACTR2 DDIAS EVI2B TNFSF8 GABRP SCARA3 AL021920.2 ROBO4 ELOVL2 AC090515.2 CBX7 STARD4 NRGN MYOC SCN1B THBS1 SCLT1 ACSL1 H2AX AC073610.2 CTXND2 PIK3R2 LOXL1-AS1 AC016727.3 AC008267.1 KRTAP4-9 AC010978.1 AC100774.1 AC004980.2 CCNE2 DPF1 CDC45 NANOGNBP2 AKR1B1 GRID2 AC011603.3 AC005393.1 LINC01833 GVINP1 APLN CFL1P1 LINC00598 AC025165.6 GSDMB LIG1 NID2 ADCY10P1 TERT AC006511.5 MCM2 TMEM200C AC096751.1 AP000829.1 AL391294.1 FAM72A AC084262.1 CALM2P2 SOCS1 AL121987.2 MCM4 BLZF2P HTRA1 AC060814.2 KLHL31 SPA17 C22orf24 AL831711.1 LINC01285 RPS5P2 AC037198.2 AL121581.2 C4orf51 JMJD1C-AS1 POP1 SAA1 PLAAT1 SBK1 AL354751.1 CCDC96 AL391844.1 AC021148.3 AL691432.1 GNG7 SLAMF7 TIMELESS PLIN5 AC092745.4 PRPH RPL37A-DT DLGAP5 MSMO1 TMEM14B LINC02761 FAM182A HTR1D RAD51AP1 HAT1 SLITRK2 L3HYPDH ESCO2 AC026471.1 TSPAN18 SAMD15 ENO1 Z97989.1 AL158163.2 C20orf203 BFSP1 MB21D2 CKAP2L AC003965.2 AC067930.2 MYZAP SYDE1 ADAMTS3 MED12L H2AC7 LURAP1L-AS1 LEKR1 ERO1B AL450426.1 GLI2 AC099329.2 C1QTNF12 CLSPN AC137936.2 MANCR GDE1 AC090921.1 DZIP1L AC004233.2 ERVMER34-1 SULF1 EME1 RNU7-53P CDCA2 TGFBR3 PBXIP1 LURAP1L LINC00184 AKR1C1 ZNF469 CENPP SLC2A4RG PLPP1 AC084357.3 SLC6A12 TGM4 AC010457.1 ISLR2 TPM2 EXO1 ACACB KCNJ2-AS1 EBNA1BP2 RAB39A STAMBPL1 CENPI AC105345.1 LAP3 AGMO PPP1R1A ABCB11 AC104852.1 MXD1 NMU TEDC2 DIRC3-AS1 LINC01612 SOWAHA CCBE1 NUAK2 RND2 INHA LMOD2 AL139099.1 OIP5-AS1 KMO AC073610.1 SOD2-OT1 GPR63 JAKMIP3 AIFM3 CENPK PPIAP46 SMG1P7 SEMA3F SLC27A2 GAS6-AS1 AC090337.2 CPE CXCR6 CFAP300 TUBGCP5 CD200R1 AC008760.2 TPPP HPSE AC145207.5 PDCD1LG2 SCRT1 NHSL2 ARHGEF25 H3C3 AC034236.2 AC007991.4 HMGCS1 SMIM10L2A AC010327.4 TESK2 NT5C3AP1* |

| | | |
|---|---|---|
| | | *AC104532.1 CCDC159 RN7SKP70 AL031777.1 DKKL1 SCNN1G AC132938.6 AC136475.2 AP000526.1 LINC01204 LCAL1 HMGB1P31 AC006378.1 SEMA6B NCOA7 ODF3B LINC00680 CEP128 CNNM2 SUCNR1 TMEM269 FAM111B AC138907.8 RRP15 AC131011.1 FZD4 AC005532.1 SERPINB3 AC008397.1 AC004947.1 BNC2 C8orf74 HOXB7 NRK ASB9 AL138478.1 CBY3 BACH2 AL138889.3 AC111149.2 MINAR1 LBH HSD17B14 SLC25A41 AC022034.1 IVL LINC01001 AC092825.1 AC108449.2 NME1 FAM3D OMG SLC4A4 AL590399.1 AL133355.1 CALCOCO1 ZBTB46 HSD3BP5 RFC5 AP001029.2 AL133351.4 AC087632.2 GJB4 FBXO2 AOX1 DTNA AC018978.1 AC024132.1 AC013268.1 ZNF658B TCOF1 GJA5 AL354920.1 LINC02672 AC013468.1 INSIG1 AC133528.1 KCNE4 TEX15 APOC1 RNASE4 CDK3 TMEM37 NEK2 PDE4A SATB1 YPEL3 LINC01801 LINC00592 FLVCR2 AC087491.1 GINS2 CYP1B1 SAT1 SPC24 OXCT2P1 ADAMTS7 MTAPP1 YPEL2 COLQ PDCD6IPP2 AC132872.1 KRT75 TTC26 FAM171A2 SLC19A2 ALPK2 CD3EAP ABCA13 BEAN1 FANCG BCL2L14 RDM1 ADGRB1 CERS4 FANCD2 HECW1 CCDC13 METTL1 IGFL1 BICDL2 AC024896.1 CDC25A XRCC2 ESM1 ESPL1 C3orf14 AC008763.2 MIR137HG RYR3 AL121749.1 E2F8 ZAP70 PTN AL138690.1 OR7M1P OTUD1 SPINK1 AC021092.1 C17orf97 AC108676.1 LINC00643 KRT18P55 ZPLD1 AC100800.1 HSPB6 AC078883.1 MYL4 IGKV1OR9-2 ARHGAP11A AC116158.3 KLK14 DOC2GP MEX3B DEPDC1B S100P CYCSP6 ZNF85 MMP13 AC048382.5 RHOXF1 HOXC13 FAM151B ZBTB34 FOXQ1 SYNE2 AC024560.5 DOCK2 AL357054.4 AL117339.3 MYO3B GIMAP8 PPM1J C6orf223 FAM95B1 CSP2 NLRP3 NPM1P25 ZFP3 ST6GALNAC5 AC006130.3 SFXN2 RFC3 MYOM1 CD33 AP003071.5 AKR1B10 NXPH3 LINC01920 AL691442.2 FARSB TGM2 SLC22A18 CLDN11 AL162741.1 AL109804.1 LINC00492 ARPP21 TMEM86A CIB2 PHACTR3 AL627309.3 AC005045.2 RPP40 AC092656.1 LINC01127 SYNGR3 APCDD1L SHISA3 WDR54 LINC02027 ELOVL3 NPM1P9 SLC2A13 RERG AC068580.3 GALNT13 AC096751.2 FO681492.1 CCDC141 AC005865.2 LINC00514 AL359922.3 AMN1 AC022973.5 AC066580.1 CMTM1 AC078860.2 MUC3A LINC00173 SEC16B AC011405.1 SLC16A12* |

| | | |
|---|---|---|
| | | *AC069360.1 ALK PLEKHB1 GRB7 AL031320.2 LMLN ATP1B1 FAS HMGB1P5 GOLGA2P5 IDI2-AS1 SLC2A1-AS1 TRIM31 PM20D2 AC023024.2 SPRN REP15 HSD17B1P1 SEMA3E CLEC2B TNFSF10 DNASE1L3 AC011462.5 CHEK1 AL136964.1 NEIL3 KPNA2P3 SCIN AKR1C7P KIF11 RASD2 PART1 PARVA FAM72B SLC40A1 NOVA2 AL133477.1 CADM2 CD36 ESRG HAO1 TTLL1 MUC7 PSG8-AS1 CENPH TMPRSS11E KIF18B AC232271.3 PCAT5 TLR6 GPI ASF1B SMIM10L2B PACRGL ADORA2A STMN3 GRK3 KCNH2 STAT4 CDK6-AS1 FAM216A FAT2 E2F1 MIR583HG DUOX2 RASGRP2 AC046130.2 TSC22D3 CDC25C IFFO2 PTRH1 ZNF382 MROH7 LINC01116 DEGS2 ITGA1 CCDC83 ARHGAP33 AC008522.1 NOTUM SNRPF PLCH2 PRRG4 RPL12P21 AC068831.6 AC008494.3 BAG1 GRIN2C PPIAP22 COLGALT2 AC060834.1 PAPPA NEAT1 KIFC1 RAB26 AC016866.3 HRAT92 ZWILCH TP73 KLHDC7B-DT BMS1P13 AKR1C2 CACNG4 MYBL2 COL4A3 CCDC13-AS1 AC091057.1 MASTL AC009055.2 AC145285.3 HSPA4L AL161756.1 LIPH MT-CYB MYO16 AC106886.3 CENPO KIAA1755 AC025419.1 MGAT5B EGR1 TEX13D LRRC31 CNGA1 MIR3681HG SNX31 TEKT3 DLX4 TUBA1B KLRC2 PCDHA2 VILL ZCCHC24 PLAGL1 LY6G6C PIM1 AC079601.1 C4orf36 ITGB7 AC010247.1 C11orf86 ASAP3 POLA1 TM7SF2 ARHGAP28 CDCA7L JMJD7 OXCT1 LRRC66 AL451064.1 NR2C2AP INGX MCM3 VPS9D1-AS1 HECW2 CYP2A6 LINC00894 RCOR2 AC069499.1 BNIP3 HMGB3P7 LEF1 BICDL1 AP003327.2 SERPINB2 AOC3 FABP3 TRPV2 CCN2 BLID AC015712.7 AC146949.1 CTSS SLC25A13 LRRC29 CBWD4P ANKS1B AC008397.2 CNTF PPIAP45 LINP1 KIF20B RAD51-AS1 LINC02246 GAS2L3 UNC79 H3C4 BLM PLA2G4A AC011294.1 SMOX DNLZ LINC02709 MIR100HG AC011632.2 RPS2P46 ITGB6 PDGFB VIM NCAPG2 PARTICL AC027228.2 RGL3 RNF112 ABCC3 XRCC3 SAMD9L PCNA GPATCH4 MMACHC PIK3CD-AS2 AC133435.1 AL031963.2 BTBD9-AS1 EDNRB SCD NPM2 CD207 PHF19 CTD-2201I18.1 CCDC134 AC005775.1 DCST2 RPL21P40 PCDHB4 CCL20 AP000317.2 PARPBP MYLK4 STX11 SPIN4 CYP7B1 GPR176 ALDH1B1 LGSN ARMC4 IDI1P1 FAM86JP ACOD1 CEP83 KIF2C EGFLAM GUCY2D CYB561D2 CPM AC006299.1* |

| | | |
|---|---|---|
| | | *AC008147.2 AL390726.4 CDC42EP2 MT-CO3 TTLL11-IT1 PLD5 FBXO5 GBP2 MRC2 LINC00393 CCR5AS RGCC WDR4 ACVR1C EEF1GP4 DPH6 HIRIP3 AC093330.2 TICRR CLEC18B CTH DLG1-AS1 MEI4 RBM46 AC004982.2 LAT2 DAPK2 EREG CENPA SELENOM HAPLN2 TMEM75 CDCA7 THG1L TBC1D26 ABCG4 DPY19L2 OGFRP1 CLMN CYP2G1P SMILR AL355612.1 AL133390.1 CENPN ACP7 AL353708.3 UGT1A6 FAM30A STARD13-AS UTP14A PDZPH1P HPGD PLEKHH2 CCDC40 TUBB3 THOP1 ADCY8 TADA2A MTND5P11 KRTAP5-AS1 RBPMS2 SPRR1A LHPP AC105020.1 RAB36 MEIS3 PLK4 AC112236.1 RXFP1 NADK2-AS1 AL356489.2 ZNF724 HAUS8 IGFL2 S100A8 DYNC2H1 INHBB WDR7-OT1 MIR3150BHG GLYCTK SOX9-AS1 AC025279.1 MIR193BHG SLC6A14 ZNF503-AS1 Z94721.3 DHRS3 HOXD1 POLQ LINC01235 EIF3EP1 PBK AL031118.1 SLITRK6 COX6B1P2 AC087521.2 AC010132.4 GK-AS1 ADAM21 GDPD1 RAPGEF4 C20orf202 AADAT RGS9BP PRR11 AC007326.4 SKA1 ATG9B LINC00922 AC004801.2 NIM1K RAB4B-EGLN2 TRIP13 AL136171.2 AC127526.5 AC069209.2 NFAM1 Z84488.1 IL6 AC095057.3 SNAI3 AC011043.1 TROAP MIR6089 HMGB2 FAM242C AC104777.1 CDC7 AC004974.1 CASKIN1 TAS2R14 UGT2A1 FBXO43 MAST1 CEP164P1 AC002525.1 AC005332.2 TMPRSS15 EEF1A1P11 LYPD1 MX2 ISYNA1 ZP3 RAB39B NXT1-AS1 CREB5 ITGB3BP LINC01559 B3GAT2 DUOX1 ATP6V0A4 PARP1 ACSS2 PLIN1 AC114271.1 ACE GCNA IRF4 LINC00433 DACH2 SKA3 NCAPH SRCIN1 TAGLN RDH10-AS1 AGAP14P COL3A1 TK1 LETM2 AL049869.3 AL162231.2 DNAH10OS SGPP2 CD37 DPT RFPL4AL1 AC024587.1 AC090616.6 TBL1X EPHA3 TREX2 TMEM45B CNNM1 TMEM150C PLA2G2F PCDHB7 AL390195.2 MCPH1-AS1 SMC2 AC092718.6 RMI1 COLCA2 SHCBP1 PECAM1 CD27-AS1 MKRN2OS ARL9 AL355315.1 TBX4 YPEL4 SACS ASPM UBE2S AC133644.1 AC008966.2 RFC2 STARD6 PPARA AP000311.1 Z95118.2 RN7SKP23 AC117464.1 AC016590.1 TMSB15A KCNJ1 EBF4 SCARA5 EXOSC3P2 HTR2B PRIM1 CYP1A2 TNFSF4 LINC01287 AL589743.1 AL451054.4 NAP1L3 CDCA3 AL663070.1 TCP10L SPATA25 PIMREG AC009879.3 RALGAPA1P1 S100A4 LGMN PPEF1 NOP56 HTATSF1P2 ATAD2 NUDT11* |

| | | |
|---|---|---|
| | | *KNL1 ZNF229 CHRNA1 SLC2A12 DNMT1 KLF8 AC004982.1 AC132153.1 BRIP1 FAM172BP AC131009.4 AC011043.2 COL4A4 GUCY1A2 NPC1L1 KREMEN1 STIL NME7 ENPP5 UBE2C CYP3A7 FFAR4 AL158206.1 AC013394.1 KCNAB2 SRRM3 GINS4 AL583810.2 MIR924HG MYO15A SBK3 IL1R2 TTC28 COLEC10 ANKRD30B EVA1B EFHB PCDH19 STRCP1 POLE2 ARHGAP19-SLIT1 DAZL AC099489.1 PLCL1 IGFBP1 TCAF2 GGT7 HPCAL1 AL670729.2 C11orf87 AL139407.1 DOK3 PXDNL FIGNL1 AC110015.1 AC011374.2 TNFSF13B AC011503.1 RAB40A PUS7 TP53INP1 WDR35 RIBC2 TBC1D10C LEMD1 LINC00461 HNRNPM AL356274.2 SPRR2A IDI1 ULBP2 AUNIP SPIRE1 OR2A42 CORO1A RRM2 NAP1L4P1 AC015849.5 LINC02873 EPHX1 C19orf48 RASSF2 TOP2A ADAM1A FEN1 PPIF JSRP1 KLK5 AC242376.1 PURG AC005410.2 AC097637.1 CXCL9 AP002387.1 ABAT ATRIP HELLS CASC17 RAPGEF3 FANCI DRD5P2 DBF4B AL109976.1 CCL28 RAD54L JAM2 ZNF367 CYTOR CORIN C4orf47 PLEKHG6 NEXN PLEC LINC02009 ITGA9-AS1 TPO AC135782.1 TEX41 AC079385.3 WNT2B AL391427.1 MPZL3 CPHL1P SMOC1 ABCB4 ARHGAP31-AS1 SPC25 FAH WDHD1 AL590062.1 DNTTIP1 AC097639.1 IKBKE TRAF1 TMED7-TICAM2 PKMYT1 ABHD12B DUT SMIM14 KIF18A LINC02615 LINC00052 C16orf46 FER1L6-AS2 SEMA4G LIPE-AS1 TMEM170B KRTAP2-3 KIF15 AP001486.2 AC006511.3 SPRY3 INMT DYNC1I1 PADI2 ADSL STAU2-AS1 TTLL3 PGM5P3-AS1 KRT16P3 GLIDR AL359834.1 ERFL PGAM1P5 AC112496.1 RRM1 ATP6V1G2-DDX39B OLFML2B FAM189A2 WDR76 GPC2 HJURP AL359258.3 AQP1 ST6GALNAC6 SNAI3-AS1 SYCP2 ACER2 KIAA1614 IL7R KIF24 ZSWIM5 TLR4 SPDYE9 AC105460.1 MS4A7 AC016766.1 AC021945.1 SLF1 HFE AC097451.1 POU2F3 AC117481.1 AC010931.1 CD96 CCDC15 AC015818.9 MGC32805 AL512353.2 CSNK2A3 AC139720.2 DTL PCDHB3 AC120036.4 FAM83D CR392039.3 CREBRF ST6GALNAC2 AC005332.7 TMEM213 MIR205HG IL33 AC004593.2 C15orf56 PLLP MSLN AL359715.2 HHIP LINC02048 AC104825.1 RAD51 CIT PAICS ANGPTL2 AL049634.1 AC145423.3 SERPINB10 PROX1-AS1 AC005041.1 PCDHA1 HMMR JAK3 CCDC146 TCTN2 AC010168.1 AL031846.2 HDHD5 CAMKV AC090527.3* |

| | | |
|---|---|---|
| | | *NCMAP CHAF1B SCNN1A Y_RNA FABP4 SLC35E3 TEDC1 HMX3 LINC00378 LINC02099 AP002992.1 NPTX1 PCDHA8 ZNF488 MCM6 SYT17 MIR4432HG CCDC171 AC007106.1 ERVH-1 AC015802.1 CROCCP4 EPHB6 CU638689.4 GPRIN3 CXXC4 RP9P WDR18 GIMAP5 GMNN FAM114A1 AP002784.1 KCTD16 ZNF295-AS1 SNRPCP1 AC114803.1 AC023157.2 ERCC6L SIRLNT SHISA2 TCP11L2 AC006460.1 MROH7-TTC4 MLXIPL AL138828.1 ORC6 KRT5 LINC02245 AC114811.2 AC124290.1 CDH12 C3orf35 LINC02827 LINC00911 MAP7D3 FCRLA LINC01433 RUSC1-AS1 AC013268.3 TLL1 PIGW AL355297.3 PLAC4 EXOC3L1 DUSP16 AC008537.3 SAMD9 HSD11B1 TRBC1 AKR1C3 LINC01234 CLUAP1 NTN5 GPR87 CTSH DAW1 AC006960.3 NRAV EXOSC6 ANKRD1 SAA2 TNFSF9 CNIH3 PAQR9 LINC00702 NXPE1 MEIOC LINC02029 PRICKLE1 RBFA KCNK5 AC006486.1 RNVU1-26 BRD3OS HNRNPA1P33 UHRF1 RIMBP3C NECTIN3-AS1 SGO2 SLC2A9 CHN1 AC016245.2 AC100822.1 PHOSPHO1 CHAC2 AC067751.1 AC099548.2 AL354893.2 ACOX2 GEM SLC14A1 NANOS1 AC109322.2 KCNJ15 ATAD5 NOTCH3 RPL9P25 QTRT2 OR8S1 TTK PSMB10 TRNP1 MYH7B SDCBP2 AC069281.2 PLXDC1 KIF3C AC008543.1 CDKN3 GAPLINC AC073349.2 AL669831.7 FJX1 AL357558.2 AC026471.4 AP000696.1 SCG5 NCAPG RNF128 PCED1B-AS1 AL353132.1 USP44 WHRN AL360181.2 LINC02742 GCNT1 FDPS DUSP9 C21orf58 VAV3 AC234772.1 MIR4458HG RMI2 BPTFP1 AF121898.1 LNCPRESS2 CRACR2B H2AC20 COQ2 SPRR2E HCG20 VRK1 ADD2 CNTN6 CYP3A4 TUBB4A SATB1-AS1 SKA2 CENPM DPYSL3 FANCB EVX2 RBL1 AC008067.1 CCDC189 SLC51A Z84485.1 LINC00242 AL121839.2 AL049796.1 CENPF MS4A14 H1-10-AS1 NUSAP1 AP000697.1 LINC00470 AP001120.2 CEACAM3 C2CD4A FLRT1 DPCD FTH1P11 KLHL24 AL589743.4 ARNT2 POLR3B AMZ1 DHRS2 AL163540.1 KCNA7 MISP3 AC022387.2 CDCA8* |
| Unique to DU145 line | 1388 | *CREB3L1 AC124068.2 PSTPIP1 XDH MGC16275 AL136985.3 AL355499.1 H3C12 SNHG16 PTPRR SLC52A1 YBX2P1 AC058791.1 GAS5 TMEM139 AC091982.3 AC138969.2 CEBPA-DT TECTA CNGB1 NOS2 GZMM ZBTB12 UPK1A LINC01569 LRMDA AC008946.1 ABALON KCNK3 GDA AC092849.2* |

| | | |
|---|---|---|
| | | *AC005674.2 AC089983.2 RPL4P6 SPN AC091181.2 GRM2 AC006008.1 LINC00706 ALOX15P1 AC108448.3 AC138466.1 AC007389.1 AL451074.6 AC020907.5 CACNG5 AC104109.3 POU3F1 MAFA-AS1 AC007375.3 CPNE5 CD160 SEC14L5 COL1A1 AC005540.1 BISPR ALDOC AC018529.1 GDPD5 SSTR2 AL359881.1 EPHX3 AC009275.1 RNF223 AL137786.1 VWA5A GFY AC233699.1 AL133476.1 CD38 KLK8 AL031719.1 C11orf88 ROR2 AC005962.2 GASK1B RIBC1 AC004801.6 H2BC21 C3 HTR7P1 AC008966.1 AL358473.1 LINC01843 AP006222.2 INSRR AC092944.1 AC023790.2 ATOH8 ASCL5 AC104596.2 IDH1-AS1 AC097059.1 MYH6 AC133644.2 SAA2-SAA4 NFE4 FBXL19-AS1 AP000866.1 AC022154.1 HESX1 AC084757.3 TMEM156 AC006449.5 QPRT MYADML2 C1QL1 AP002990.1 AC091057.3 H2AC8 AL355337.1 AC018553.2 HLA-DRA AC026464.4 ADRA2A TERC CNOT6LP1 RAG1 IRS1 PTPN5 AC013472.2 NME1-NME2 LINC01270 SEPTIN4 AL049555.1 MT3 KCNE3 AC007842.1 SCAT8 AL353705.4 RIIAD1 NCAM1 AL137060.3 AP002847.1 FRY-AS1 ADGRG5 AGT CLSTN2 TSPEAR-AS2 NACAD CALHM5 GDAP1L1 TMEM125 CNTN2 AC104850.2 TM6SF1 UBAC2-AS1 H2AZ2P1 PSG3 ADGRD2 METTL15P1 AC073365.1 ADAMTS2 PSG4 AC090061.1 AL122035.1 TBX2-AS1 SMTNL2 AC105046.2 C19orf67 AL049838.1 AP002754.1 C2orf92 PTGER1 KRT15 GFI1 GATA1 HK3 AL442224.1 KRT17 AC019069.1 ACTG1P1 GAPDHP70 TCEA1P4 XCR1 PLPP7 LINC01058 PLXNB3 SH2D2A LINC02204 SORCS2 CCNP AC114490.3 PCBP3-AS1 AL022069.2 AC120057.3 SCN1A BTK AP005018.2 MMP23B KIF19 CSTA ZCCHC12 C2orf78 TMEM25 ST7-AS1 BMP6 SCN3A CAPN6 RPE65 AHRR AC124862.1 AC131392.1 LINC01607 KLHL6 NEPNP LINC02004 VSTM4 GPR158 LDLRAD2 UBE2D3-AS1 HOXB8 WFDC21P AC026150.2 MT1DP IGSF9 BX470102.1 NGFR ATP8B4 COL23A1 DOCK8-AS1 FP236315.2 CACNA1H AL117379.1 FOXD4L6 PSD2 LINC00636 FAM3B ANKRD20A2P RPS20P22 COL5A2 SIX2 AC008764.4 AC068338.2 AL590714.1 ANXA8 DCT HLA-DOA DLGAP1 MIPEPP3 XKR9 AOC1 AC008770.3 DRC7 PCDHGA10 TYRP1 CD177 BX255923.2 ROPN1 AP001830.1 RHCE SENP8 TCEANC AL512604.2 AC012184.3 AL662884.5 AC005537.1 AL353743.2* |

| | | |
|---|---|---|
| | | *RGPD2 HS3ST6 AL158166.2 PRSS30P AL137784.1 AC018926.3 FAM81B PHOSPHO2 TREML3P CFHR1 GPR176-DT SLC25A34 SLC7A4 AL138781.1 MAP1A KLK4 H2AC13 BX537318.2 ZNF781 PRDM6 EPHX4 PSMB8-AS1 INCA1 SIGLEC1 LINC00324 CCNT2-AS1 LRRC19 KCNN3 LMNTD2-AS1 LST1 TCF15 HDAC4-AS1 WIF1 TPPP3 CHMP3 CD79B ITPR1-DT TNFRSF19 AC226118.1 CPA4 SDK2 BAIAP3 RBM3 ZNF807P KCNK7 AC092687.3 AL591043.2 CNTN5 NPNT AC022532.1 TNFRSF10C MEF2B CAND2 OTOF AMIGO3 OSTN AC138811.2 RAB25 LINC00896 BSN-DT AC061992.2 DMBX1 ACTRT3 SHANK2-AS1 APEX1 AL035458.2 CAVIN2 AP000688.1 PRSS43P PGLYRP4 ZBED6 KRT18P63 LINC01213 MALL C19orf38 XIAPP2 PIANP PFN4 GTF2IRD2P1 LINC01600 TRAPPC6A TMLHE-AS1 CEMP1 SLC23A3 AC007881.4 AC127024.8 SLC11A1 POU2F2 AP001574.1 ADAMTS17 AC080038.3 CLCNKB FLJ31104 AC004832.3 ADCYAP1R1 AC122129.1 CPZ GRAMD2A HLA-DQA1 DHFRP1 AC012213.5 TMED2-DT LRRC36 SNTG2-AS1 AC013270.1 AL031777.2 AC010331.1 NCMAP-DT FMR1-AS1 AC005618.4 PSG7 STK32A-AS1 KCNH1-IT1 AL512625.3 ADAMTS12 KLHDC1 ZNF608 AL158835.1 AP000350.6 RASSF8-AS1 CABP7 KLHDC8A HORMAD2 AC004408.2 GREM2 SYNPO2L AC073065.1 CFAP70 AC254562.3 C4B AL136084.1 AC118553.1 PDZK1IP1 COL9A3 HES2 TPTE2P1 AC135178.4 MDGA1 KCNH3 DBH-AS1 LINC00652 C1S LRRC63 AC026786.1 SERPINB8 RPLP0 CSDC2 AF064858.1 AL359538.1 HMGN2P5 AP001347.1 KIF1C-AS1 LRRC32 SLC29A4 CKMT1B TMCO1-AS1 F13A1 LINC02432 AC079880.1 ZNF365 AP003119.1 ANGPTL4 CYP4F22 TAS2R5 ESAM MT1E AC008691.1 AC008763.1 NTRK2 FAM90A20P AC025262.2 TAS2R4 CES3 Z83843.1 CA4 IRX4 KRTCAP3 CTRC MSH4 RXFP4 RIPK3 AC020922.1 SLC8A2 AP000365.1 AC087893.2 CFAP91 PLAT TAP2 AL096794.1 AC022966.1 LHX4 RASL12 CASC15 AL391280.2 RB1-DT ADAM28 AC135983.4 CYP4F2 AC010522.1 AL021392.1 AC245052.4 NUTM2A CXCR3 AC012306.2 NAIPP1 GPBAR1 H1-12P LCN10 ASPG PDLIM4 AC079753.2 SLC38A3 SOHLH2 LINC01635 NAT2 WDR87 FAM138B AC020661.4 HLA-DPB1 TSPEAR-AS1 AKAP3 SLC22A13 NPIPA8 CAPN11 AL022326.1* |

| | | |
|---|---|---|
| | | *FAM90A11P AC005089.1 NOX5 C1orf167 AL118522.1 H4-16 H3C2 WNT11 AP005242.4 GPR35 COMP AC105020.4 AC016877.1 CKMT1A TUBA1C PTPRG AL844892.2 PATL2 AL357556.4 CRIP2 AC025283.2 AP000346.2 DIO2 GPER1 SYT8 ANKK1 RPL6P27 GAS2L2 PLA2G7 ARL5C AL359313.1 PDE6B GRPR LINC01018 AC079414.3 AL110118.2 ZNF853 AC015909.2 H2AC11 CCDC184 SMIM6 ANKLE1 ANKRD20A17P ILDR1 AC009486.2 COL16A1 INHBE AC023794.5 MRGPRE ZRSR2P1 ITGAX VASN LINC01271 LINC00659 AL024497.1 ODC1 CYSRT1 TCTE1 KRBOX1 BCL2L12P1 TSPEAR CCDC180 AC125611.3 AC015971.1 RASGRP4 LINC01275 TNFRSF18 AC097382.3 AL022332.1 DSCR9 KIF1A DNAH8 ICAM3 AC024580.1 PLEKHD1 SPAG8 SAA4 BX322234.1 AC026471.2 AC096992.2 AL021154.1 LINC01952 AC007569.1 AP001972.5 AL135999.1 LINC01583 LRRN4CL AC116351.1 AC009292.1 AL356740.1 AL158151.1 HCN4 CPB2-AS1 AC244453.2 TRIML2 MADCAM1 MEI1 AC091114.1 OLFML3 CPS1 NDRG4 FBXL21P RPP25 TNRC6C-AS1 LRG1 FAXDC2 AC010643.1 AC007066.3 AL590556.3 RNU1-1 ADCY4 AC016590.3 LINC02263 AL445524.1 DOC2B PTGR2 CD22 LRRC46 RORC LDLRAD1 SEMA4A LINC00634 AL137002.2 POU5F1 MDP1 LINC02262 ANKRD33 AC141586.4 CCDC187 FOSB TUBB8 FBXW11P1 SUN3 IL1B UBE2Q2L NHLH1 LINC02453 RPL23A AL049840.2 AL133500.1 DMWD CSTF3-DT RAMP2-AS1 PICART1 SOBP ITGA7 COL24A1 U1 IGFBP3 AC012508.2 AC254629.1 TTC9B AC079174.2 AL121917.1 AC024941.2 AL121938.1 JMJD7-PLA2G4B WASF5P AL356017.1 LTK YARS1 AC018647.1 CCDC63 KISS1 PGAM2 CCN5 AC093752.2 LINC01135 GDF7 PSG11 PSAPL1 AC244517.6 TBC1D3P1-DHX40P1 AC002451.1 IFITM1 AL137000.1 AC005041.3 SERPINA3 TSHR Z85996.2 SPTSSB KLHDC9 AP000229.1 NALT1 ACTG1 C9orf147 FP671120.4 IMPDH1P5 AC000120.4 MAP2K3 Z99943.1 MESP2 BHLHE41 TRBV20OR9-2 KRR1P1 AC117500.5 AP005131.1 AC005225.3 SERPINA5 AL358472.5 AC012236.1 PURPL FHAD1-AS1 AC104785.1 AC087175.1 ALPL CACNA1G SPHK2 AC008532.1 AL603832.2 RUBCNL ACTBP13 SLC6A3 TMEM81 NRXN2 AC099552.5 ACKR3 AC084880.1 AC004784.1* |

| | | |
|---|---|---|
| | | *AC096677.1 DUSP13 AC079807.1 EFS LINC02856 LYPLAL1-DT SLFN12L IL20RB-AS1 TUBBP1 MAST4-AS1 ABHD1 DRD1 RAP2CP1 ST8SIA5 PTGER3 RFLNA ERBB4 AC116351.2 TSPAN11 AC060766.1 SCML2P2 ADRA2C ANKRD34B AC093827.1 SOX7 CXCL14 MATK AC079296.1 RENBP AL137847.1 AL390728.2 FCRL6 ITIH5 CAPN3 AC036176.1 HMGB3 LINC02274 PADI3 ZNF843 KCNE1 AC061975.6 AL596244.1 AC105339.2 UNC5B NXNL2 AC117500.3 ALPK3 AP000569.1 ALOX5 TRIM22 FKBP7 TRPM8 FGFBP3 H2AC14 RGS16 AC108047.1 ACVRL1 H2BU1 FCGR2B CSPG4P5 CA14 YBX2 LINC01772 ARHGAP30 PIGZ CREB3L3 AC068020.1 ZCWPW1 AC108471.2 SH3GL1P2 AC104564.2 CU634019.3 AC008571.2 YOD1 AC089984.1 CALML4 CFAP46 GPR89P LINC00482 AC005840.2 TTLL6 SYT2 RASL10B AF131216.3 GUSBP3 CEACAM8 HIF3A LINC00484 TEX19 NDUFC2-KCTD14 AL035530.2 CHST1 AC006252.1 LGALS1 COL15A1 NPAS1 AC091152.2 DACT3 RAB37 LINC01504 FRRS1L AL731702.1 LBHD2 POU2AF1 PNMT SOD3 AC139530.2 AL590428.1 DNAH6 NUTM2D TAS1R3 OR6L2P SLC2A3 LRRC75A AL645922.1 TNFRSF11B AC002996.1 EFHD1 AC090541.1 RNF180 QARS1 CAPN14 CTBP2P10 ALG14 LINC01686 LINC01391 RPS27A NTNG2 ADCY5 MROH8 AL158801.2 NSMCE1-DT AF111169.1 PIGR AL445250.1 ZNF311 TRO AC064843.1 LINC00649 PGGT1BP1 RSKR LINC02593 FOLR1 PRAF2 AL391056.1 CADM3-AS1 AL359962.3 SGIP1 MAL RPL7AP15 AC118754.2 MIR1-1HG-AS1 CACNA1B LINC01366 LHX5 AL133255.1 AL139142.2 MT1M DLL1 TRG-AS1 SERPINA1 LIF-AS1 AL035460.1 AC100830.3 OLFM1 RET RPSA AL136379.1 IL1RL1 AC073195.1 CYP4F3 AC020550.2 MAMSTR MTTP TSHZ2 RPL22L1 AC136352.4 EFNB3 DUSP26 FAM215A LINC02458 CTBP2P9 AC009403.2 MYT1 MFRP TRAPPC2B AC016727.1 NR5A1 TPBGL SLC25A21-AS1 LUNAR1 SCUBE1 CCL27 AJ011931.1 MYBPH ERN2 AC080038.1 AC009065.4 AC009053.3 TNFRSF4 AC130650.2 AC002378.1 AL033397.2 STAC2 AL139420.1 KCNJ9 C2CD4C CHL1 AC008736.1 LCK TDGF1P2 P2RX6 AC136475.3 TIGIT AC108471.3 AL353588.1 ZBED5-AS1 AC004951.4 HLA-DPA1 AC106038.1 HOGA1 AC008393.1 EIF4BP7 TCEA1P2 H4C5 RPL4P2 FHL2* |

| | | |
|---|---|---|
| | | LTB S100A14 AC092111.2 KCNK17 CERS1 TMEM191B BEST1 ACRBP LZTS1 RHBDL3 AC008147.4 PCDHB17P MORN3 TECRP1 RSPO1 ADAMTS14 LINC01892 HR FP671120.5 AC022613.2 SLC38A2 FAM83E LRRN4 SPATA41 SLPI AC010754.1 AC087164.2 FAM220A H19 PSG5 PAK3 SMCP TREML4 LINC01715 AL445928.2 VTN GTSE1-DT AL355601.1 LINGO1 KCNG2 AL353572.4 LINC01767 CRLF1 ABI3BP OPRL1 AC087623.2 AC055714.1 HTRA3 PAPPA2 CU638689.5 C1orf210 CGB2 SYCE1 IL17RB LRRC15 DIRAS2 AC010336.7 HAR1A MUC20P1 GSTT2 AC025423.4 OTP JPH4 AC007308.1 AC010240.2 RPL13AP5 TM4SF19-AS1 SCTR-AS1 SRGN CRYGN AL139094.1 AC025569.1 AL157400.3 AC055811.2 FUT8-AS1 EEF1B2 DNAJC12 AC021739.2 SEMA3B SLC24A2 BRWD1-AS2 PER3 PSG1 S100A6 RPL6 RHCG SLC7A10 LRRC10B LINC01303 KCNK12 MT1X MIAT AC003965.1 SULT4A1 ARRDC4 SRSF9P1 LRRC73 IER3-AS1 AC137630.3 EXOSC5 AZU1 AL645940.1 AL135978.2 ACHE AC004834.1 FOXD4L4 INSYN1 HEG1 AL356417.3 AC022826.2 TXNIP KRT81 FADS6 ANKRD31 C16orf86 AGAP2 RNVU1-28 FOXA3 LINC01623 Z98752.4 KRBA2 NEBL-AS1 AC005993.1 DUXAP10 VIPR2 UFC1 AC107021.2 AC079848.2 AC139099.1 PSTPIP2 LINC02600 TAMALIN SAP30L-AS1 RNU1-47P AP005717.2 RHEBP1 OR11M1P H4C9 L1CAM AL391832.3 AL157996.2 GOLGA8M KLHL7-DT AL157996.1 AC018470.1 EPPK1 ADGRD1 SPAG17 ZDHHC11 AP000640.1 PDCD4-AS1 AL022069.1 AC007342.7 B3GALT4 KIF26A AC127070.2 BDH1 AC119674.1 AC016877.3 AC005332.1 KRTAP5-1 AP002478.1 PCDH1 AC023389.2 SREBF2-AS1 AC119403.1 PMEL CHP2 LSP1 ALOX15B AC005837.1 AL732292.2 SLC22A17 CDYL2 MAOB PTGDS BST2 LYRM9 H2BP1 AL355377.4 TG DRC1 AL360268.1 AC100835.2 AC004069.1 RPL41P1 AGGF1P1 RPS10P7 EPN3 RARRES2 ICAM4 PIWIL4 SLC5A5 CFI PSMC1P1 PSG2 ADRA2B GAL3ST2 REG4 C19orf71 AC015712.2 BX322562.1 PIK3CG FGF7 SLC15A1 IL17REL TAT SNHG5 MB LINC02478 AC025774.1 AL929091.1 GPR4 SKOR1 CCDC18-AS1 SAMD14 SLAMF9 MAG HCN2 AC243964.3 HES6 SEMA6C AC005020.2 AC006435.4 HAND1 HPN-AS1 AP001267.3 FOS CHRNA4 CLEC2L AC026691.1 SCN2A UPK3A TREHP1 LRRC74B FOXL2 |

| | | |
|---|---|---|
| | | *HTR6 AC011466.4 KCNQ2 SMC1B LINC01363 AC098934.1 AC117488.1 PPP4R1L LINC02562 HK2 AC106886.2 AC020658.6 KHDRBS3 AL022341.2 OR7E125P COL14A1 KLRC4-KLRK1 SOX8 KLRC3 AC087591.1 AC129492.5 RN7SL1 AC008013.1 AC008403.2 LINC00942 AC108925.1 TNS4 AEBP1 ISM2 ZNF256 AL450311.1 C20orf144 CACNA1S AL080276.2 LRRC7 PCDHB16 NAIPP3 AC009093.8 AC020922.3 AL139156.2 AL353796.2 USH1G AL512329.1 KCNK13 MAP2 GDF5 LINC01348 ANGPTL6 AC017033.1 KLF14 EIF3L LINC02731 NPPC METTL7A AC009812.4 B3GAT3P1 COX6B2 RASL10A AC009102.2 AL391845.2 AC126755.6 FAM243A KCTD19 NOTCH4 AC008878.3 SMARCE1P6 NDUFB2-AS1 LACTB2-AS1 AC087645.2 H2BC8 COL17A1 MAT1A LGI3 ACTB VN1R1 SLC49A3 SERPINE1 DNASE1L2 GLB1L3 AC132812.1 AC099552.3 GPR142 ARC SLC41A3 RPS7P14 SFTPA2 AC022919.1 AL451074.2 ATP6V1C2 AC010327.2 AL139424.1 CDH23 CHRD AMN ATP8A2P2 ITGA11 MIF-AS1 AL133492.1 HAAO ALPG RPL41 PARD6G-AS1 AF127577.6 AC148477.2 AC021016.2 KLF15 PPP2R2B AC084876.1 AC234582.1 AC017104.3 PSG6 CEP295NL HEY2 AC024267.6 TPRG1-AS1 CFAP52 LINC01634 AC233723.1 ITGA9 SCUBE2 ZNF286B NRCAM CFB VWA5B2 LINC00518 U73166.1 CRTAC1 AC079145.1 RAP2C-AS1 CCDC85A AL162632.1 AC015802.5 NES PITX3 CITED4 EDN2 GRM4 CACNA1G-AS1 AC138409.3 KLK1 PNMA8A ITGB3 CLRN3 GPR156 CFHR3 DUSP15 TRGV9 AL022344.1 ADAMTS10 BSND AL135926.1 ANAPC15 LINC02762 PRSS8 IL11 PDZD4 AC244230.2 AC120036.1 AC022613.1 SBK2 AC092142.1 COL5A1 AP002761.1 AL353608.3 HLX-AS1 C10orf82 INKA2-AS1 KLF10 TEPP FTCD AC020904.2 RPS9P1 CYP46A1 KBTBD12 DNER WNT9B AC010531.1 PTH1R RPL12P37 RPS14 ONECUT2 NRG1 TNFSF14 SYT9 ESPN AC006486.2 MYO18B AC139491.2 AC084035.1 AL049839.2 AP001981.2 AC024361.2 AC124312.3 AP001596.1 AC006059.5 AC093668.1 TAS2R20 AC104452.1 AL356215.1 GALNT16 SELENBP1 NEK3 AC139493.2 AC007405.1 Z82217.1 AC021066.1 BEX5 CACNA1I CSPG4P13 DACT1 LINC02324 AC091978.1 AC005670.2 AL136307.1 PAH PPP1R12A-AS1* |

| | | |
|---|---|---|
| | | *AC145098.2 LINC02610 UGT3A2 MMP9 SPOCK1 SPEF2 AL359636.2* |

**Supplementary Table 5. List of quantitative RT-PCR primers.**

| Target name | Forward (5'-3') | Reverse (5'-3') |
|---|---|---|
| ***EREG*** | CGTGTGGCTCAAGTGTCAAT | TGGAACCGACGACTGTGATA |
| ***IL6*** | TACCCCCAGGAGAAGATTCC | TTTTCTGCCAGTGCCTCTTT |
| ***CXCL8*** | GTGCAGTTTTGCCAAGGAGT | CTCTGCACCCAGTTTTCCTT |
| ***WNT16B*** | GCTCCTGTGCTGTGAAAACA | TGCATTCTCTGCCTTGTGTC |
| ***SFRP2*** | GCCTCGATGACCTAGACGAG | GATGCAAAGGTCGTTGTCCT |
| ***MMP1*** | GGTCTCTGAGGGTCAAGCAG | AGTTCATGAGCTGCAACACG |
| ***MMP3*** | GCAGTTTGCTCAGCCTATCC | GAGTGTCGGAGTCCAGCTTC |
| ***MMP12*** | ACACATTTCGCCTCTCTGCT | CCTTCAGCCAGAAGAACCTG |
| ***SPINK1*** | CCTTGGCCCTGTTGAGTCTA | GCCCAGATTTTTGAATGAGG |
| ***CSF2*** | CCCCAGTCACCTGCTGTTAT | TGGAATCCTGAACCCACTTC |
| ***ANGPTL4*** | GCCTATAGCCTGCAGCTCAC | AGTACTGGCCGTTGAGGTTG |
| ***IL1a*** | AATGACGCCCTCAATCAAAG | TGGGTATCTCAGGCATCTCC |
| ***IL1b*** | GGGCCTCAAGGAAAAGAATC | TTCTGCTTGAGAGGTGCTGA |
| ***CXCL1*** | AGGGAATTCACCCCAAGAAC | TGGATTTGTCACTGTTCAGCA |
| ***CXCL3*** | GCAGGGAATTCACCTCAAGA | GGTGCTCCCCTTGTTCAGTA |
| ***IL-2*** | TGCAACTCCTGTCTTGCATT | GCCTTCTTGGGCATGTAAAA |
| ***IL-3*** | CTTTGCCTTTGCTGGACTTC | CCGTCCTTGATATGGATTGG |
| ***IL-5*** | GAGACCTTGGCACTGCTTTC | CAGTACCCCCTTGCACAGTT |
| ***IL-12*** | GATGGCCCTGTGCCTTAGTA | TCAAGGGAGGATTTTTGTGG |
| ***MARCHF4*** | CATCGCCATAAGCACAAAAA | GCTGCAACCTGAACCTTCTC |
| ***SPNS2*** | ATTCTCAGCTGCGGCATTT | AGGACCAGCAGCCAGAAGTA |
| ***KIF20A*** | TGCTGTCCGATGACGATGTC | AGGTTCTTGCGTACCACAGAC |
| ***E-cadherin*** | TGCCCAGAAAATGAAAAAGG | GTGTATGTGGCAATGCGTTC |
| ***N-cadherin*** | GACAATGCCCCTCAAGTGTT | CCATTAAGCCGAGTGATGGT |
| ***Vimentin*** | GAGAACTTTGCCGTTGAAGC | TCCAGCAGCTTCCTGTAGGT |
| ***ALDH1A1*** | TGTTAGCTGATGCCGACTTG | TTCTTAGCCCGCTCAACACT |
| ***CD44*** | AGCAACCAAGAGGCAAGAAA | GTGTGGTTGAAATGGTGCTG |
| ***CD24*** | ACCCACGCAGATTTATTCCA | ACCACGAAGAGACTGGCTGT |
| ***RPL13A*** | GTACGCTGTGAAGGCATCAA | CGCTTTTTCTTGTCGTAGGG |

**Supplementary Table 6. Antibodies used for immunoblot (WB), immunofluorescence staining (IF) and immunohistochemistry (IHC) staining.**

| *Antigen name* | *Commercial source* | *Catalog number (clone number）* | *Application* | *Dilution time* |
|---|---|---|---|---|
| EREG | Cell signaling | 12048 | WB, IHC | 1:1000; 1:200 |
| CXCL8 | Proteintech | 60141-2-Ig | WB | 1:500 |
| γH2AX (S139) | MerckMillipore | 05-636-25UG (clone JBW301) | IF | 1:200 |
| p-53BP1 (S1778) | Cell signaling | 2675 | IF | 1:250 |
| p-ATM (S1981) | Cell signaling | 4526 | WB | 1:1000 |
| ATM | Abways | CY5207 | WB | 1:1000 |
| p-p38 | Abcam | AF869 | WB | 1:2000 |
| p38 | Cell signaling | 9212 | WB | 1:2000 |
| p65 (RelA) | Santa Cruz | sc-8008 | WB | 1:2000 |
| BrdU | Cell signaling | 5292 | IF | 1:500 |
| p-EGFR | Cell signaling | 2231 | WB | 1:1000 |
| EGFR | Santa Cruz | sc-03-G | WB | 1:1000 |
| p-mTOR (S2448) | Abcam | ab109268 | WB | 1:500 |
| mTOR | Invitrogen | AHO1232 | WB | 1:1000 |
| p-AKT1 | Abcam | ab8932 | WB | 1:300 |
| AKT1 | Cell signaling | 9272 | WB | 1:1000 |
| p-Mek1/2 | Cell signaling | 9121 | WB | 1:1000 |
| Mek1/2 | Cell signaling | 9122 | WB | 1:1000 |
| p-Erk1/2 | Cell signaling | 4377 | WB | 1:1000 |
| Erk1/2 | Proteintech | 16443-1-AP | WB | 1:1000 |
| $p16^{INK4a}$ | Abways | CY5357 | WB | 1:1000 |
| $p21^{CIP1}$ | Abways | CY5088 | WB | 1:1000 |
| His-tag | Abmart | M30111 | WB | 1:1000 |

| | | | | |
|---|---|---|---|---|
| E-cadherin | Proteintech | 20874-1-AP | WB, IF | 1:500 |
| N-cadherin | Proteintech | 22018-1-AP | WB | 1:200 |
| Vimentin | Proteintech | 60330-1-Ig | WB, IF | 1:1000, 1:500 |
| Lamin A/C | Proteintech | 10298-1-AP | WB | 1:3000 |
| Histone H3 | Histone H3 | 4499 | WB | 1:1000 |
| MARCHF4 | Bioss | bs-9338R | WB, IF | 1:500, 1:500 |
| CD81 | Abnova | PAB16754 | WB | 1:1000 |
| GAPDH | Proteintech | 10494-1-AP | WB | 1:2000 |
| β-actin | Proteintech | 66009-1-Ig | WB | 1:5000 |
| Caspase 3 (cleaved) | Cell signaling | 9661 | WB, IHC | 1:500 |
| Caspase 3 (intact) | Cell signaling | 9662 | WB | 1:1000 |
| PARP1 | Cell signaling | 9542 | WB | 1:1000 |
| Mouse IgG | Santa Cruz | sc-2025 | | |
| Rabbit IgG | Santa Cruz | sc-2027 | | |
